# Supplementary material for: Generative deep learning enables the discovery of a potent and selective RIPK1 inhibitor
Source: Nat Commun. 2022 Nov 12;13:6891. doi: 10.1038/s41467-022-34692-w (PMC9653409; doi:10.1038/s41467-022-34692-w)
Supplement: Supplementary file 1 — Supplementary Information [file 41467_2022_34692_MOESM1_ESM.pdf]

Supplementary Information

Generative Deep Learning Enables the Discovery of a Potent and Selective RIPK1 Inhibitor

Yueshan Li[1,3], Liting Zhang[1,3], Yifei Wang[1,3], Jun Zou[1,3], Ruicheng Yang[1], Xinling Luo[2], Chengyong Wu[1], Wei Yang[1], Chenyu Tian[1], Haixing Xu[1], Falu Wang[1], Xin Yang[1], Linli Li[2], Shengyong Yang[1,*]

[1] State Key Laboratory of Biotherapy and Cancer Center, West China Hospital, Sichuan University, Chengdu, Sichuan 610041, China.

[2] Key Laboratory of Drug Targeting and Drug Delivery System of Ministry of Education, West China School of Pharmacy, Sichuan University, Chengdu, Sichuan 610041, China.

[3] These authors contributed equally to this work.

*To whom correspondence should be addressed. Shengyong Yang, State Key Laboratory of Biotherapy and Cancer Center, West China Hospital, Sichuan University, Chengdu, Sichuan 610041, China. E-mail: yangsy@scu.edu.cn.

Table of contents

Supplementary Figures **1**
Supplementary Figure 1 1
Supplementary Figure 2 2
Supplementary Figure 3 3
Supplementary Figure 4 4
Supplementary Figure 5 5
Supplementary Figure 6 6
Supplementary Figure 7 7
Supplementary Figure 8 8
Supplementary Figure 9 9
Supplementary Tables **10**
Supplementary Table 1 10
Supplementary Table 2 11
Supplementary Table 3 12
Supplementary Table 4 17
Supplementary Table 5 18
Supplementary Table 6 19
Supplementary Notes **20**
Supplementary Note 1 20
Supplementary Note 2 21
Supplementary Note 3 23
Supplementary Note 4 25
Supplementary Methods **30**
Supplementary References **62**

Supplementary Figures

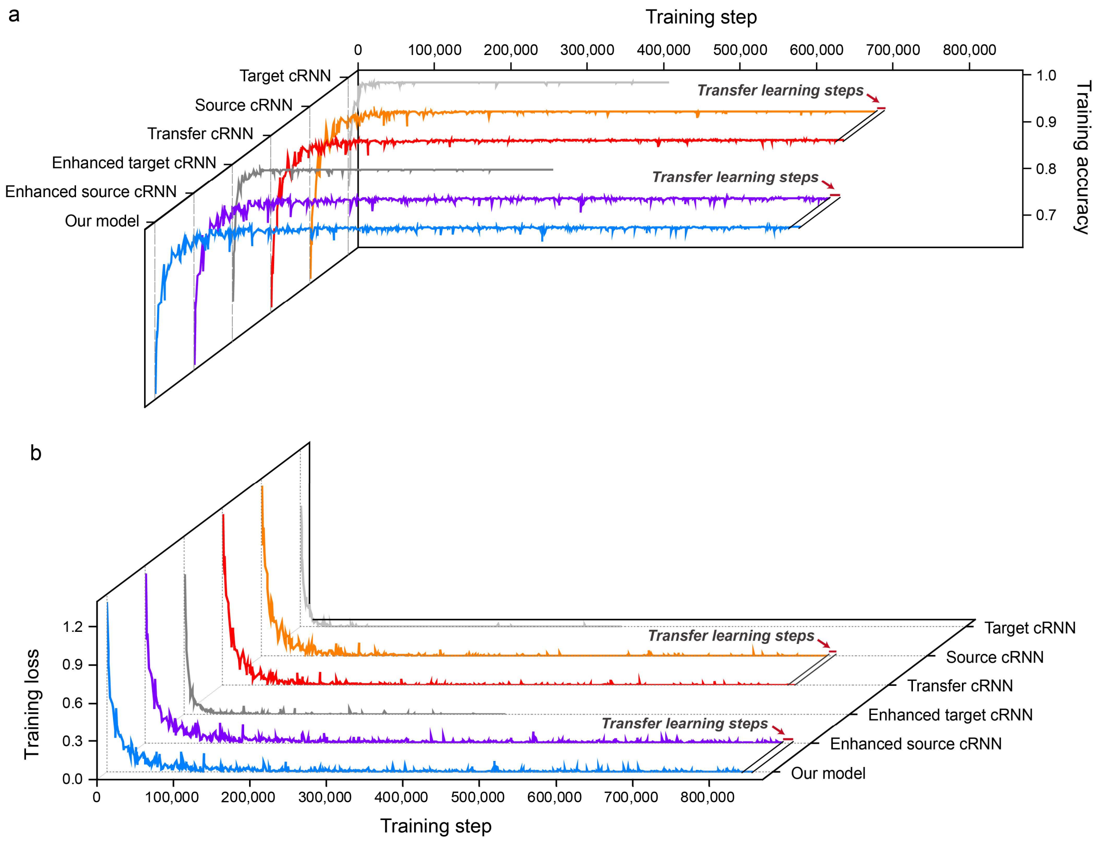

Supplementary Figure 1 | Training accuracy (a) and training loss (b) for baseline models and our GDL model.

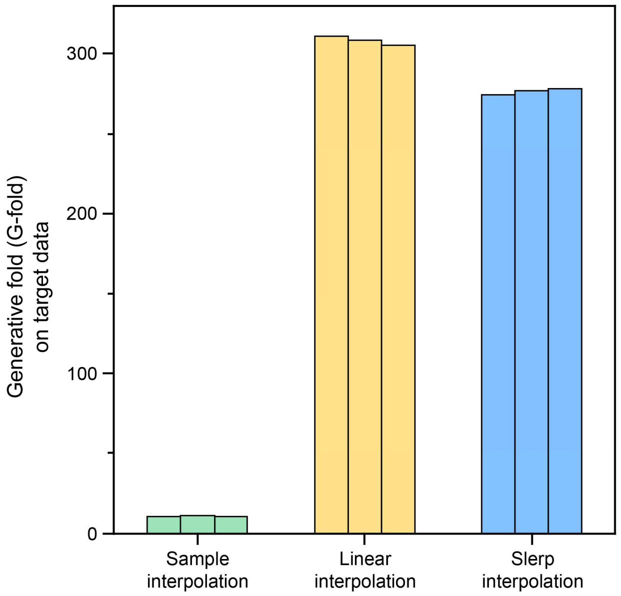

Supplementary Figure 2 | Comparison of generation capability of three different sampling enhancement types. Molecules are generated by our GDL model based on 100 molecules randomly selected from the target data, using sample interpolation, linear interpolation or slerp interpolation, respectively. The generation process was repeated three times. Source data are provided as a Source Data file.

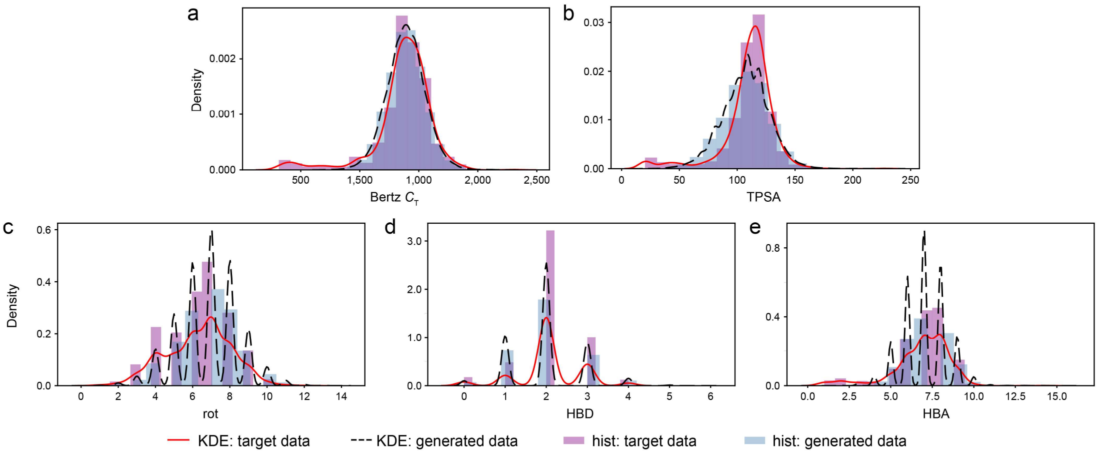

Supplementary Figure 3 | Comparison of five properties between the target data and the generated data. The histogram and kernel density plot of data distribution of Bertz C_T (**a**), the topological polar surface area (TPSA) (**b**), the number of rotatable bonds (rot) (**c**), the number of H-bond donors (HBD) (**d**), and the number of H-bond acceptors (HBA) (**e**). Source data are provided as a Source Data file.

a

Dataset	M	N	FNM	NSING	FNSING.M	FNSING.N	AUC	F50
The target data	1,000	48	0.05	18	0.02	0.38	0.91	0.04
The source data	1,000	180	0.18	111	0.11	0.62	0.87	0.03
The generated data	1,000	169	0.17	106	0.11	0.63	0.88	0.02
The filtered molecules	1,000	172	0.17	104	0.10	0.60	0.88	0.02

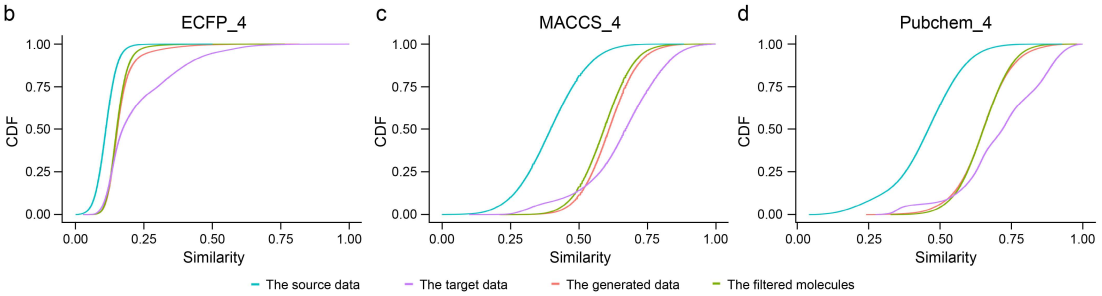

e

Fingerprint type: ECFP_4

Dataset	Min	1st Qu	Median	Mean	3rdQu	Max	Std.Dev
The target data	0.02	0.13	0.17	0.23	0.30	1.00	0.14
The source data	0.00	0.09	0.11	0.11	0.13	0.54	0.04
The generated data	0.03	0.13	0.15	0.16	0.18	0.83	0.06
The filtered molecules	0.03	0.13	0.15	0.16	0.17	0.98	0.04

f

Fingerprint type: MACCS_4

Dataset	Min	1st Qu	Median	Mean	3rdQu	Max	Std.Dev
The target data	0.10	0.58	0.67	0.66	0.76	1.00	0.15
The source data	0.00	0.32	0.40	0.40	0.48	0.91	0.12
The generated data	0.16	0.55	0.61	0.59	0.67	1.00	0.09
The filtered molecules	0.19	0.53	0.59	0.61	0.65	1.00	0.09

g

Fingerprint type: Pubchem_4

Pubchem_4	Min	1st Qu	Median	Mean	3rdQu	Max	Std.Dev
The target data	0.26	0.63	0.72	0.71	0.84	1.00	0.15
The source data	0.03	0.38	0.46	0.45	0.53	0.96	0.13
The generated data	0.22	0.59	0.65	0.65	0.71	1.00	0.10
The filtered molecules	0.28	0.60	0.65	0.65	0.71	1.00	0.09

Supplementary Figure 4 | Scaffold diversity and fingerprint diversity of the source data, the target data, the generated data and the filtered molecules with PUMA. a The summary statistics of scaffold diversity of the target data, the source data, the generative data, and the filtered molecules, corresponding to Figure 3d. **b-g** The fingerprint diversity analysis with Cumulative Distribution Function (CDF) curve of the pairwise Tanimoto similarity using ECFP_4 fingerprints (**b**), MACCS_4 fingerprints (**c**) and PubChem_4 fingerprints (**d**) of the target data, the source data, the generative data, and the filtered molecules. The corresponding summary statistics of fingerprint diversity using ECFP_4 fingerprints (**e**), MACCS_4 fingerprints (**f**) and PubChem_4 fingerprints (**g**) on the target data, the source data, the generative data, and the filtered molecules. Source data are provided as a Source Data file.

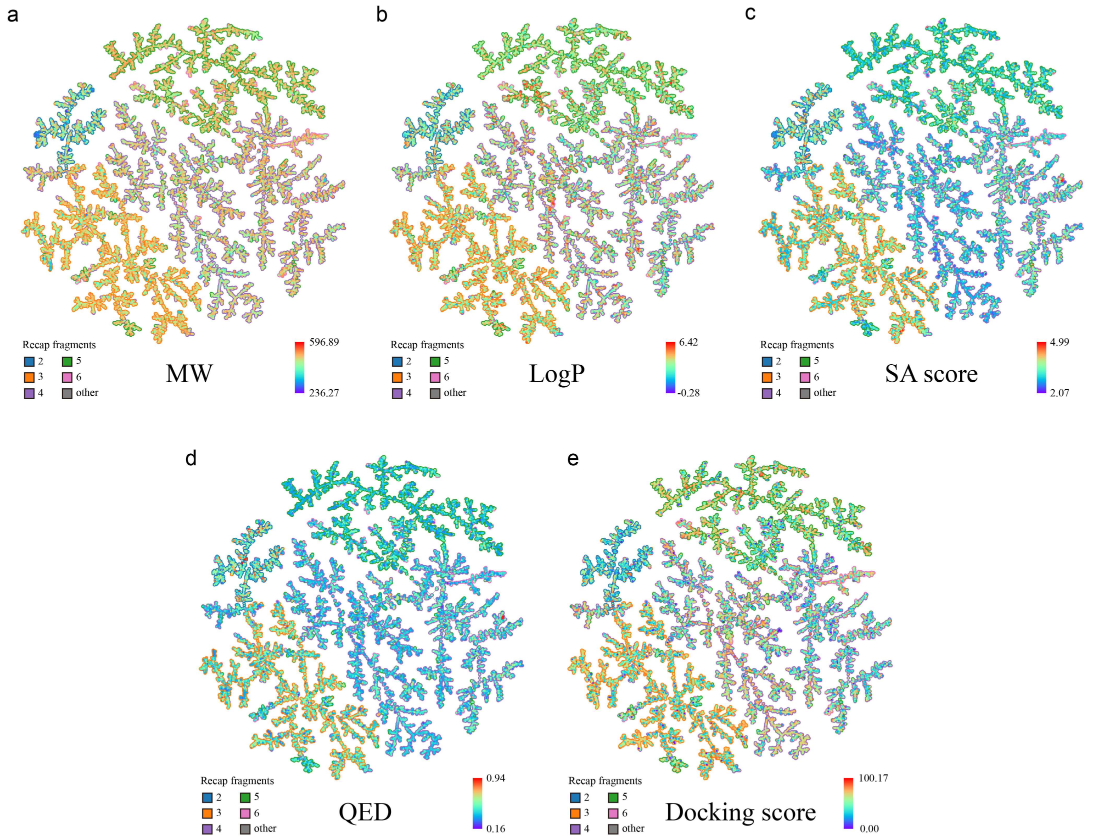

Supplementary Figure 5 | TMAPs of the filtered molecules in five color modes. The TMAPs are colored by the molecule properties (MW (**a**), LogP (**b**), SA score (**c**) and QED (**d**)) [red–yellow–green] or docking score (**e**) [red–yellow–green] for the first circle depicts, and the number of RECAP fragments for the second circle depicts.

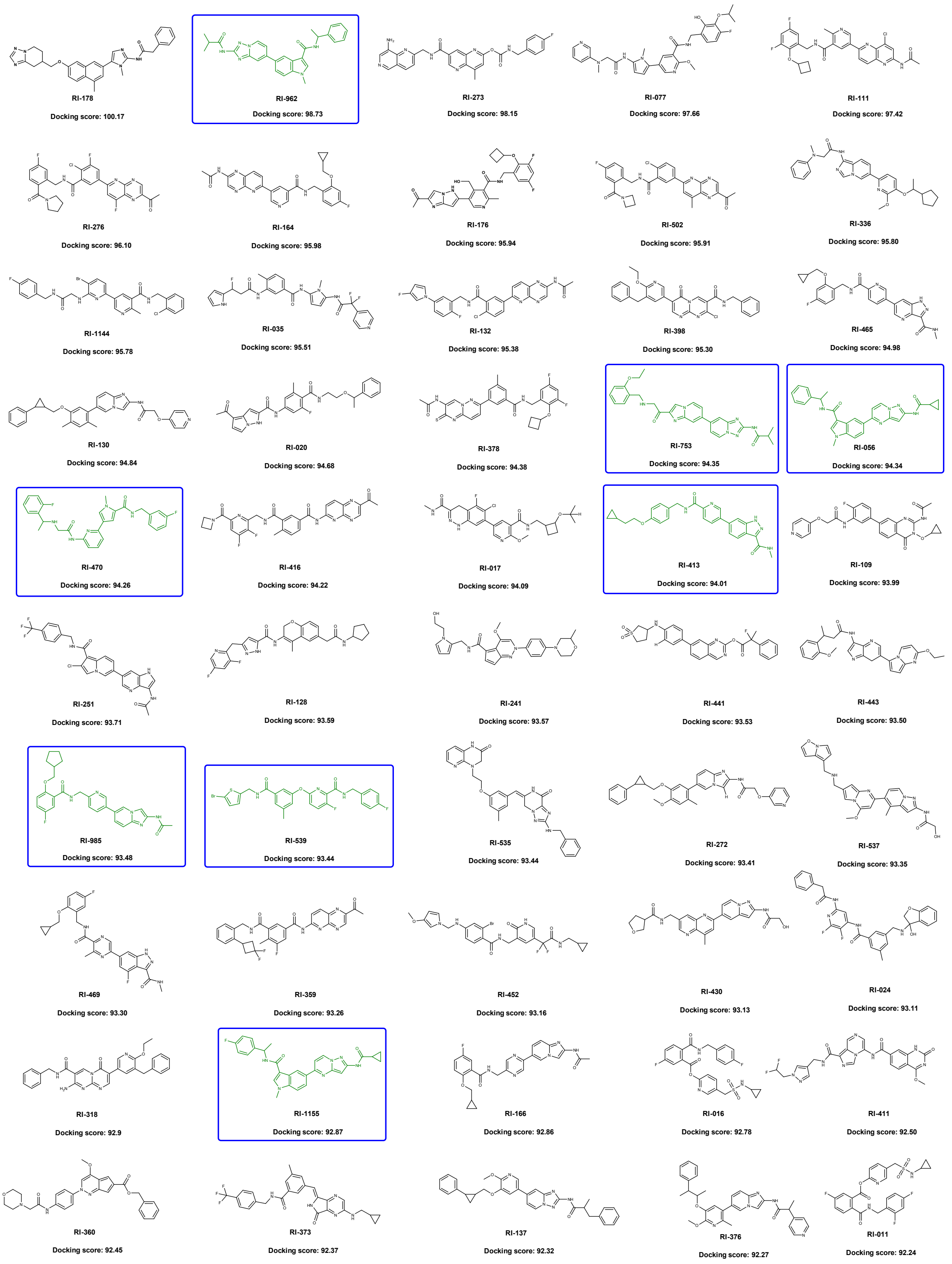

Supplementary Figure 6 | Chemical structures and docking scores of the top-ranked 50 molecules. Compounds selected for synthesis were colored in green and marked by blue box.

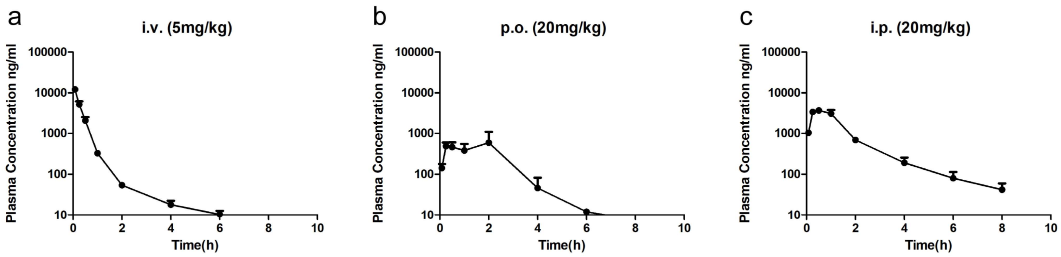

Supplementary Figure 7 | Pharmacokinetic parameters of RI-962 in Sprague-Dawley rats. Plot of plasma concentration versus time for intravenous (i.v.) administration (5 mg/kg) (**a**), oral (p.o.) administration (20 mg/kg) (**b**) and intraperitoneal (i.p.) administration (20 mg/kg) (**c**), respectively (n = 3). Data are presented as mean ± standard deviation (SD). Source data are provided as a Source Data file.

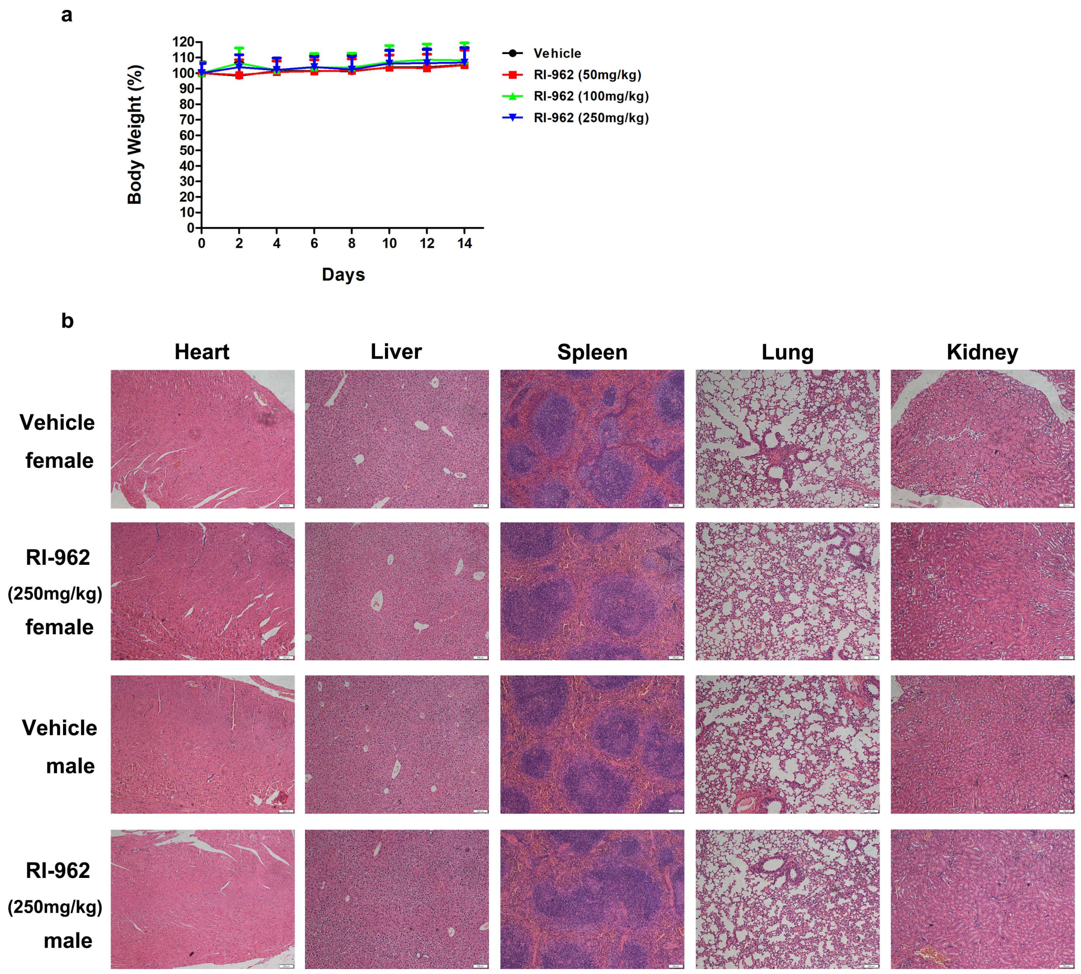

Supplementary Figure 8 | RI-962 did not show obvious adverse effects in mouse models. **a** Maximum tolerated dose study of RI-962 in C57B6 mice. Data are presented as the mean ± standard deviation (SD) of n = 6 independent mice per group. **b** RI-962 (250mg/kg) has no obvious impact on different mouse organs in indicated doses (H&E staining, scale bar = 100 μm). Each image is a representative one for those from at least three mice. Source data are provided as a Source Data file.

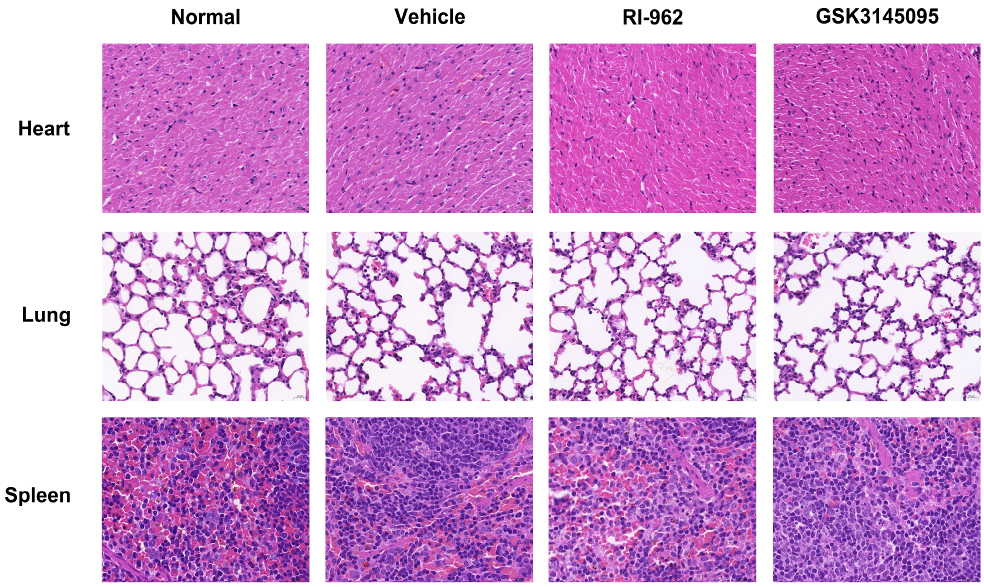

Supplementary Figure 9 | The mice (*n* = 4) were killed at 6 h after TNFα administration, and the heart, lung, spleen, and liver tissues were collected for analysis. Representative images of the histological analyses of the heart, lung, and spleen tissues by H&E staining in TNFα-induced SIRS model (scale bar = 20 μm). Data were obtained from two independent experiments.

Supplementary Tables

Supplementary Table 1 | Data source where we collected the known RIPK1 inhibitors (the target data) used in this paper.*

No	Data source	Type	Quantity
1	Ren, Y. et al. *J. Med. Chem.* (2017)	Article[1]	33
2	Karaman, M. W. et al. *Nat. Biotechnol.* (2008)	Article[2]	6
3	Davis, M. I. et al. *Nat. Biotechnol.* (2011)	Article[3]	10
4	Zarrinkar, P. P. et al. *Blood* (2009)	Article[4]	1
5	Harris, P. A. et al. *ACS Med. Chem. Lett.* (2013)	Article[5]	32
6	Harris, P. A. et al. *J. Med. Chem.* (2017)	Article[6]	28
7	Yoshikawa, M. et al. *J. Med. Chem.* (2018)	Article[7]	41
8	Harris, P. A. et al. *J. Med. Chem.* (2016)	Article[8]	12
9	Munoz, L. *Nat. Rev. Drug Discov.* (2017)	Article[9]	1
10	WO2020056072 (A1)	Patent	56
11	US20190337907 (A1)	Patent	92
12	US20190337934 (A1)	Patent	26
13	WO2019089442 (A1)	Patent	341
14	WO2018148626 (A1)	Patent	332
15	US 10457678 (B2)	Patent	10
16	US 10426758 (B2)	Patent	9
	**Total**		**1,030**

*Data collection was ended in July 2020.

Supplementary Table 2 | RIPK1 inhibitory activity and cell protection effect in TSZ-induced HT29 cell necroptosis model of selected compounds.

Compd.	Structure	RIPK1 IC_{50} (μM)	HT29 EC_{50} (μM)
**RI-056**	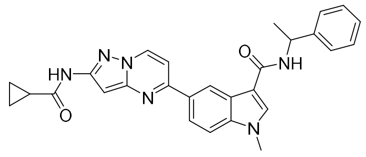	0.065	0.049
**RI-413**	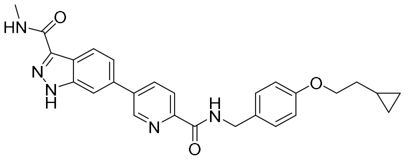	>10	>30
**RI-470**	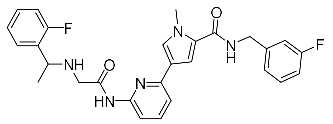	>10	>100
**RI-539**	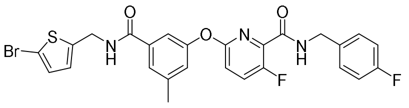	>10	>30
**RI-753**	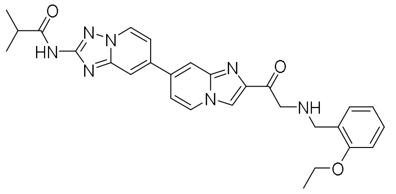	0.463	0.064
**RI-962**	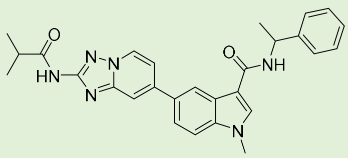	0.035	0.010
**RI-985**	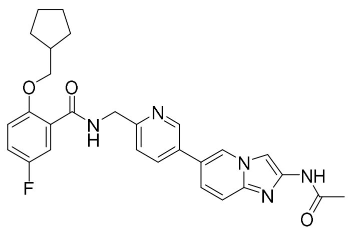	>10	>30
**RI-1155**	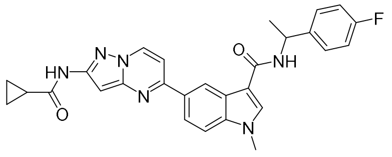	0.049	0.040

Supplementary Table 3 | Kinase inhibitory activities of compound RI-962@10 µM against 406 kinases.

Kinase	Activity (%)	Kinase	Activity (%)	Kinase	Activity (%)
AAK1(h)	106	B-Raf(h)	99	CDK5/p25(h)	92
Abl (H396P) (h)	110	B-Raf(V599E)(h)	107	CDK5/p35(h)	118
Abl (M351T)(h)	121	BRK(h)	101	CDK6/cyclinD3(h)	92
Abl (Q252H) (h)	135	BrSK1(h)	100	CDK7/cyclinH/MAT1(h)	93
Abl(h)	85	BrSK2(h)	110	CDK9/cyclin T1(h)	67
Abl(T315I)(h)	132	BTK(h)	92	CDKL1(h)	98
Abl(Y253F)(h)	102	BTK(R28H)(h)	105	CDKL2(h)	77
ACK1(h)	100	CaMKI(h)	92	CDKL3(h)	84
ACTR2(h)	81	CaMKIIα(h)	93	CDKL4(h)	70
ALK(h)	68	CaMKIIβ(h)	109	ChaK1(h)	85
ALK1(h)	94	CaMKIIγ(h)	93	CHK1(h)	124
ALK2(h)	104	CaMKIIδ(h)	92	CHK2(h)	98
ALK4(h)	90	CaMKIß(h)	100	CHK2(I157T)(h)	92
ALK6(h)	95	CaMKIV(h)	77	CHK2(R145W)(h)	99
AMPKα1(h)	93	CaMKIγ(h)	113	CK1(y)	100
AMPKα2(h)	89	CaMKIδ(h)	104	CK1γ1(h)	94
A-Raf(h)	80	CaMKK1(h)	56	CK1γ2(h)	88
Arg(h)	106	CaMKK2(h)	81	CK1γ3(h)	96
Arg(m)	90	Cdc7/cyclinB1(h)	53	CK1δ(h)	108
ARK5(h)	83	CDK1/cyclinB(h)	99	CK1ε(h)	83
ASK1(h)	97	CDK12/cyclinK(h)	117	CK2(h)	91
ATM(h)	85	CDK13/cyclinK(h)	100	CK2α1(h)	94
ATR/ATRIP(h)	108	CDK14/cyclinY(h)	91	CK2α2(h)	85
Aurora-A(h)	95	CDK16/cyclinY(h)	121	cKit(D816H)(h)	142
Aurora-B(h)	119	CDK17/cyclinY(h)	124	cKit(D816V)(h)	97
Aurora-C(h)	95	CDK18/cyclinY(h)	119	cKit(h)	105
Axl(h)	110	CDK2/cyclinA(h)	122	cKit(V560G)(h)	95

BIKe(h)	60	CDK2/cyclinE(h)	92	cKit(V654A)(h)	85
Blk(h)	96	CDK3/cyclinE(h)	119	CLIK1(h)	132
BMPR2(h)	105	CDK4/cyclinD3(h)	75	CLK1(h)	109
CLK2(h)	94	EphA4(h)	77	GRK2(h)	86
CLK3(h)	94	EphA5(h)	93	GRK3(h)	92
CLK4(h)	103	EphA7(h)	100	GRK5(h)	80
c-RAF(h)	114	EphA8(h)	88	GRK6(h)	108
CRIK(h)	101	EphB1(h)	111	GRK7(h)	60
CSK(h)	76	EphB2(h)	73	GSK3α(h)	112
cSRC(h)	100	EphB3(h)	77	GSK3β(h)	111
DAPK1(h)	95	EphB4(h)	90	Haspin(h)	107
DAPK2(h)	104	ErbB2(h)	104	Hck(h)	93
DCAMKL2(h)	132	ErbB4(h)	98	Hck(h) activated	97
DCAMKL3(h)	100	FAK(h)	108	HIPK1(h)	103
DDR1(h)	104	Fer(h)	107	HIPK2(h)	72
DDR2(h)	126	Fes(h)	99	HIPK3(h)	89
DMPK(h)	89	FGFR1(h)	81	HIPK4(h)	71
DNA-PK(h)	100	FGFR1(V561M)(h)	99	HPK1(h)	92
DRAK1(h)	100	FGFR2(h)	107	HRI(h)	99
DRAK2(h)	106	FGFR2(N549H)(h)	100	ICK(h)	102
DYRK1A(h)	96	FGFR3(h)	72	IGF-1R(h)	94
DYRK1B(h)	100	FGFR4(h)	85	IGF-1R(h), activated	92
DYRK2(h)	92	Fgr(h)	96	IKKα(h)	101
DYRK3(h)	83	Flt1(h)	85	IKKβ(h)	88
eEF-2K(h)	112	Flt3(D835Y)(h)	88	IKKε(h)	103
EGFR(h)	94	Flt3(h)	87	IR(h)	103
EGFR(L858R)(h)	93	Flt4(h)	86	IR(h), activated	89
EGFR(L861Q)(h)	82	Fms(h)	111	IRAK1(h)	103
EGFR(T790M)(h)	119	Fms(Y969C)(h)	73	IRAK4(h)	79
EGFR(T790M,L858R)(h)	85	Fyn(h)	107	IRE1(h)	115
EphA1(h)	97	GCK(h)	83	IRR(h)	100

EphA2(h)	96	GCN2(h)	76	Itk(h)	71
EphA3(h)	105	GRK1(h)	111	JAK1(h)	85
JAK2(h)	107	Met(D1246H)(h)	134	NEK1(h)	93
JAK3(h)	95	Met(D1246N)(h)	125	NEK11(h)	89
JNK1α1(h)	99	Met(h)	102	NEK2(h)	100
JNK2α2(h)	71	Met(M1268T)(h)	115	NEK3(h)	103
JNK3(h)	56	Met(Y1248C)(h)	116	NEK4(h)	104
KDR(h)	92	Met(Y1248D)(h)	110	NEK6(h)	99
Lck(h)	137	Met(Y1248H)(h)	99	NEK7(h)	85
Lck(h) activated	81	MINK(h)	63	NEK9(h)	85
LIMK1(h)	97	MKK3(h)	103	NIM1(h)	88
LIMK2(h)	101	MKK6(h)	97	NLK(h)	93
LKB1(h)	105	MLCK(h)	81	NUAK2(h)	86
LOK(h)	108	MLK1(h)	36	p70S6K(h)	106
LRRK2(h)	102	MLK2(h)	29	PAK1(h)	105
LTK(h)	100	MLK3(h)	20	PAK2(h)	84
Lyn(h)	65	Mnk2(h)	92	PAK3(h)	77
MAK(h)	126	MOK(h)	88	PAK4(h)	92
MAP4K3(h)	85	MRCKα(h)	94	PAK5(h)	93
MAP4K4(h)	88	MRCKβ(h)	79	PAK6(h)	87
MAP4K5(h)	100	MRCKγ(h)	83	PAR-1Bα(h)	103
MAPK1(h)	94	MSK1(h)	112	PASK(h)	71
MAPK2(h)	90	MSK2(h)	94	PDGFRα(D842V)(h)	104
MAPKAP-K2(h)	97	MSSK1(h)	85	PDGFRα(h)	99
MAPKAP-K3(h)	98	MST1(h)	95	PDGFRα(V561D)(h)	102
MARK1(h)	99	MST2(h)	64	PDGFRβ(h)	107
MARK3(h)	84	MST3(h)	149	PDHK2(h)	96
MARK4(h)	102	MST4(h)	119	PDHK4(h)	75
MEK1(h)	105	mTOR(h)	87	PDK1(h)	101
MEK2(h)	86	mTOR/FKBP12(h)	98	PEK(h)	94
MEKK2(h)	98	MuSK(h)	52	PhKγ1(h)	98
MEKK3(h)	93	MYLK2(h)	86	PhKγ2(h)	93

MELK(h)	95	MYO3B(h)	76	PI3(p110a(E542K)/p85a	97
Mer(h)	91	NDR2(h)	85	PI3(p110a(E545K)/p85a)	96
PI3 Kinase (p110a(H1047R)/ p85a)(h)	99	PKCι(h)	97	Rsk1(h)	120
PI3 Kinase (p110a/p65a)(h)	97	PKCμ(h)	87	Rsk2(h)	106
PI3 Kinase (p110a/p85a)(h)	94	PKD2(h)	98	Rsk3(h)	110
PI3 Kinase (p110b/p85a)(h)	88	PKD3(h)	104	Rsk4(h)	61
PI3 Kinase (p110d/p85a)(h)	64	PKG1α(h)	101	SAPK2a(h)	118
PI3 Kinase (p120g)(h)	80	PKG1β(h)	98	SAPK2a(T106M)(h)	86
PI3KC2a(h)	102	PKR(h)	97	SAPK2b(h)	84
PI3KC2g(h)	99	Plk1(h)	122	SAPK3(h)	100
Pim-1(h)	101	Plk3(h)	88	SAPK4(h)	91
Pim-2(h)	97	Plk4(h)	88	SBK1(h)	112
Pim-3(h)	111	PRAK(h)	64	SGK(h)	85
PIP4K2a(h)	98	PRK1(h)	109	SGK2(h)	120
PIP5K1a(h)	106	PRK2(h)	101	SGK3(h)	132
PIP5K1g(h)	98	PRKG2(h)	105	SIK(h)	137
PKA(h)	97	PrKX(h)	126	SIK2(h)	101
PKAcβ(h)	117	PRP4(h)	88	SIK3(h)	100
PKBα(h)	80	PTK5(h)	104	SLK(h)	84
PKBβ(h)	92	Pyk2(h)	106	Snk(h)	79
PKBγ(h)	95	Ret (V804L)(h)	103	SNRK(h)	121
PKCα(h)	97	Ret(h)	114	Src(1-530)(h)	95
PKCβI(h)	120	Ret(V804M)(h)	115	Src(T341M)(h)	100
PKCβII(h)	87	RIPK1(h)	0	SRMS(h)	101
PKCγ(h)	93	RIPK2(h)	79	SRPK1(h)	102
PKCδ(h)	90	ROCK-I(h)	99	SRPK2(h)	100
PKCε(h)	99	ROCK-II(h)	90	STK16(h)	100
PKCζ(h)	119	Ron(h)	104	STK25(h)	81

PKCη(h)	99	Ros(h)	106	STK32A(h)	113
PKCθ(h)	131	Rse(h)	108	STK32B(h)	85
STK32C(h)	76	TrkB(h)	126	WNK3(h)	75
STK33(h)	85	TrkC(h)	56	WNK4(h)	80
Syk(h)	55	TSSK1(h)	84	Yes(h)	97
TAF1L(h)	89	TSSK2(h)	76	ZAK(h)	72
TAK1(h)	90	TSSK3(h)	94	ZAP-70(h)	95
TAO1(h)	81	TSSK4(h)	104	ZIPK(h)	102
TAO2(h)	92	TTBK1(h)	92		
TAO3(h)	86	TTBK2(h)	81		
TBK1(h)	90	TTK(h)	92		
Tec(h) activated	103	Txk(h)	94		
TGFBR1(h)	98	TYK2(h)	113		
TGFBR2(h)	94	ULK1(h)	69		
Tie2 (h)	90	ULK2(h)	89		
Tie2(R849W)(h)	109	ULK3(h)	68		
Tie2(Y897S)(h)	69	VRK1(h)	86		
TLK1(h)	69	VRK2(h)	89		
TLK2(h)	78	Wee1(h)	92		
TNIK(h)	65	Wee1B(h)	95		
TRB2(h)	91	WNK1(h)	75		
TrkA(h)	64	WNK2(h)	79		

Supplementary Table 4 | IC_{50} values of compound RI-962 against selected kinases.

Kinase	IC_{50} (μM)
RIPK1	0.035
RIPK2	>10
RIPK3	>10
RIPK4	>10
MLK1	>10
MLK2	>10
MLK3	3.747

Supplementary Table 5 | Data collection and refinement statistics for X-ray structures.

	RIPK1–RI-962
PDB ID	7YDX
**Data collection**	
Space group	$P2_12_12_1$
Cell dimensions	
a, b, c (Å)	47.57, 97.53, 132.38
α, β, γ (°)	90.00, 90.00, 90.00
Resolution (Å)	50.00-2.64 (2.70-2.64)
R_{merge}	0.071 (0.925)
$I / \sigma I$	36.0 (2.2)
Completeness (%)	100.00 (100.00)
Redundancy	11.3 (11.5)
**Refinement**	
Resolution (Å)	48.77-2.64
No. reflections	18433 (1529)
R_{work} / R_{free}	0.260/0.289
No. atoms	
Protein	4249
Ligand/ion	79
Water	65
B-factors	
Protein	42.4
Ligand/ion	29.8
Water	42.0
R.m.s. deviations	
Bond lengths (Å)	0.011
Bond angles (°)	1.28

Supplementary Table 6 | Parameters used in the GDL model.

Parameter name	Candidate values	Default value
Batch size	64, 128, 256, 512	256
Batch size for transfer learning	5, 10, 20, 50	10
Feature extractor hidden unit number	32, 64, 128, 256	128
Feature extractor RNN layer number	1, 2, 4	1
Generative RNN hidden unit number	64, 128, 256, 512	256
Generative RNN layer number	1, 2, 4	1
Embedding size	16, 32, 64, 128	32
Gaussian distribution of noise (μ, σ^2)	(0.5, 0), (1, 0), (2, 0)	(1, 0)
Sampling number	10, 50, 100	50
Optimizer type	Adam, Gradient descent, Adagrad	Adam
Learning rate	0.00001, 0.0001, 0.001, 0.01	0.0001

Supplementary Notes

Supplementary Note 1 | Conversion between SMILES and word embedding matric.

All molecules were converted into canonized SMILES strings using RDkit to ensure the uniqueness of molecular representations. Molecules containing elements other than C, H, O, N, P, S, Cl, Br, F and I were removed.

The conversion from the SMILES strings to the inputs of model was achieved through the following two steps. In the first step, a pre-processed SMILES string was coded into a molecular vector V:

$$V = (v_1, v_2, \ldots, v_n), \tag{S1}$$

where v_n is the value of the nth token in the molecular vector V. We established the vocabulary of SMILES as the coding rules, which consists of the most frequent forms of the 10 elements, namely C, H, O, N, P, S, Cl, Br, F and I (Supplementary Note Table 1) instead of the single letter in a SMILES string. In each molecular vector, the placeholders <SOS>, <EOS> and <PAD> were introduced. The placeholder <SOS> with a value of 1 is the symbol of the start of the molecule, and <EOS> with a value of 2 is the symbol of the end of it. The placeholder <PAD> with a value of 0 was added after the <EOS> of a vector and used to pad the vector to a specific length. For example, suppose that the total length of the molecular vector is 16, the SMILES of benzene is "c1ccccc1", and its vector should be "1 24 29 24 24 24 24 24 29 2 0 0 0 0 0 0".

In the second step, the molecular vector was converted to a word embedding matric at the embedding layer by employing the word embedding algorithm[10,11]. The word embedding matric M is the input of model:

$$M = (x_1, x_2, \ldots, x_n), \tag{S2}$$

where x_i $(i = 1,2, \ldots, n)$ is a vector for the ith token in a word embedding matrix.

Suppose x_i has d dimensions, then the word embedding matric M is a 2D matrix of shape n-by-d. The reverse conversion from the outputs of model to the SMILES strings was performed in a similar way. We firstly converted an output word embedding matric to a molecular vector at the dense layer, and then coded the molecular vector back to the SMILES string using the same vocabulary of SMILES.

Supplementary Note Table 1 | Vocabulary of SMILES.

**<PAD>**	0	**<SOS>**	1	**<EOS>**	2	**one-char**	3-36
**two-char**	37-44	**three-char**	45-47	**bracket**	48-68	**branch**	69-104

one-char [3-36]
= # @ ( ) [ ] + - / \ C N O P S H F I c n o p s h 1 2 3 4 5 6 7 8 9
two-char [37-44]
@@ Cl Br =C =c =O =S =N
three-char [45-47]
C#N N#C C#C
Bracket [48-68]
[C@@H] [C@H] [NH+] [O-] [nH] [nH+] [N+] [NH2+] [C@] [C@@] [N-] [NH3+] [n+] [S-] [n-] [S@@] [S@] [NH-] [P@@] [P@] [H]
Branch [69-104]
(=O) (C) (F) (Cl) (O) (OC) (Br) ([O-]) (N) (CC) (C#N) (=S) (CO) (CCO) (\[O-]) (OCC) (I) ([S-]) (SC) (\C#N) (CCC#N) (CCC) (C=O) (=N) (CCOC) (CC#N) (/C#N) (CCCO) (CC=C) (\C) (COC) (\O) (/C) (CCCC) (CCl) ([H])

Supplementary Note 2 | Regularization enhancement.

Regularization through adding random noise to model during training can achieve significant improvements in generalization performance with a little computational overhead, which includes adding noise to model inputs, hidden layers and weights[18-26]. Among them, adding noise to model inputs has been reported to be effective in improving the generation performance in both supervised[18] and unsupervised networks[19,20].

Generally, the effect of adding noise to model inputs is considered to be equivalent to introducing penalty terms in the objective function[21-23]. Hence, the expected empirical cost with input noise can be written as:

$$E^{noisy} = E + \phi \tag{S3}$$

$$\phi = \eta^2 E^R, \tag{S4}$$

where ϕ is the penalty term, η^2 is the regularization term[21,24] which is controlled by the amplitude of the noise E^R.

Motivated by the success of adding noise to the model inputs, this paper used the random noise-added input during training to improve the generalization performance of generative RNN. The original model trained the mapping $p(X_{\text{out}}|X_{\text{in}})$ between the input X_{in} and the output X_{out} (Eq. (S5), (S6)). While during training with regularization enhancement, we learn a stochastic operator $p(\tilde{X}_{\text{out}}|\tilde{X}_{\text{in}})$ that maps a regularized output $\tilde{X}_{\text{out}}$ back to its corresponding unregularized output X_{out} (Eq. (S7), (S8)).

$$X_{\text{in}} = (x, h_0) \tag{S5}$$

$$X_{\text{out}} = f(x, h_0) \to x \tag{S6}$$

$$\tilde{X}_{\text{in}} = (x, \tilde{h}_0) \tag{S7}$$

$$\tilde{X}_{\text{out}} = f(x, \tilde{h}_0) \to x. \tag{S8}$$

Also, an intuitive geometric interpretation can be given to this regularization enhancement process under the manifold assumption[19,25], which states that natural high-dimensional data concentrate close to a non-linear low-dimensional manifold (Supplementary Note Figure 1). Suppose training data x (black cross) concentrate near a low-dimensional manifold, and so do the unregularized outputs X_{out} of inputs X_{in}. In contrast, the regularized output $\tilde{X}_{\text{out}}$ (red solid circle) of input data $\tilde{X}_{\text{in}} = (x, \tilde{h}_0)$ (the random noise-added input obtained by applying regularization process $q_D(\tilde{h}_0|h_0)$) will generally lie farther from the manifold. The model learns $p(X_{\text{out}}|\tilde{X}_{\text{out}})$ to project the regularized output $\tilde{X}_{\text{out}}$ (red solid circle) back (via generative RNN $f(\tilde{X}_{\text{in}})$) onto the manifold. Intermediate representation $\tilde{X}_{\text{out}} = f(\tilde{X}_{\text{in}})$ may be interpreted as a coordinate system for points x on the manifold.

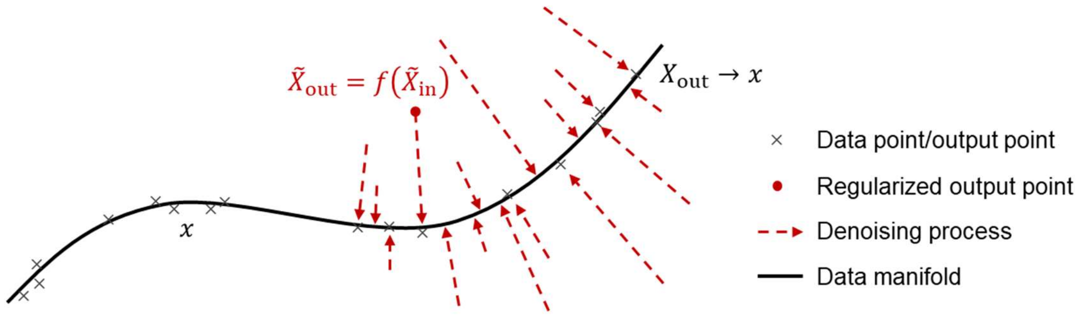

Supplementary Note Figure 1 | Manifold assumption.

Supplementary Note 3 | Sampling enhancement.

The generative network can generate previously unseen data, which demonstrates that the network internally learns the molecular representation. We explore three methods that generate molecules by sampling from the latent space, including single-point sampling (Sample), linear interpolation (Linear) and spherical interpolation (Slerp)[27-30].

Single-point sampling[27]. In order to generate novel molecules different from those in training dataset, we introduced diversity in generation process by adding a multidimensional Gaussian parameter ξ_s to the original hidden vector h_0^i (Eq. (9)). ξ_s was randomly sampled from a prior Gaussian distribution with zero-mean and defined variance. And then, the new hidden vectors $h_{0,\text{new}}$ were created according to the distribution centered upon the original hidden vectors h_0 but with a larger variance. This allowed us to explore the chemical space around an origin molecule, with a tunable amount of diversity, corresponding to the variability in chemical space.

Interpolation is used to traverse between two known locations in latent space, and has been commonly employed in generative models.

Linear interpolation[28]. Linear interpolation is a frequently used method. Its implementation process is described as follows[29]. We first randomly sampled a pair of

hidden vectors h_0^i and h_0^j. Then, we obtained a series of latent vectors $h_{0,\text{new}}^{ij,\alpha}$ with linear interpolation factor α ranging from 0 to 1 (Eq. (7)). The geometric interpretation of linear interpolation process was provided. Suppose point p moves from p_1 to p_2 (Supplementary Note Figure 2a). For any p, there is,

$$p = (l - m)p_1 + mp_2. \tag{S9}$$

Set $t = \frac{m}{l}, t\epsilon(0,1)$, then,

$$p = (1 - t)p_1 + tp_2. \tag{S10}$$

Thus, linear interpolation in a high-dimension representation-space would yield new intermediate vectors following the shortest Euclidean path between their latent representations. Also, it only introduces minimal computation overhead.

Spherical interpolation[30]. Other than linear interpolation, another well-known interpolation method is the spherical interpolation method named slerp[28,30]. Given hidden vectors h_0^i and h_0^j, the latent vectors $h_{0,\text{new}}^{ij,\beta}$ were then obtained with spherical interpolation factor β, following the formula introduced by Shoemake et al.[31] (Eq. (8)). As a key parameter, the central angle θ can be calculated by the following formula,

$$\theta = \arccos\left(h_0^i \cdot h_0^j\right). \tag{S11}$$

Further, for any vector $\mathbf{x}$, there is,

$$\mathbf{x} = \frac{x}{\|x\|_2} = \frac{x}{\sqrt{\sum_{n=1}^{\mathrm{N}} x_n^2}}. \tag{S12}$$

Also, the geometric interpretation of spherical interpolation process was described as follows. Suppose point p moves from point p_1 to point p_2 along the arc (Supplementary Note Figure 2b). For any vector $\mathbf{p}$, there is,

$$\mathbf{p} = \mathbf{p}_1' + \mathbf{p}_2'. \tag{S13}$$

$\perp\mathbf{p}_1$ is the vertical vector of $\mathbf{p}_1'$, then,

$$\mathbf{p}_2' = \frac{\sin}{\sin\theta}\mathbf{p}_2. \tag{S14}$$

And $\mathbf{p}_1'$ can be inferred as follows,

$$\mathbf{p}_1' = \frac{\sin(\theta-\Omega)}{\sin\theta}\mathbf{p}_1. \tag{S15}$$

Set $t = \frac{\Omega}{\theta}, t \in (0,1)$, then,

$$\mathbf{p} = \frac{\sin[(1-t)\theta]}{\sin\theta}\mathbf{p}_1 + \frac{\sin(t\theta)}{\sin\theta}\mathbf{p}_2. \tag{S16}$$

Unlike linear interpolation, spherical interpolation follows a circular arc lying on the surface of an N-dimensional sphere, and is capable to sample the apace with high probability[28,30].

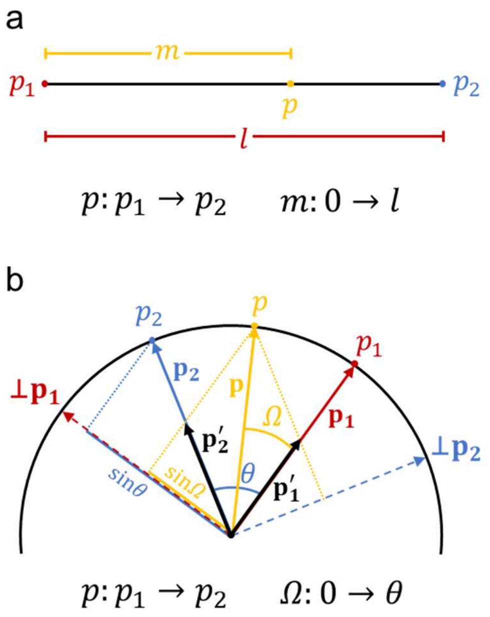

Supplementary Note Figure 2 | Diagram of linear interpolation (a) and spherical interpolation (b).

Supplementary Note 4 | Full-feature pharmacophore map.

As one of the major tools of computer-aided drug design (CADD), pharmacophore model methods including ligand-based and structure-based methods, have been widely developed and successfully applied in *de novo* drug design. The ligand-based

pharmacophore method overly depends on the selection of training set. The classic structure-based pharmacophore method, which is based on *apo* structures or a single protein–ligand complex, lacks statistically significant information. The multicomplex-based full-feature pharmacophore map that is created with multiple protein–ligand complexes is a competitive choice[32,33]. It allows for detecting all the interaction patterns and evaluating the importance of each protein–ligand interaction. In this study, we have utilized a collection of 13 crystal structures of human RIPK1 bound to a variety of inhibitors to generate a full-feature pharmacophore map by using multicomplex-based map and most-frequent-feature method.

Protein and ligand preparation. To create the structure-based pharmacophore model for RIPK1 ATP-competitive inhibitors, only *holo* structures of human RIPK1 with inhibitors targeting against the ATP binding pocket were collected. The *holo* structures with ATP, ATP analogues and natural products were excluded to avoid introducing unnecessary noise. In total, 13 crystallography structures of RIPK1 were obtained from the protein data bank (PDB)[34] (Supplementary Note Table 2). Waters, ions and other solvent molecules were removed due to their limited positional conservation[35]. Structures were verified by visual inspection. As a necessary step for further analysis, structural alignment based on sequence similarity and root mean squared deviation (RMSD) was performed using Modeller[36] in Discovery Studio 3.1. The crystal structure with PDB code "4NEU" was chosen as the reference structure.

Supplementary Note Table 2 | List of RIPK1 complexes.

No.	PDB ID	Resolution(Å)	Ligand	Release date
1	4ITJ	1.80	1HX	2013-Mar-13
2	4ITI	2.86	1HW	2013-Mar-13
3	4ITH	2.25	RCM	2013-Mar-13
4	4NEU	2.57	Q1A	2013-Nov-20

5	5HX6	2.23	65U	2016-Mar-02
6	5TX5	2.56	7MJ	2017-Jul-05
7	6C4D	2.52	EJP	2018-Mar-21
8	6HHO	3.49	G4W	2018-Dec-12
9	6NW2	2.00	L4Y	2019-May-01
10	6R5F	3.25	JSW	2019-May-01
11	6OCQ	2.79	M5J	2019-May-08
12	6NYH	2.10	L8D	2019-May-29
13	6RLN	2.87	K8K	2019-Jul-03

Full-feature pharmacophore map. The module "Recepter–Ligand Pharmacophore Generation" LigandScout[37], which could automatically construct 3D pharmacophore from the structural data of protein–ligand complex, was used to generate 13 individual complex-based pharmacophore models based on the previously aligned structures. For the purpose of creating full-feature pharmacophore map, all the identified pharmacophore features were then clustered according to their interaction pattern with the receptor. Hydrogen bond donor (HBD), hydrogen bond acceptor (HBA) and excluded volume features were clustered by their corresponding residue atoms. Hydrophobic, positive/negative ionizable and ring aromatic features were clustered by their density centers. The primary pharmacophore model was further modified according to the constraint tolerance of feature spheres using Catalyst[38] with default values. Due to its enormous computational cost, the full-feature pharmacophore map obtained initially is not suitable for virtual screening. Thus, a simplified model with selected pharmacophore features representing important protein–ligand interactions was constructed for practical application. As a viable solution, top-ranked features using frequency-based method were chosen and merged to create the most-frequent-feature pharmacophore map that consisted of top-ranked 6 features (A1, D1, H1, H2, H3, H4, H5 and H6) with frequencies greater than 30% in the 13 complexes

(Supplementary Note Table 3, Supplementary Note Figure 3a). Because of their critical contribution to the activity[5,39], 5 features named A2 (Met95: N), D1 (Glu63: OE2), D2 (Met95: O), D3 (Ser161: OG), and H6 (Ile43, Tyr94, Leu145), were added to the pharmacophore model as a necessary complement. The refined multicomplex-based comprehensive pharmacophore map consisted of 11 clustered features, including 2 hydrogen bond acceptors (A1–A2), 3 hydrogen bond donors (D1–D3) and 6 hydrophobic features (H1–H6) (Supplementary Note Figure 3b). In the previous study, Rajanikant et al.[40,41] have reported the ensemble pharmacophore model for RIPK1 inhibitors based on crystal structures 4ITH, 4ITI and 4ITJ. Compared with that, our model was created based on 13 *holo* structures of RIPK1 (including 10 structures from the latest reports) with various interaction modes, and used the frequency-based method to distinguish the key features from others.

Further, for a full pharmacophore map, it is also important to include excluded volume features, which reflect potential steric restriction and correspond to the positions that are inaccessible to any potential ligand. In order to reduce the influence of flexible side-chain residues Lys45 and Met67, we removed the corresponding excluded volume features with distance less than 2 Å from hydrophobic center. 25 excluded volume features were found in the ATP-binding site, which correspond to the spaces occupied by residues Val31, Ile43, Met44, Lys45, Met67, Asn68, Leu70, Val75, Val76, Leu78, Lys77, Leu90, Val91, Met92, Leu129, Val134, Ile135, His136, Ile154, Ala155, Asp156, Leu157, Leu159, Ser161 and Phe162. Full-feature pharmacophore map involving excluded volume spheres is shown in Supplementary Note Figure 3c. In-house automatic pipeline was developed for acceleration. All the processes above were performed in Discovery Studio 3.1.

Supplementary Note Table 3 | Spreading of full-feature pharmacophore map features.

No.	Feature name	ID	Count	Frequency (%)	Interaction
1	HBA-F 1	A1	15	100	Asp156: N
2	Hydrophobic 1	H1	8	62	Ile154, Asp156
3	Hydrophobic 2	H2	8	62	Val75, Leu78, Phe162
4	Hydrophobic 3	H3	6	46	Lys45, Leu157
5	Hydrophobic 4	H4	6	46	Ile43, Lys45, Met92
6	Hydrophobic 5	H5	5	38	Met92, Leu157

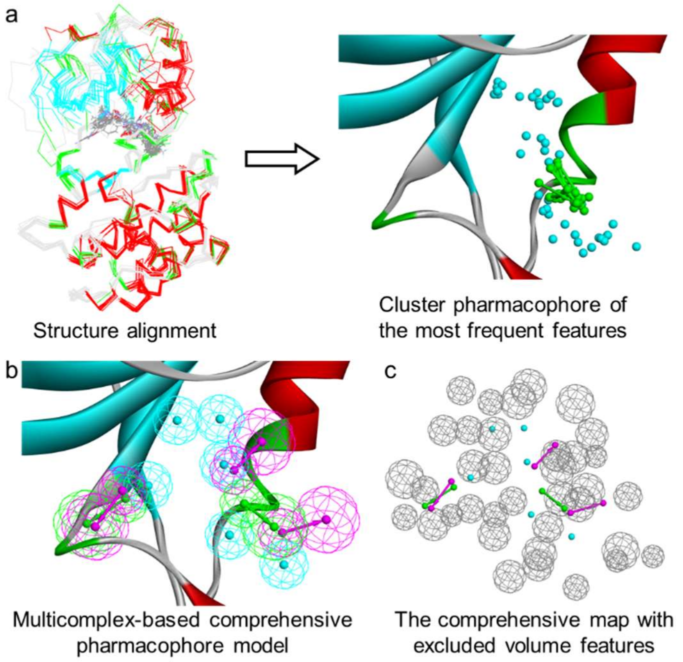

Supplementary Note Figure 3 | Generation of multicomplex-based comprehensive pharmacophore model. a Flowchart of the most-frequent-feature pharmacophore model generation. **b** All the clustered pharmacophore features of the multicomplex-based full-feature pharmacophore map. **c** The full-feature pharmacophore map with excluded volume features.

Supplementary Methods

General Remarks and Instrumentation

All chemical solvents and reagents were purchased from commercial suppliers and were used without further purification. All reactions were monitored by thin layer chromatography (TLC) and visualization was achieved by using ultraviolet light (254 nm) or display by iodine reagent. Column chromatography was carried out using Biotage Isolera flash purification system under proper pressure. ^{1}H NMR and ^{13}C NMR spectra were recorded on a Bruker Avance 400 spectrometer (Bruker Company, Germany) using TMS as an internal standard. Chemical shifts were given in ppm (parts per million). Low resolution ESI-MS readings were recorded on an Agilent 1200-G6410A mass spectrometer. High-resolution mass spectra were recorded on Q-TOF Premier mass spectrometer (Micromass, Manchester, UK). The purity was determined on a Waters e2695 series LC system (phenomenex-C18 reversed-column (4.6 mm × 150 mm, 5 μm), methanol (60%)/H_2O (40%); low rate, 1.0 mL/min; UV wavelength, 254-400 nm. The purity of compounds was determined to be over 95% by reversed-phase HPLC analysis.

Procedure for the Synthesis of RI-470

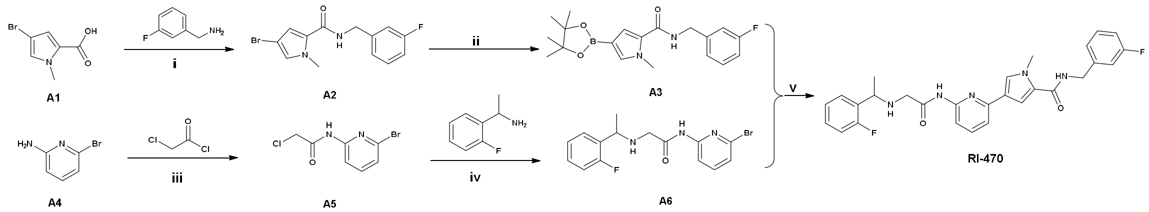

Reagents and conditions: (i) T_3P, THF, 45 °C, 4 h, 82%; (ii) diboron pinacol ester, Pd(dppf)Cl_2, KOAc, 1,4-dioxane, 95 °C, 3 h, 65%; (iii) K_2CO_3, THF, 0°C-rt, 12h, 85%;

(iv) DIEA, Acetonitrile, 80 °C, 4 h; (v) $PdCl_2(dppf)\cdot CH_2Cl_2$ adduct, Cs_2CO_3, 1,4-dioxane/water (v/v, 10/1), 100 °C, 12 h, 50%;

*4-bromo-N-(3-fluorobenzyl)-1-methyl-1H-pyrrole-2-carboxamide **(A2)*** A THF solution (30 mL) containing 4-bromo-1-methyl-1*H*-pyrrole-2-carboxylic acid (**A1**, 0.50g, 2.45 mmol, Bide Pharmatech Ltd.), (3-fluorophenyl)methanamine (0.31g, 2.45mmol, Bide Pharmatech Ltd.), 4-dimethylaminopyridine(0.60g, 4.90 mmol, Macklin Biochemical Co., Ltd) and T_3P (50 wt % in Ethyl acetate, 1.82 mL, Accela ChemBio Co., Ltd.) was stirred at 45 °C for 4 h. The reaction mixture was removed under reduced pressure and the residue was purified through silica gel chromatography (petroleum ether/ethyl acetate = 3:1) to afford the title compound **A2** as a white solid. Yield 82%. ^{1}H NMR (400 MHz, DMSO-d_6) δ 8.87-.76 (m, 1H), 7.35 (td, J = 7.5, 5.0 Hz, 1H), 7.24-7.17 (m, 1H), 7.09 (dp, J = 8.0, 1.3 Hz, 1H), 7.05-6.96 (m, 2H), 6.92 (d, J = 1.5 Hz, 1H), 4.41 (dt, J = 8.5, 0.9 Hz, 2H), 3.84 (s, 3H). MS (ESI, positive ion) m/z: 312.0 $[M + H]^+$.

*N-(3-fluorobenzyl)-1-methyl-4-(4,4,5,5-tetramethyl-1,3,2-dioxaborolan-2-yl)-1H-pyrrole-2-carboxamide **(A3)*** A flask charged with **A2** (0.3g, 0.96mmol), diboron pinacol ester (0.73g, 2.88mmol, Accela ChemBio Co., Ltd.), KOAc(0.19g, 1.92mmol, Accela ChemBio Co., Ltd.) and $Pd(dppf)Cl_2$ (70 mg, 0.096 mmol, Energy Chemical) was flushed with nitrogen. Anhydrous 1,4-dioxane (15 mL, Energy Chemical) was then added. After being stirred at 95 °C for 3 h, solvent was filtrated and the filtrate was concentrated in vacuo, then the residue was purified through silica gel chromatography (petroleum ether/ethyl acetate = 3:1) to afford the title compound as a white solid. Yield 65%. ^{1}H NMR (400 MHz, DMSO-d_6) δ 8.73 (t, J = 8.5 Hz, 1H), 7.33 (td, J = 7.4, 4.9 Hz, 1H), 7.25 (d, J = 1.3 Hz, 1H), 7.21-7.17 (m, 1H), 7.12-7.07 (m, 2H), 7.03-6.97 (m, 1H), 4.40 (dt, J = 8.6, 1.0 Hz, 2H), 3.85 (s, 3H), 1.24 (s, 12H). MS (ESI, positive ion) m/z: 359.2 $[M + H]^+$.

*N-(6-bromopyridin-2-yl)-2-chloroacetamide **(A5)*** To a solution of 6-bromopyridin-2-amine (**A4,** 1.00 g, 5.78 mmol) and potassium carbonate (2.40 g, 17.34 mmol, Energy Chemical) in THF (35 mL, Energy Chemical), chloroacetyl chloride (0.66 g, 5.78 mmol,

Energy Chemical) was added dropwise at rt, and the resulting mixture was stirred at room temperature for 3 h. The reaction mixture was evaporated under reduced pressure, and the solid residue was triturated with a saturated aqueous solution of $NaHCO_3$ and filtered. The solid was washed successively with water and hexane in 85% yield. ^{1}H NMR (400 MHz, DMSO-d_6) δ 7.86 (d, J = 7.5, 1H), 7.66 (t, J = 7.5 Hz, 1H), 7.31 (d, J = 7.4 Hz, 1H), 4.25 (s, 2H). MS (ESI, positive ion) m/z: 250.3 $[M + H]^+$.

N-(6-bromopyridin-2-yl)-2-((1-(2-fluorophenyl)ethyl)amino)acetamide **(A6)** To a suspension of 800 mg (3.19 mmol) of *N*-(6-bromopyridin-2-yl)-2-chloroacetamide **A5** in 20 mL of acetonitrile (Bide Pharmatech Ltd.), 440 mg (3.19 mmol) of 1-(2-fluorophenyl)ethan-1-amine (Energy Chemical)and 1.59 mL DIEA (Energy Chemical) were added. The resulting mixture was stirred at 80 °C for 4 hours and then cooled to room temperature. The organic solution was concentrated in vacuo, and the residue was purified through chromatography (petroleum ether / ethyl acetate = 2:1) to afford the title compound in 60% yield as a white solid. ^{1}H NMR (400 MHz, DMSO-d_6) δ 7.88 (d, J = 7.3 Hz, 1H), 7.67 (t, J = 7.5 Hz, 1H), 7.46-7.35 (m, 1H), 7.35-7.25 (m, 3H), 7.17-7.06 (m, 1H), 4.74 (d, J = 6.8, 1H), 3.80 (td, J = 8.4, 7.7 Hz, 1H), 3.46 (d, J = 8.5 Hz, 1H), 3.33 (d, J = 8.4 Hz, 1H), 1.53 (d, J = 6.9 Hz, 3H). MS (ESI, positive ion) m/z: 353.0 $[M + H]^+$.

N-(3-fluorobenzyl)-4-(6-(2-((1-(2-fluorophenyl)ethyl)amino)acetamido)pyridin-2-yl)-1-methyl-1H-pyrrole-2-carboxamide (RI-470) Intermediate **A3** (300 mg, 0.84 mmol), intermediate **A6** (300 mg, 0.84 mmol), $PdCl_2(dppf)\cdot CH_2Cl_2$ adduct (69 mg, 0.084 mmol, Bide Pharmatech Ltd.) and Cs_2CO_3 (680 mg, 2.10 mmol, Energy Chemical) were dissolved in 1,4-dioxane and water (v/v, 10/1, Energy Chemical) at room temperature and heated at 100 °C for 12 h under nitrogen protection. After the reaction was complete as indicated by TLC, the reaction mixture was filtered through a Celite bed, and the filtrate was concentrated and purified by silica gel chromatography (petroleum ether/ethyl acetate = 1:6) to afford the title compound as a yellow solid. After washing by Et_2O, the desired product was obtained.Yield 50%. ^{1}H NMR (400 MHz, DMSO-d_6) δ 9.92 (s, 1H), 8.90 (d, J = 5.8 Hz, 1H), 7.86-7.70 (m, 2H), 7.57 (d, J = 6.5 Hz, 2H),

7.45 (d, J = 2.0 Hz, 1H), 7.41 – 7.33 (m, 1H), 7.28 (dd, J = 9.1, 6.7 Hz, 2H), 7.15 (d, J = 8.1 Hz, 5H), 4.42 (d, J = 6.2 Hz, 2H), 4.10 (d, J = 6.7 Hz, 1H), 3.90 (s, 3H), 3.27 – 3.13 (m, 2H), 1.36 (d, J = 6.6 Hz, 3H). ^{13}C NMR (101 MHz, DMSO-d_6) δ 171.12, 161.62, 159.40, 152.51, 151.07, 143.62, 139.26, 130.64, 129.01, 128.28, 127.49, 126.87, 125.06, 123.68, 122.57, 115.80, 115.58, 114.41, 114.20, 113.97, 113.76, 111.10, 110.14, 51.14, 50.81, 41.91, 37.06, 23.22. HRMS (m/z): calculated for $C_{28}H_{27}F_2N_5O_2^+$ [M+H]$^+$ 504.22660; found, 504.22060. HPLC purity: 97.06%, t_R = 6.10 min.

Procedure for the Synthesis of RI-413

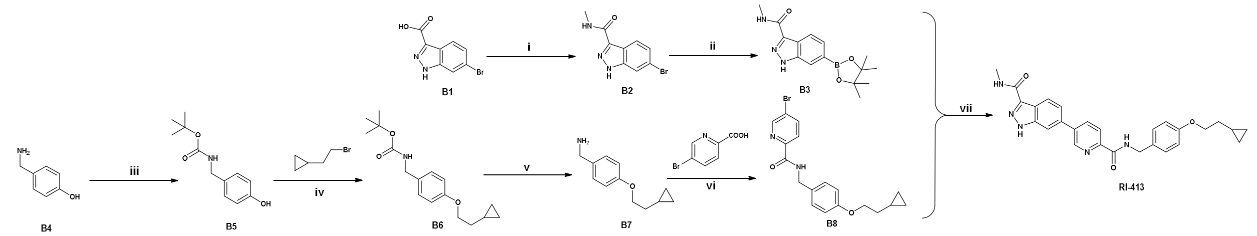

Reagents and conditions: (i) Methylamine hydrochloride, HOBt, EDCI, DIEA, DMF, rt, 3 h, 63%; (ii) diboron pinacol ester, Pd(dppf)Cl_2, KOAc, 1,4-dioxane, 95 °C, 4 h, 45%; (iii) Di-tert-butyl dicarbonate, DIEA, acetonitrile, 70 °C, 2 h, 90%; (iv) Potassium tert-butoxide, DMF, 90 °C, 4 h, 60%; (v) HCl 1,4-dioxane solution, 2 h, 65 °C; (vi) T_3P, THF, 45 °C, 2 h, 58%; (vii) $PdCl_2$(dppf)·CH_2Cl_2 adduct, tricyclohexylphosphane, Cs_2CO_3, 1,4-dioxane/water (v/v, 10/1), 100°C, 12 h, 48%;

6-bromo-N-methyl-1H-indazole-3-carboxamide ***(B2)*** A DMF solution (50 mL) containing 6-bromo-1*H*-indazole-3-carboxylic acid (**B1**, 2 g, 8.30 mmol, Macklin Biochemical Co., Ltd), HOBt (1.46 g, 10.79 mmol, Energy Chemical) and EDCI (3.18 g, 16.60 mmol, Energy Chemical) was stirred at room temperature for 30 min. DIEA (2.75 mL, 16.60 mmol, Energy Chemical) and methylamine hydrochloride (0.63 g, 9.13 mmol, Energy Chemical) were then added to the reaction mixture. After 3 h solvent was removed under reduced pressure, and the residue was partitioned between DCM and saturated sodium carbonate solution. The organic layer was concentrated under reduced pressure and the residue was purified through silica gel chromatography (petroleum ether/ethyl acetate = 3:1) to afford the title compound as a white solid. Yield

63%. ^{1}H NMR (400 MHz, DMSO-d_6) δ 11.94 (s, 1H), 8.05 (d, J = 1.5 Hz, 1H), 7.98 (d, J = 7.5 Hz, 1H), 7.71 (dd, J = 7.5, 1.5 Hz, 1H), 7.58 (t, J = 4.8 Hz, 1H), 2.89 (d, J = 4.8 Hz, 3H). MS (ESI, positive ion) m/z: 254.9 $[M + H]^+$.

*N-methyl-6-(4,4,5,5-tetramethyl-1,3,2-dioxaborolan-2-yl)-1H-indazole-3-carboxamide **(B3)*** A flask charged with **B2** (1.0 g, 3.94 mmol), diboron pinacol ester (2.0 g, 7.88mmol, Accela ChemBio Co., Ltd.), KOAc (0.77 g, 7.88 mmol, Accela ChemBio Co., Ltd.) and Pd(dppf)Cl_2 (290 mg, 0.39 mmol, Bide Pharmatech Ltd.) was flushed with nitrogen. Anhydrous 1,4-dioxane (50 mL, Energy Chemical) was then added. After being stirred at 95 °C for 4 h, solvent was filtrated and the filtrate was concentrated in vacuo, then the residue was purified through silica gel chromatography (petroleum ether/ethyl acetate = 1:3) to afford the title compound as a white solid. Yield 45%. ^{1}H NMR (420 MHz, DMSO-d_6) *δ* 11.92 (s, 1H), 7.97 (d, J = 1.1 Hz, 2H), 7.72 (t, J = 1.0 Hz, 1H), 7.52 (t, J = 4.8 Hz, 1H), 2.94 (d, J = 4.7 Hz, 3H), 1.23 (s, 12H). MS (ESI, positive ion) m/z: 302.1 $[M + H]^+$.

*Tert-butyl (4-hydroxybenzyl)carbamate **(B5)*** To a solution of **B4** (6.40 g, 25.3 mmol) in acetonitrile (100 mL, Energy Chemical) were added DIEA (3.9 g, 30.36 mmol, Energy Chemical) and di-tert-butyl dicarbonate (6.6 g, 30.36 mmol, Bide Pharmatech Ltd.), and the resultant mixture was stirred at 70 °C for 2 h. After the time quantum, the reaction mixture was concentrated in vacuo and the residue was redissolved in brine (100 mL). The resulting white precipitate was filtered and washed with ethanol to give the title compound in 90% yield as a white solid. ^{1}H NMR (400 MHz, DMSO-d_6) δ 8.65 (s, 1H), 7.21 (d, J = 7.6 Hz, 2H), 7.01 (t, J = 8.7 Hz, 1H), 6.96-6.91 (m, 2H), 4.32 (d, J = 8.7 Hz, 2H), 1.41 (s, 9H). MS (ESI, positive ion) m/z: 224.1 $[M + H]^+$.

*Tert-butyl (4-(2-cyclopropylethoxy)benzyl)carbamate **(B6)*** To a stirred mixture of **B5** (0.30 g, 1.34 mmol) and potassium tert-butanolate (0.30 g, 2.68 mmol, Bide Pharmatech Ltd.) were added DMF (20 mL, Energy Chemical) and (2-bromoethyl)cyclopropane (0.17 mL, 1.61 mmol, Accela ChemBio Co., Ltd.) in one portion. The solution was warmed to 90 °C for 4 h. The solvent was concentrated in vacuo and the residue was partitioned between DCM (20 mL) and saturated aqueous sodium bicarbonate solution

(20 mL). The organic layer was dried over MgSO4, filtered, and concentrated in vacuo to give the yellow oil that was purified by silica gel chromatography (ethyl acetate / petroleum ether = 1:5) to afford **B6** in 60% yield. ^{1}H NMR (400 MHz, DMSO-d_6) δ 7.23 (d, *J* = 7.4 Hz, 2H), 7.05 (t, *J* = 8.7 Hz, 1H), 6.87-6.80 (m, 2H), 4.33 (d, *J* = 8.8 Hz, 2H), 4.03-3.96 (m, 2H), 1.70-1.62 (m, 3H), 1.47-1.33 (m, 13H). MS (ESI, positive ion) m/z: 292.2 $[M + H]^+$.

(4-(2-cyclopropylethoxy)phenyl)methanamine ***(B7)*** To stirred solution of the intermediate **B6** (0.1 g, 0.34 mmol) in 1,4-dioxane (10 mL) at room temperature was added 4 N HCl in dioxane (15 mL). The reaction mixture was stirred at 80°C for 2 h. After complete conversion of the starting material, the solvent was removed under vacuum and used for next step without further purification.

5-bromo-N-(4-(2-cyclopropylethoxy)benzyl)picolinamide ***(B8)*** A THF solution (30 mL) containing 5-bromopicolinic acid (0.10 g, 0.50 mmol, Energy Chemical), **B7** (0.096 g, 0.50 mmol), DMAP (0.112 g, 1.00 mmol, Energy Chemical) and T_3P (50 wt % in EA, 0.23 mL, Accela ChemBio Co., Ltd.) was stirred at 45 °C for 2 h. The reaction mixture was removed under reduced pressure and the residue was purified through silica gel chromatography (petroleum ether/ethyl acetate = 1:3) to afford the title compound as a white solid. Yield 58% ^{1}H NMR (400 MHz, DMSO-d_6) δ 8.65-8.57 (m, 2H), 8.04 (d, *J* = 7.4 Hz, 1H), 7.93 (dd, *J* = 7.5, 1.5 Hz, 1H), 7.25 (d, *J* = 7.6 Hz, 2H), 6.91-6.79 (m, 2H), 4.50 (t, *J* = 9.8 Hz, 2H), 4.04 (t, *J* = 7.0 Hz, 2H), 1.69 – 1.55 (m, 3H), 1.47-1.31 (m, 4H). MS (ESI, positive ion) m/z: 376.1 $[M + H]^+$.

6-(6-((4-(2-cyclopropylethoxy)benzyl)carbamoyl)pyridin-3-yl)-N-methyl-1H-indazole-3-carboxamide (RI-413) Intermediate **B3** (50 mg, 0.17 mmol), intermediate **B8** (64 mg, 0.17 mmol), $PdCl_2(dppf){\cdot}CH_2Cl_2$ adduct(14 mg, 0.017mmol, Bide Pharmatech Ltd.), tricyclohexylphosphane (0.48 mg, 0.017 mmol, Energy Chemical) and Cs_2CO_3 (140 mg, 0.43 mmol, Energy Chemical) were dissolved in 1,4-dioxane and water (v/v, 6/1, Energy Chemical) at room temperature and heated at 100 °C for 12 h under nitrogen protection. After the reaction was complete as indicated by TLC, the reaction mixture was filtered through a Celite bed, and the filtrate was concentrated and purified by silica

gel chromatography (petroleum ether/ethyl acetate = 1:10) to afford the title compound as a pale-yellow solid. Yield 48%. ^{1}H NMR (400 MHz, DMSO-d_6) δ 13.76 (s, 1H), 9.02 (s, 1H), 8.47-8.28 (m, 3H), 8.15 (d, J = 8.1 Hz, 1H), 7.96 (s, 1H), 7.65 (dd, J = 8.6, 1.5 Hz, 1H), 7.27 (d, J = 8.6 Hz, 2H), 6.88 (d, J = 8.6 Hz, 2H), 3.96 (d, J = 6.6 Hz, 2H), 2.84 (d, J = 4.7 Hz, 3H), 1.58 (t, J = 6.7 Hz, 2H), 0.47-0.36 (m, 2H), 0.13-0.09 (m, 2H). ^{13}C NMR (101 MHz, DMSO-d_6) δ 164.05, 163.04, 158.09, 149.43, 147.44, 142.05, 139.02, 138.64, 136.62, 135.15, 131.89, 129.23, 122.95, 122.57, 122.17, 121.89, 114.68, 109.67, 67.98, 42.36, 34.05, 26.02, 8.13, 4.57. HRMS (m/z): calculated for $C_{27}H_{27}N_5O_3$ $^+$ [M+H_2O+H] $^+$ 488.30441; found, 488.30063. HPLC purity: 95.31%, t_R = 5.02 min.

Procedure for the Synthesis of RI-539

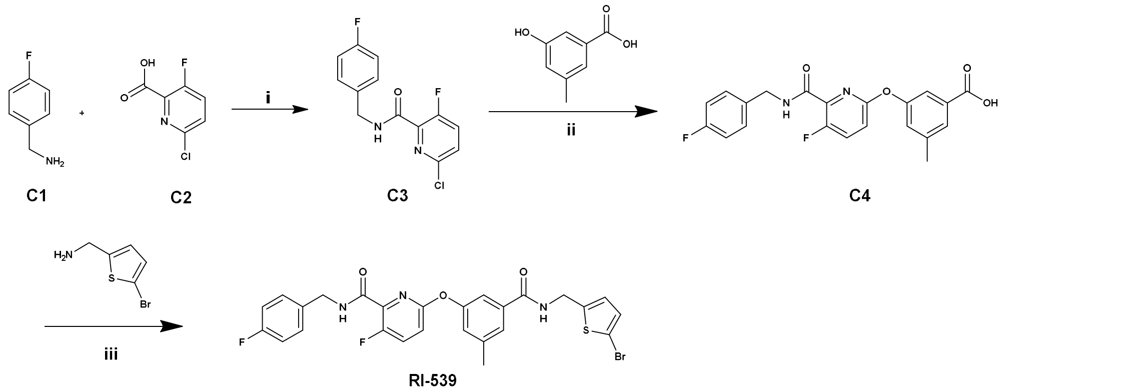

Reagents and conditions: (i) HOBt, EDCI, DIEA, DMF, 65 °C, 3 h, 67%; (ii) K_2CO_3, DMF, 120 °C, 4 h; (iii) T_3P, THF, 40 °C, 1 h, 55%.

6-chloro-3-fluoro-N-(4-fluorobenzyl)picolinamide ***(C3)*** A DMF solution (30 mL) containing 6-chloro-3-fluoropicolinic acid (**C2**, 1 g, 5.68 mmol, Bide Pharmatech Ltd.), HOBt (0.87 g, 6.44 mmol, Bide Pharmatech Ltd.) and EDCI (2.18 g, 11.36 mmol,) Bide Pharmatech Ltd. was stirred at room temperature for 30 min. DIEA (1.65 mL, 11.36 mmol, Energy Chemical) and (4-fluorophenyl)methanamine (**C1**, 0.71 g, 5.68 mmol, Energy Chemical) was then added to the reaction mixture. After 3 h the solvent was concentrated in vacuo and the residue was partitioned between DCM (20 mL) and saturated aqueous sodium thiosulfate solution (20 mL). The organic layer was dried

over Na_2SO_4, filtered, and concentrated in vacuo to give the yellow oil that was purified by silica gel chromatography (petroleum ether/ethyl acetate = 3/1) to afford **C3** as a white solid. Yield 67%. ^{1}H NMR (400 MHz, DMSO-d_6) δ 8.52 (t, J = 10.1 Hz, 1H), 7.53 – 7.21 (m, 4H), 7.18-6.99 (m, 2H), 4.53-4.49 (m, 2H). MS (ESI, positive ion) m/z: 283.0 $[M + H]^+$.

3-((5-fluoro-6-((4-fluorobenzyl)carbamoyl)pyridin-2-yl)oxy)-5-methylbenzoic acid ***(C4)*** To a suspension of 711 mg (2.73 mmol) of 6-chloro-3-fluoro-*N*-(4-fluorobenzyl)picolinamide (**C3**, Bide Pharmatech Ltd.) in 20 mL of DMF, 415 mg (2.73 mmol) of 3-hydroxy-5-methylbenzoic acid (Bide Pharmatech Ltd.) and K_2CO_3 (942 mg, 6.82 mmol, Energy Chemical) were added. Then, the mixture was heated to 120 °C for 4 h. The completion of the reaction was confirmed by TLC. Then the solvent was concentrated and the residue was treated by H_2O (10 mL) and acidified to pH ~3 using hydrochloric acid. The aqueous layer was extracted with ethyl acetate and the combined organic layers were concentrated. The crude intermediate **C4** was used to next step without further purification.

6-(3-(((5-bromothiophen-2-yl)methyl)carbamoyl)-5-methylphenoxy)-3-fluoro-N-(4-fluorobenzyl)picolinamide (RI-539) To a solution of the crude product **C4** (1.11 g, 2.79 mmol) in THF was added the (5-bromothiophen-2-yl)methanamine (0.48 g, 2.52 mmol, Energy Chemical), DMAP (0.37 g, 3.03 mmol, Energy Chemical) and T_3P (50 wt % in EA, 1.55 mL, Energy Chemical) sequentially. The solution was stirred at 40 °C for 1 h. The completion of the reaction was confirmed by TLC. Then, the reaction mixture was removed under reduced pressure and the residue was purified through silica gel chromatography (petroleum ether/ethyl acetate = 1:5) to afford the final compound as a white solid. Yield 55% ^{1}H NMR (400 MHz, Chloroform-*d*) *δ* 8.06 (s, 1H), 7.74 (s, 1H), 7.36-7.24 (m, 4H), 7.20 (d, J = 8.6 Hz, 1H), 7.11 (d, J = 2.2 Hz, 1H), 6.98 (td, J = 8.6 Hz, 2H), 6.79 (dd, J = 3.7, 1.4 Hz, 1H), 6.68 (d, J =6.9 Hz, 2H), 4.56 (d, J = 5.8 Hz, 2H), 4.49 (d, J = 6.1 Hz, 2H), 2.24 (s, 3H). ^{13}C NMR (101 MHz, Chloroform-*d*) *δ* 166.47, 163.41, 162.18, 160.97, 155.83, 152.45, 143.50, 140.72, 139.85, 135.79, 133.70, 132.25, 129.54, 129.31, 128.44, 126.53, 123.94, 122.49, 115.65, 115.43, 114.23,

111.56, 42.67, 38.82, 21.28. HRMS (m/z): calculated for $C_{26}H_{20}BrF_2N_3O_3S^+$ $[M+H_2O+H]^+$ 590.01770; found, 590.01283. HPLC purity: 96.60%, t_R = 5.77 min.

Procedure for the Synthesis of RI-985

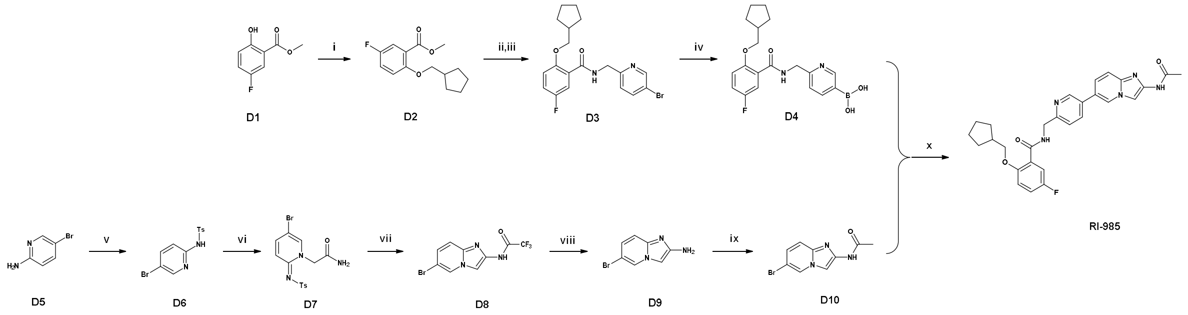

Reagents and conditions: (i) (bromomethyl)cyclopentane, K_2CO_3, DMF, 70 °C, 3 h, 74%; (ii) NaOH, H_2O/MeOH, 50 °C, 6 h; (iii) (5-bromopyridin-2-yl)methanamine, T_3P, DMAP, THF, rt, overnight, 75%; (iv) diboron pinacol ester, Pd(dppf)Cl_2, KOAc, 1,4-dioxane, 95 °C, 2 h, 40%; (v) TsCl, pyridine, 95 °C, overnight; (vi) 2-iodoacetamide, DIPEA, DMF, rt, overnight, 80%; (vii) TFAA, DCM, 50 °C, overnight; (viii) NaOH, EtOH, 80 °C, overnight; (ix) acetic acid, T_3P, DIPEA, THF, 80 °C, overnight, 79%; (x) $PdCl_2$(dppf)·CH_2Cl_2 adduct, tricyclohexylphosphane, Cs_2CO_3, 1,4-dioxane/water (v/v, 10/1), 100 °C, 12 h, 37%.

Methyl 2-(cyclopentylmethoxy)-5-fluorobenzoate ***(D2)*** A DMF solution (15mL) containing methyl 5-fluoro-2-hydroxybenzoate (1.20 g, 7.1 mmol, Bide Pharmatech Ltd.), (bromomethyl)cyclopentane (1.09 mL, 8.5 mmol, Energy Chemical) and K_2CO_3 (2.81 g, 21.3 mmol, Energy Chemical) was stirred at 70 °C for 3 h. The reaction mixture was then partitioned between DCM and water. The organic layer was concentrated under reduced pressure and the residue was purified through silica gel chromatography (petroleum ether/ethyl acetate = 3:1) to afford the title compound as a white solid. Yield 74%. ^{1}H NMR (400 MHz, Chloroform-*d*) δ 7.75 (d, J = 8.0 Hz, 1H), 7.15 (t, J = 7.7 Hz, 1H), 7.02 (d, J = 7.5 Hz, 1H), 3.94 (d, J = 6.9 Hz, 2H), 3.87 (s, 3H), 2.10 -1.98 (m, 1H), 1.71-1.48 (m, 8H). MS (ESI, positive ion) m/z: 253.1 $[M + H]^+$.

N-((5-bromopyridin-2-yl)methyl)-2-(cyclopentylmethoxy)-5-fluorobenzamide ***(D3)*** 2 N aqueous NaOH solution (10.0 mL, 20.0 mmol) was added to a solution of the methyl

2-(cyclopentylmethoxy)-5-fluorobenzoate (**D2**, 1.00 g, 2.46 mmol) in MeOH (5.0 mL). This mixture was stirred at 50 °C for 4 h and evaporated under reduced pressure. The residue was diluted with water and acidified with an aqueous solution of hydrochloric acid (3 N). This aqueous mixture was extracted with ethyl acetate (2 × 50 mL), and the combined organic extracts were washed with brine (50 mL), dried over sodium sulphate, filtered, and evaporated under reduced pressure. The residue, (5-bromopyridin-2-yl)methanamine (0.41 g, 2.21mmol, Energy Chemical), T_3P (50 wt % in EA, 2.63 mL, 4.42mmol, Energy Chemical) and DMAP (0.54 g, 4.42mmol, Energy Chemical) were then dissolved in THF and stirred at rt overnight. The solvent was removed under reduced pressure and the residue was purified through silica gel chromatography (petroleum ether/ethyl acetate = 3:1) to afford the title compound as a white solid. Yield 75% ^{1}H NMR (400 MHz, Chloroform-*d*) δ 8.48 (d, J = 1.5 Hz, 1H), 8.32 (t, J = 9.1 Hz, 1H), 7.76 (d, J = 7.8 Hz, 2H), 7.59 (d, J = 7.5 Hz, 1H), 7.20-7.12 (m, 1H), 7.01 (d, J = 7.5 Hz, 1H), 4.64 (d, J = 9.0 Hz, 2H), 3.99 (d, J = 7.0 Hz, 2H), 2.16-2.01 (m, 1H), 1.70-1.45 (m, 8H). MS (ESI, positive ion) m/z: 408.1 $[M + H]^+$.

(6-((2-(cyclopentylmethoxy)-5-fluorobenzamido)methyl)pyridin-3-yl)boronic acid ***(D4)*** A flask charged with **D3** (0.24g, 0.59mmol), diboron pinacol ester (0.45g, 1.77mmol, Energy Chemical), KOAc(0.15g, 1.48mmol, Energy Chemical) and Pd(dppf)Cl_2 (43mg, 0.059 mmol, Bide Pharmatech Ltd.) was flushed with nitrogen. Anhydrous 1,4-dioxane (10 mL, Energy Chemical) was then added. After being stirred at 95 °C for 8 h, the mixture was filtrated and the filtrate was concentrated in vacuo, and the residue was purified through silica gel chromatography (petroleum ether/ethyl acetate = 1:3) to afford the title compound as a yellow solid. Yield 40% ^{1}H NMR (400 MHz, DMSO-d_6) δ 8.80 (d, J = 1.5 Hz, 1H), 8.50 (s, 2H), 8.28 (t, J = 9.1 Hz, 1H), 8.10 (dd, J = 7.5, 1.5 Hz, 1H), 7.80 (dd, J = 8.0, 1.4 Hz, 1H), 7.45 (d, J = 7.5 Hz, 1H), 7.25 (d, J = 7.8 Hz, 1H), 7.02 (dd, J = 7.5, 4.9 Hz, 1H), 4.64 (d, J = 9.1 Hz, 2H), 3.97 (d, J = 7.0 Hz, 2H), 2.09 (dt, J = 7.1, 3.1 Hz, 1H), 1.67-1.51 (m, 8H). MS (ESI, positive ion) m/z: 372.2 $[M + H]^+$.

N-(5-bromopyridin-2-yl)-4-methylbenzenesulfonamide ***(D6)*** A mixture of 5-

bromopyridin-2-amine (**D5,** 17.3 g, 100.0 mmol) and TsCl (22.9 g, 120.0 mmol, Energy Chemical) in 80 mL pyridine (Energy Chemical) was heated to 90 °C overnight. After cooled to r.t., the solution was removed by vacuum. 50 ml water was poured into the residue. The resulting precipitate was filtered. The filter cake was washed with water, dried over vacuum to give a white crude solid which was taken onto the next step without further purification.

2-(5-bromo-2-(tosylimino)pyridin-1(2H)-yl)acetamide ***(D7)*** A mixture of **D6** (34.56 g, crude, 90 mmol), DIEA (24.0 ml, 135 mmol, Energy Chemical) and 2-iodoacetamide (25.0 g, 135 mmol, Energy Chemical) in 50 mL DMF (Energy Chemical) was stirred at rt overnight. After that, sufficient water was added. The resulting precipitate was filtered and the cake was washed with water, dried over vacuum to give a brown solid. Yield 80%. ^{1}H NMR (400 MHz, DMSO-d_6) δ 7.84-7.78 (m, 2H), 7.71 (d, J = 1.5 Hz, 1H), 7.53 (d, J = 9.9 Hz, 1H), 7.32 (d, J = 7.6 Hz, 2H), 7.22 (dd, J = 9.8, 1.5 Hz, 1H), 7.09 (s, 2H), 4.54-4.35 (m, 2H), 2.40 (d, J = 2.8 Hz, 3H). MS (ESI, positive ion) m/z: 384.0 $[M + H]^+$.

N-(6-bromoimidazo[1,2-a]pyridin-2-yl)-2,2,2-trifluoroacetamide ***(D8)*** To a stirred solution of **D7** (9 g, 23 mmol) in 80 mL DCM at rt , TFAA (16.2 ml, 115 mmol, Energy Chemical) was added. The mixture was stirred at 50 °C overnight. After cooled to rt , the mixture was basified by sat. aq. Na_2CO_3 to pH= 7. The resulting precipitate was filtered. The cake was washed with water. The filtrate was extracted with DCM. The organic phases were dried over Na_2SO_4 and concentrated under reduced pressure to afford the title compound as a crude brown solid. The solid was used for next step without further purification.

6-bromoimidazo[1,2-a]pyridin-2-amine ***(D9)*** A mixture of **D8** (5 g, crude, 15.3 mmol) in 2 mol/L NaOH/EtOH (2:1) was stirred at 70 °C overnight. After cooled to r.t, the aqueous phase was extracted with ethyl acetate. The organic phases were dried over Na2SO4, filtered, and purified by purified through silica gel chromatography (petroleum ether/ethyl acetate = 1:5) to afford the title compound as a brown oil. ^{1}H NMR (400 MHz, DMSO-d_6) δ 8.60 (s, 1H), 7.32-7.05 (m, 2H), 7.00 (s, 1H), 5.22 (s,

2H). MS (ESI, positive ion) m/z: 211.9 $[M + H]^+$.

N-(6-bromoimidazo[1,2-a]pyridin-2-yl)acetamide ***(D10)*** A THF solution (10 mL) containing **D9** (0.33 g, 1.58 mmol), 2-(4-fluorophenyl)acetic acid (0.09 g, 1.58 mmol, Accela ChemBio Co., Ltd.), T_3P (50 wt % in EA, 1.88 mL, 3.16 mmol, Energy Chemical) and DMAP (0.18 g, 3.16 mmol, Energy Chemical) was stirred at 50 °C for 6 h. The reaction mixture was removed under reduced pressure and the residue was purified through silica gel chromatography (petroleum ether/ethyl acetate = 1:3) to afford the title compound as a brown solid. Yield 79%. ^1H NMR (400 MHz, DMSO-d_6) δ 10.64 (s, 1H), 9.43 (d, J = 1.3 Hz, 1H), 7.62-7.55 (m, 2H), 7.32-7.22 (m, 1H), 2.12 (s, 3H). MS (ESI, positive ion) m/z: 253.9 $[M + H]^+$.

N-((5-(2-acetamidoimidazo[1,2-a]pyridin-6-yl)pyridin-2-yl)methyl)-2-(cyclopentylmethoxy) -5-fluorobenzamide (RI-985) Intermediate **D4** (93 mg, 0.25mmol), intermediate **D10** (63 mg, 0.25 mmol), $PdCl_2(dppf)\cdot CH_2Cl_2$ adduct (21 mg, 0.025 mmol, Bide Pharmatech Ltd.), tricyclohexylphosphine (7 mg, 0.025 mmol, Energy Chemical) and Cs_2CO_3 (244 mg, 0.75 mmol, Energy Chemical) were dissolved in 1,4-dioxane and water (v/v, 10/1) at room temperature and heated to 100 °C for 12 h under nitrogen protection. After the reaction was complete as indicated by TLC, the reaction mixture was filtered through a Celite bed, and the filtrate was concentrated and purified by silica gel chromatography (dichloromethane/methanol = 20:1) to afford the title compound as a dark yellow solid. After washing by Et_2O, the desired product was obtained. Yield 37% ^1H NMR (400 MHz, DMSO-d_6) δ 10.73 (s, 1H), 9.00 (s, 1H), 8.95 (t, J = 5.3 Hz, 1H), 8.86 (s, 1H), 8.14 (s, 1H), 8.12-8.06 (m, 1H), 7.64-7.57 (m, 2H), 7.54 (d, J = 9.0 Hz, 2H), 7.38-7.30 (m, 1H), 7.22 (dd, J = 9.3, 4.3 Hz, 1H), 4.69 (d, J = 5.2 Hz, 2H), 4.02 (d, J = 7.1 Hz, 2H), 2.45-2.32 (m, 1H), 2.09 (s, 3H), 1.77-1.66 (m, 2H), 1.60-1.41 (m, 4H), 1.35-1.25 (m, 2H). ^{13}C NMR (101 MHz, DMSO-d_6) δ 169.00, 164.56, 155.74, 153.17, 146.27, 140.37, 135.01, 131.57, 124.43, 123.18, 122.67, 121.92, 119.16, 118.92, 117.42, 117.17, 115.17, 113.41, 73.57, 44.40, 38.44, 28.93, 24.80, 22.10. HRMS (m/z): calculated for $C_{28}H_{28}FN_5O_3^+$ $[M+H]^+$ 502.22024; found, 502.22504. HPLC purity: 95.31%, t_R = 6.11 min.

Procedure for the Synthesis of RI-962

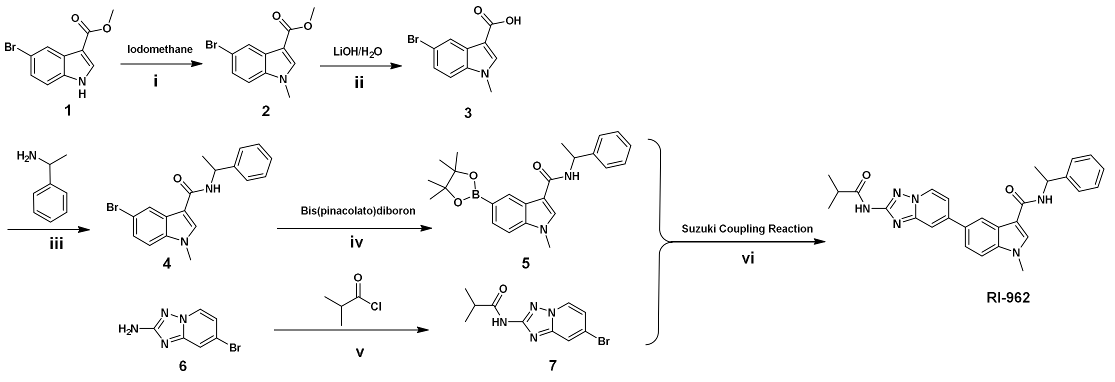

Reagents and conditions: (i) Iodomethane, Cs_2CO_3, DMF, 80 °C, 1.5 h, 90%; (ii) LiOH, H_2O/MeOH, 50 °C, 3 h; (iii) 1-phenylethan-1-amine, HATU, DIEA, DMF, rt, 5 h, 50%; (iv) diboron pinacol ester, Pd(dppf)Cl_2, KOAc, 1,4-dioxane, 95 °C, 3 h, 44%; (v) Isobutyryl chloride, pyridine, 0 °C-rt, 2 h, 70%; (vi) $PdCl_2$(dppf)·CH_2Cl_2 adduct, tricyclohexylphosphane, Cs_2CO_3, 1,4-dioxane/water (v/v, 10/1), 100 °C, 8 h, 40%.

Methyl 5-bromo-1-methyl-1H-indole-3-carboxylate ***(2)*** A flask was charged with methyl methyl 5-bromo-1H-indole-3-carboxylate (500 mg, 1.97 mmol, Bide Pharmatech Ltd.), Cs_2CO_3 (1.93 g, 5.91 mmol, Energy Chemical), and DMF (20 mL, Energy Chemical). Iodomethane (0.85 mL, 2.71 mmol, Energy Chemical) was added, and the resulting mixture was heated to 80°C and stirred for 1.5 h. The reaction mixture was cooled to rt, and the solid crude product was concentrated and purified by silica gel chromatography (petroleum ether/ethyl acetate = 3:1) to afford the compound 2 as a white solid. Yield 90% ^{1}H NMR (400 MHz, DMSO-d_6) δ 8.39-8.31 (m, 1H), 7.86 (d, J = 0.7 Hz, 1H), 7.59-7.45 (m, 2H), 3.85 (s, 3H), 3.75 (s, 3H). ESI-MS (m/z): 267.9 (M + H)$^+$

5-bromo-1-methyl-1H-indole-3-carboxylic acid ***(3)*** A solution of methyl 5-bromo-1-methyl-1*H*-indole-3-carboxylate (**2**) (200 mg) in MeOH (30 mL) was treated with 2 M LiOH aqueous solution (30 mL). The reaction mixture was stirred for 3 h at 50 °C. The completion of the reaction was confirmed by TLC, then the MeOH was concentrated and the solution was acidified to pH ~3 using hydrochloric acid. The aqueous layer was

extracted with DCM and the combined organic layers were concentrated. The crude intermediate **3** was used to next step without further purification.

5-bromo-1-methyl-N-(1-phenylethyl)-1H-indole-3-carboxamide ***(4)*** To a solution of the 5-bromo-1-methyl-1*H*-indole-3-carboxylic acid **3** (0.45 g, 1.77 mmol) in DMF was added the HATU (0.81 g, 2.1 mmol, Energy Chemical) sequentially. The solution was stirred for 30 mins, and then DIEA (0.73 mL, 4.42 mmol, Energy Chemical) was added. Next, the 1-phenylethan-1-amine (0.21 g 1.77 mmol, Macklin Biochemical Co., Ltd) was added, and the reaction mixture was stirred for 5 h under room temperature. The reaction mixture was concentrated and extracted with saturated NH_4Cl solution, saturated $NaHCO_3$ solution and brine. The organic phase was dried over Na_2SO_4 and concentrated, and the residue was purified by column chromatography (ethyl acetate / methanol = 5:1) to afford **4** as a white solid. Yield 50%. ^{1}H NMR (400 MHz, DMSO-d_6) δ 8.38 (d, J = 8.8 Hz, 1H), 8.20 (d, J = 1.5 Hz, 1H), 7.91 (s, 1H), 7.56-7.46 (m, 2H), 7.39 – 7.19 (m, 5H), 5.14 (dt, J = 8.8, 0.9 Hz, 1H), 3.78 (s, 3H), 1.51 (d, J = 6.8 Hz, 3H). ESI-MS (m/z): 357.1 $(M + H)^+$

1-methyl-N-(1-phenylethyl)-5-(4,4,5,5-tetramethyl-1,3,2-dioxaborolan-2-yl)-1H-indole-3-carboxamide ***(5)*** A flask charged with **4** (0.8 g, 2.24 mmol), diboron pinacol ester (1.14 g, 4.48 mmol, Bide Pharmatech Ltd.), KOAc(0.44 g, 4.48 mmol, Energy Chemical) and Pd(dppf)Cl_2 (160 mg, 0.22 mmol, Bide Pharmatech Ltd.) was flushed with nitrogen. Anhydrous 1,4-dioxane (25 mL, Energy Chemical) was then added. After being stirred at 95 °C for 3 h, solvent was filtrated and the filtrate was concentrated in vacuo, then the residue was purified through silica gel chromatography (petroleum ether/ethyl acetate = 1:1) to afford the title compound as a white solid. Yield 44% ^{1}H NMR (400 MHz, DMSO-d_6) δ 8.50 (d, J = 8.8 Hz, 1H), 7.82 (dt, J = 1.3, 0.5 Hz, 1H), 7.65-7.56 (m, 2H), 7.51 (s, 1H), 7.40-7.16 (m, 5H), 5.14 (dt, J = 8.8, 1.1 Hz, 1H), 3.77 (s, 3H), 1.51 (d, J = 6.8 Hz, 3H), 1.24 (s, 12H). ESI-MS (m/z): 405.2 $(M + H)^+$

N-(7-bromo-[1,2,4]triazolo[1,5-a]pyridin-2-yl)isobutyramide ***(7)*** To a pyridine solution (10 mL) containing 7-bromo-[1,2,4]triazolo[1,5-*a*]pyridin-2-amine (0.20 g, 1.58 mmol, Bide Pharmatech Ltd.) and triethylamine (0.55 ml, 3.95 mmol, Energy

Chemical) was added isobutyryl chloride (0.21 mL , 2.0 mmol, Energy Chemical) dropwise at 0 °C for 10 min. The reaction mixture was stirred at 50 °C for 4 h. After the reaction was complete as indicated by TLC, the mixture was removed under reduced pressure and the residue was purified through silica gel chromatography (petroleum ether/ethyl acetate = 1:2) to afford the title compound as a white solid. Yield 70%. ^{1}H NMR (400 MHz, DMSO-d_6) δ 10.79 (s, 1H), 8.81 (d, J = 7.1 Hz, 1H), 8.04 (d, J = 2.1 Hz, 1H), 7.30 (d, J = 7.2 Hz, 1H), 2.77 (s, 1H), 1.15-1.03 (m, 6H). ESI-MS (m/z): 283.0 $(M + H)^+$

5-(2-isobutyramido-[1,2,4]triazolo[1,5-a]pyridin-7-yl)-1-methyl-N-(1-phenylethyl)-1H-indole-3-carboxamide (RI-962) Intermediate **5** (54 mg, 0.12 mmol), intermediate **5** (32 mg, 0.12 mmol), $PdCl_2(dppf)\cdot CH_2Cl_2$ adduct(9 mg, 0.012 mmol, Bide Pharmatech Ltd.), tricyclohexylphosphane(2 mg, 0.006 mmol, Bide Pharmatech Ltd.) and Cs_2CO_3 (98 mg, 0.30 mmol, Energy Chemical) were dissolved in 1,4-dioxane and water (v/v, 8/1) at room temperature and heated at 100 °C for 12 h under nitrogen protection. After the reaction was complete as indicated by TLC, the reaction mixture was filtered through a Celite bed, and the filtrate was concentrated and purified by silica gel chromatography (petroleum ether/ethyl acetate = 1:8) to afford the title compound as a yellow solid. After washing by Et_2O, the desired product was obtained. Yield 40%. ^{1}H NMR (400 MHz, DMSO-d_6) δ 10.73 (s, 1H), 8.84 (d, J = 7.1 Hz, 1H), 8.54 (d, J = 1.8 Hz, 1H), 8.36 (d, J = 8.1 Hz, 1H), 8.22 (s, 1H), 7.86 (d, J = 1.9 Hz, 1H), 7.77-7.62 (m, 2H), 7.50-7.39 (m, 3H), 7.33 (t, J = 7.5 Hz, 2H), 7.22 (t, J = 7.3 Hz, 1H), 5.26-5.14 (m, 1H), 3.90 (s, 3H), 2.79 (s, 1H), 1.49 (d, J = 7.1 Hz, 3H), 1.14-1.03 (m, 6H). ^{13}C NMR (101 MHz, DMSO-d_6) δ 163.75, 159.48, 145.98, 137.50, 133.53, 130.37, 128.97, 128.66, 127.64, 126.94, 126.54, 121.74, 120.36, 113.23, 111.55, 110.62, 48.13, 33.61, 23.01, 19.79. HRMS (m/z): calculated for $C_{28}H_{28}N_6O_2^+$ $[M+H]^+$ 481.2307; found, 481.2375. HPLC purity: 99.10%, t_R = 6.10 min.

Procedure for the Synthesis of RI-056

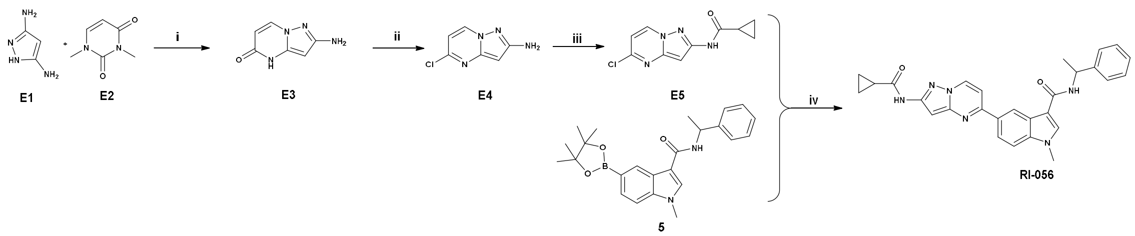

Reagents and conditions: (i) 1,3-dimethyl-1,2,3,4-tetrahydropyrimidine-2,4-dione, NaOEt, EtOH, 80 °C, 15 h; (ii) $POCl_3$, 90 °C, 1 h; (iii) Cyclopropanecarbonyl Chloride, pyridine, 0 °C to rt, 2 h, 60%; (iv) $PdCl_2$(dppf)·CH_2Cl_2 adduct, tricyclohexylphosphane, Cs_2CO_3, 1,4-dioxane/water (v/v, 10/1), 100 °C, 10 h, 35%.

2-aminopyrazolo[1,5-a]pyrimidin-5(4H)-one ***(E3)*** A flask was charged with 1*H*-pyrazole-3,5-diamine (**E1**, 2.50 g, 25.5 mmol, Bide Pharmatech Ltd.), 1,3-dimethyl-1,2,3,4-tetrahydropyrimidine-2,4-dione (**E2**, 3.60 g, 25.5 mmol, Bide Pharmatech Ltd.) and EtOH (50 mL). NaOEt (21 wt % in EtOH, 8 mL) was added, and the resulting mixture was heated to 80 °C and stirred for 15 h. The reaction mixture was cooled to rt, and the solid crude product **E3** was isolated by filtration, dried under reduced pressure, and taken immediately onto the next step without further purification.

5-chloropyrazolo[1,5-a]pyrimidin-2-amine ***(E4)*** The crude product (**E3**, 660 mg) from the previous step was cautiously added to $POCl_3$(4 mL, Energy Chemical) at 0 °C. The resulting mixture was heated to 80°C and stirred for 30 min. The reaction was carefully poured onto ice and quenched by the slow addition of K_2CO_3(excess), with stirring, and diluted with DCM (100 mL). The aqueous layer was separated and back-extracted with additional DCM (2×75 mL). The organic layers were combined, washed with H_2O (100 mL) and brine (100 mL), and dried over $MgSO_4$. Concentration under reduced pressure furnished the title compound that was of sufficient purity to use in subsequent steps. ^{1}H NMR (400 MHz, DMSO-d_6) δ 8.63 (d, J = 7.5 Hz, 1H), 6.72 (d, J = 7.5 Hz, 1H), 6.13 (s, 1H), 5.80 (s, 2H). ESI-MS (m/z): 169.0 $(M + H)^+$

N-(5-chloropyrazolo[1,5-a]pyrimidin-2-yl)cyclopropanecarboxamide ***(E5)*** To a stirred suspension of 5-chloropyrazolo[1,5-*a*]pyrimidin-2-amine (500 mg, 2.97 mmol, Bide Pharmatech Ltd.) and triethylamine (1.24 mL, 8.91 mmol, Energy Chemical) in

pyridine (15 mL, Energy Chemical), Cyclopropanecarbonyl Chloride (310 mg, 2.97 mmol, Accela ChemBio Co., Ltd.) was added dropwise at 0 °C. Then, the reaction mixture was heated to room temperature and stirred for 2 h. The solvent was concentrated in vacuo and the residue was partitioned between DCM (20 mL) and saturated aqueous sodium carbonate solution (20 mL). The organic layer was washed with H_2O and saturated sodium chloride solution, dried over $MgSO_4$, filtered, and concentrated in vacuo to give the yellow oil that was purified by silica gel chromatography (ethyl acetate/petroleum ether = 1:1) to afford **E5** as a pale-white solid. Yield 60%. ^{1}H NMR (400 MHz, DMSO-d_6) δ 9.58 (s, 1H), 8.68 (d, *J* = 7.5 Hz, 1H), 6.86-6.74 (m, 2H), 2.33 (t, *J* = 7.0 Hz, 1H), 0.92-0.93 (m, 4H). ESI-MS (m/z): 237.1 $(M + H)^+$

5-(2-(cyclopropanecarboxamido)pyrazolo[1,5-a]pyrimidin-5-yl)-1-methyl-N-(1-phenylethyl)-1H-indole-3-carboxamide (RI-056) Intermediate compounds **E5** (59 mg, 0.25 mmol), Cs_2CO_3(200.0 mg, 0.63 mmol), $PdCl_2$(dppf)·CH_2Cl_2 adduct (20 mg, 0.025 mmol, Bide Pharmatech Ltd.), tricyclohexylphospine(3.5 mg, 0.013 mol, Energy Chemical) and Intermediate compounds **5** (100.0 mg, 0.25 mmol) were stirred in 1,4-dioxane (Energy Chemical) and water (25 mL, v/v, 8/1) and heated at 95 °C for 10 h under nitrogen protection. The reaction mixture was subsequently evaporated, and the residue was purified by chromatography (petroleum ether / ethyl acetate = 1:3) to afford 5-(2-(cyclopropanecarboxamido)pyrazolo[1,5-*a*]pyrimidin-5-yl)-1-methyl-*N*-(1-phenylethyl)-1*H*-indole-3-carboxamide as a yellow solid. Yield 35%. ^{1}H NMR (400 MHz, DMSO-d_6) *δ* 11.17 (s, 1H), 8.94 (d, *J* = 8.2 Hz, 1H), 8.37 (d, *J* = 8.1 Hz, 1H), 8.23 (s, 1H), 8.14-8.01 (m, 1H), 7.64 (d, *J* = 8.7 Hz, 1H), 7.61-7.48 (m, 2H), 7.34 (d, *J* = 7.4 Hz, 5H), 6.86 (s, 1H), 5.22 (s, 1H), 3.90 (s, 3H), 1.97 (s, 1H), 1.56-1.41 (m, 3H), 1.24 (s, 2H), 0.85 (d, *J* = 7.3 Hz, 2H). ^{13}C NMR (101 MHz, DMSO-d_6) *δ* 164.11, 159.51, 150.31, 143.88, 138.70, 137.64, 137.53, 133.60, 130.39, 129.16, 128.99, 128.54, 127.71, 127.01, 126.28, 121.75, 120.47, 113.25, 111.57, 110.69, 110.62, 46.73, 30.70, 29.45, 20.97, 19.79. HRMS (m/z): calculated for $C_{28}H_{26}N_6O_2^+$ $[M+H]^+$ 479.71511; found, 479.21957. HPLC purity: 97.45%, t_R = 5.72 min.

Procedure for the Synthesis of RI-1155

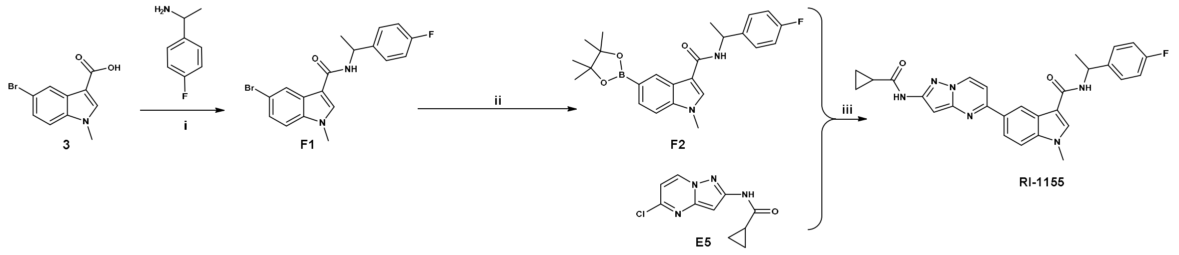

Reagents and conditions: (i) 1-phenylethan-1-amine, HATU, DIEA, DMF, rt, 5 h, 77%; (ii) diboron pinacol ester, Pd(dppf)Cl_2, KOAc, 1,4-dioxane, 95 °C, 3 h, 57%; (iii) $PdCl_2$(dppf)·CH_2Cl_2 adduct, tricyclohexylphosphane, Cs_2CO_3, 1,4-dioxane/water (v/v, 10/1), 100 °C, 10 h, 40%.

*5-bromo-N-(1-(4-fluorophenyl)ethyl)-1-methyl-1H-indole-3-carboxamide **(F1)*** **F1** was prepared by the same method as that for **4** as a yellow solid. Yield 77%. ^{1}H NMR (400 MHz, DMSO-*d6*) δ 8.16-8.09 (m, 2H), 7.90 (s, 1H), 7.59-7.39 (m, 2H), 7.23 (d, *J* = 6.7, 2H), 7.10-6.99 (m, 2H), 5.16-5.05 (m, 1H), 3.79 (s, 3H), 1.52 (d, *J* = 6.9 Hz, 3H). ESI-MS (m/z): 375.0 (M + H)$^+$.

*N-(1-(4-fluorophenyl)ethyl)-1-methyl-5-(4,4,5,5-tetramethyl-1,3,2-dioxaborolan-2-yl)-1H-indole-3-carboxamide **(F2)*** **F2** was prepared by the same method as that for **4** as a white solid. Yield 57%. ^{1}H NMR (400 MHz, DMSO-d_6) δ 8.53 (d, *J* = 8.8 Hz, 1H), 7.82 (d, *J* = 6.4 Hz, 1H), 7.63-7.55 (m, 2H), 7.51 (s, 1H), 7.35-7.28 (m, 2H), 7.07-6.99 (m, 2H), 5.14 (dt, *J* = 8.8, 1.1 Hz, 1H), 3.77 (s, 3H), 1.50 (d, *J* = 6.8 Hz, 3H), 1.27 (s, 12H). ESI-MS (m/z): 423.2 (M + H)$^+$.

5-(2-(cyclopropanecarboxamido)pyrazolo[1,5-a]pyrimidin-5-yl)-N-(1-(4-fluorophenyl)ethyl)-1-methyl-1H-indole-3-carboxamide (RI-1155) RI-1155 was prepared by the same method as that for RI-056 as a white solid. Yield 40%. ^{1}H NMR (400 MHz, DMSO-d_6) δ 11.17 (s, 1H), 8.94 (d, *J* = 7.2 Hz, 2H), 8.38 (d, *J* = 8.0 Hz, 1H), 8.21 (s, 1H), 8.09 (dd, *J* = 8.8, 1.8 Hz, 1H), 7.64 (d, *J* = 8.7 Hz, 1H), 7.53-7.41 (m, 3H), 7.21-7.09 (m, 2H), 6.86 (s, 1H), 5.28-5.16 (m, 1H), 3.90 (s, 3H), 1.96 (d, *J* = 5.9 Hz, 1H), 1.49 (d, *J* = 6.9 Hz, 3H), 0.96 – 0.76 (m, 4H). ^{13}C NMR (101 MHz, DMSO-

*d*6) *δ* 172.27, 163.70, 162.62, 160.21, 156.76, 153.09, 148.50, 142.12, 138.43, 135.51, 133.58, 129.88, 128.43, 127.43, 121.75, 121.13, 115.21, 111.15, 110.84, 105.06, 86.26, 47.57, 33.63, 22.94, 14.50, 8.07. HRMS (m/z): calculated for $C_{28}H_{25}FN_6O_2^+$ [M+H] $^+$ 497.20570; found, 497.21014. HPLC purity: 98.44%, t_R = 5.74 min.

Procedure for the Synthesis of RI-753

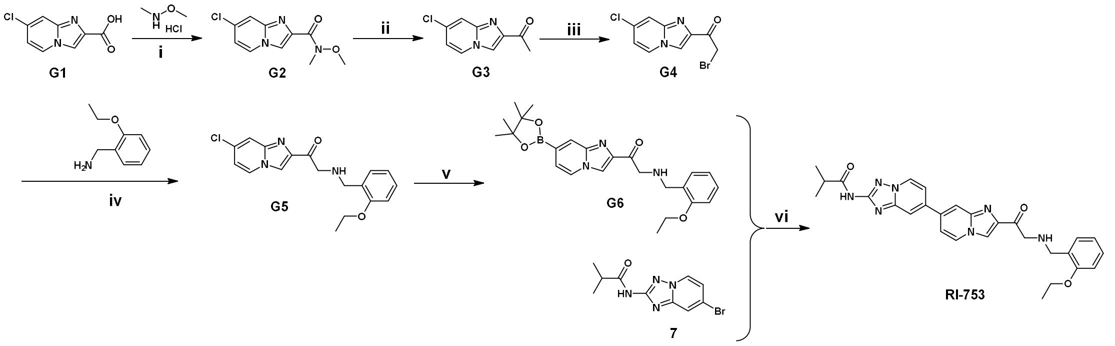

Reagents and conditions: (i) *N,O*-Dimethylhydroxylamine hydrochloride, HOBT, EDCI, DIEA, DCM, rt, 16 h, 57%; (ii) Methylmagnesium bromide, THF, 0 °C-rt, 2 h, 46%; (iii) HBr, Br_2, 70 °C, 1 h, 66%; (iv) DIEA, DMF, rt-80 °C, 3 h, 52%; (v) diboron pinacol ester, Pd(dppf)Cl_2, KOAc, 1,4-dioxane, 95 °C, 3 h, 50%; (vi) $PdCl_2$(dppf)·CH_2Cl_2 adduct, tricyclohexylphosphane, Cs_2CO_3, 1,4-dioxane/water (v/v, 10/1), 100 °C, 8 h, 41%.

7-chloro-N-methoxy-N-methylimidazo[1,2-a]pyridine-2-carboxamide ***(G2)*** To a mixture of 7-chloroimidazo[1,2-*a*]pyridine-2-carboxylic acid (**G1**, 0.5 g, 2.54 mmol, Bide Pharmatech Ltd.) in DCM (20 mL) was added DIEA (1.05 mL, 6.35 mmol, Energy Chemical), *N,O*-dimethylhydroxylamine hydrochloride (0.3 g, 3.05 mmol, Energy Chemical), 1-Hydroxybenzotriazole (0.51 g, 3.81mmol, Macklin Biochemical Co., Ltd) and EDCI (0.91 g, 5.08 mmol, Energy Chemical). The reaction mixture was stirred at room temperature for 16 h. Then, the mixture was added $NaHCO_3$ (30 mL), eluting with ethyl acetate (30 mL×3). The organic phase was concentrated in vacuo and the residue was purified by silica gel column chromatography (ethyl acetate / petroleum ether = 1:1) to afford **G2** as an off-white solid. Yield 57%. ^{1}H NMR (400 MHz, DMSO-

d$_6$) δ 8.52-8.49 (m, 1H), 8.42 (s, 1H), 7.42-7.37 (m, 1H), 6.74 (dd, *J* = 7.5, 1.5 Hz, 1H), 3.56 (s, 3H), 3.18 (s, 3H). ESI-MS (m/z): 240.0 $(M + H)^+$.

1-(7-chloroimidazo[1,2-a]pyridin-2-yl)ethan-1-one ***(G3)*** To a solution of 7-chloro-*N*-methoxy-*N*-methylimidazo[1,2-*a*]pyridine-2-carboxamide (**G2**, 1.0 g, 4.16 mmol) in dry THF (100 mL, Energy Chemical) was added dropwise a solution of Methylmagnesium bromide in THF in THF (4.5 mL, 3 M, Energy Chemical) at 0 °C under Argon, then the mixture was stirred at room temperature for 2 h. After the reactant was consumed, the reaction was quenched by saturated NH_4Cl solution (40 mL). The reaction mixture was allowed to warm up to room temperature and then poured into brine (40 mL). The organic layer was concentrated and purified by flash column chromatography (petroleum ether/ethyl acetate = 7/3) to give product **G3** as a pale-yellow solid. Yield 46%. ^{1}H NMR (400 MHz, DMSO-*d*$_6$) δ 8.49 (d, *J* = 7.6 Hz, 1H), 8.36 (s, 1H), 7.42-7.33 (m, 1H), 6.74 (dd, *J* = 7.5, 1.5 Hz, 1H), 2.57 (s, 3H). ESI-MS (m/z): 195.1 $(M + H)^+$.

2-bromo-1-(7-chloroimidazo[1,2-a]pyridin-2-yl)ethan-1-one ***(G4)*** Intermediate (**G3**) (0.873 g, 4.50 mol) in HBr 48% (50 ml) was stirred at 70°C. A solution of Br_2 (0.715 g, 4.50 mol) in HBr 48% (3 mL, Energy Chemical) was added dropwise and the reaction mixture was stirred for 1 h at 70°C. The solvent was evaporated. The residue was stirred in 2-propanone with a small amount of methanol (15/1), filtered off and dried to afford the title compound **G4** as a yellow solid. Yield 66%. ^{1}H NMR (400 MHz, DMSO-*d*$_6$) δ 8.47 (d, *J* = 7.6 Hz, 1H), 8.40 (s, 1H), 7.39-7.31 (m, 1H), 6.74 (dd, *J* = 7.4, 1.1 Hz, 1H), 4.65 (s, 2H). ESI-MS (m/z): 272.8 $(M + H)^+$.

1-(7-chloroimidazo[1,2-a]pyridin-2-yl)-2-((2-ethoxybenzyl)amino)ethan-1-one ***(G5)*** DIEA (0.28 g, 2.19 mmol, Energy Chemical) and **G4** (0.2 g, 0.73 mmol) were added to a solution of (2-ethoxyphenyl)methanamine (0.11 g, 0.73 mmol, Accela ChemBio Co., Ltd.) in DMF (10 mL). The reaction mixture was stirred at room temperature for 1 h and then heated to 80 °C for 3 h. T The solvent was concentrated in vacuo and the residue was partitioned between DCM (20 mL) and saturated aqueous sodium thiosulfate solution (20 mL). The organic layer was dried over Na_2SO_4, filtered, and

concentrated in vacuo to give the yellow oil that was purified by silica gel chromatography (petroleum ether/ethyl acetate = 1/3) to afford **G5** as a yellow solid. Yield 52%. ^{1}H NMR (400 MHz, DMSO-d_6) δ 8.49 (d, J = 7.4 Hz, 1H), 8.39 (s, 1H), 7.40-7.32 (m, 1H), 7.31-7.18 (m, 2H), 7.02 (td, J = 7.5, 1.5 Hz, 1H), 6.86 (d, J = 7.4 Hz, 1H), 6.74 (d, J = 7.5Hz, 1H), 4.44-4.13 (m, 4H), 4.11-3.98 (m, 3H). 1.47-1.38 (m, 3H). ESI-MS (m/z): 345.1 $(M + H)^+$.

2-((2-ethoxybenzyl)amino)-1-(7-(4,4,5,5-tetramethyl-1,3,2-dioxaborolan-2-yl)imidazo[1,2-a]pyridin-2-yl)ethan-1-one ***(G6)*** **G6** was prepared by the same method as that for **5** as a white solid. Yield 50%. ^{1}H NMR (400 MHz, DMSO-d_6) δ 8.41-8.34 (m, 2H), 7.52-7.48 (m, 1H), 7.32-7.27 (m, 1H), 7.21 (td, J = 7.5, 1.5 Hz, 1H), 7.02 (td, J = 7.5, 1.5 Hz, 1H), 6.85 (d, J = 7.4 Hz, 1H), 6.74 (d, J = 7.5 Hz, 1H), 4.42 (d, J = 8.5 Hz, 2H), 4.24-4.13 (m, 1H), 4.12-4.00 (m, 4H), 1.43 (t, J = 8.0 Hz, 3H), 1.24 (s, 12H). ESI-MS (m/z): 436.1 $(M + H)^+$.

N-(7-(2-((2-ethoxybenzyl)glycyl)imidazo[1,2-a]pyridin-7-yl)-[1,2,4]triazolo[1,5-a]pyridin-2-yl)isobutyramide (RI-753) RI-753 was prepared by the same method as that for RI-962 as a pale-yellow solid. Yield 41%.^{1}H NMR (400 MHz, DMSO-d_6) δ 10.02 (s, 1H), 8.62 (d, J = 6.3 Hz, 1H), 8.56 (d, J = 7.2 Hz, 1H), 8.41 (d, J = 0.7 Hz, 1H), 7.92 (d, J = 2.0 Hz, 1H), 7.27-7.12 (m, 3H), 7.02-6.94 (m, 1H), 6.87 (d, J = 7.4 Hz, 1H), 5.49 (d, J = 7.8 Hz, 1H), 4.56 (d, J = 6.3 Hz, 2H), 4.46 (d, J = 7.3 Hz, 2H), 4.09 (t, J = 7.0 Hz, 2H), 3.66-3.59 (m, 1H), 1.39 (t, J = 6.9 Hz, 3H), 1.03-0.96 (m, 1H).^{13}C NMR (101 MHz, DMSO-d_6) δ 173.43, 162.52, 159.36, 156.47, 149.34, 144.91, 140.80, 138.12, 133.38, 133.09, 128.42, 128.13, 127.89, 127.45, 125.44, 121.30, 120.53, 120.39, 114.86, 113.59, 113.15, 111.82, 63.75, 38.06, 15.24, 14.36.HRMS (m/z): calculated for $C_{28}H_{29}N_7O_3^+$ $[M+H]^+$ 512.17615; found, 512.17578. HPLC purity: 95.82%, t_R = 6.42 min.

^{1}H NMR spectra, ^{13}C NMR spectra and HRMS of synthetic compounds.

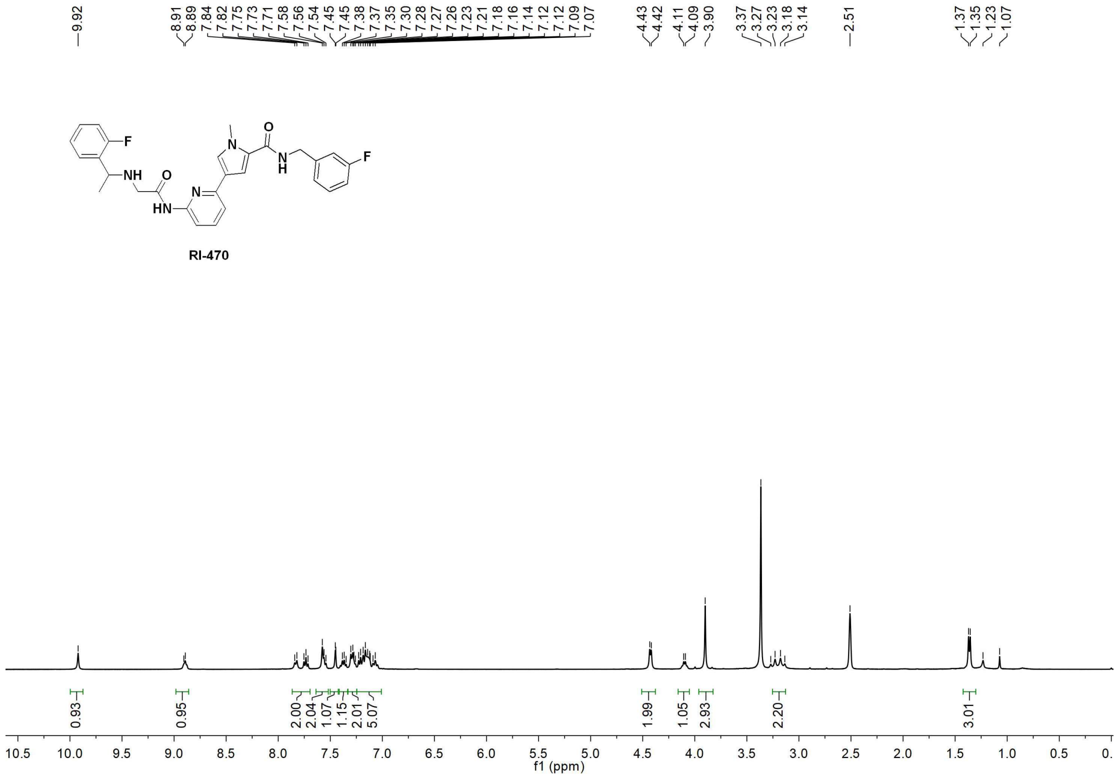

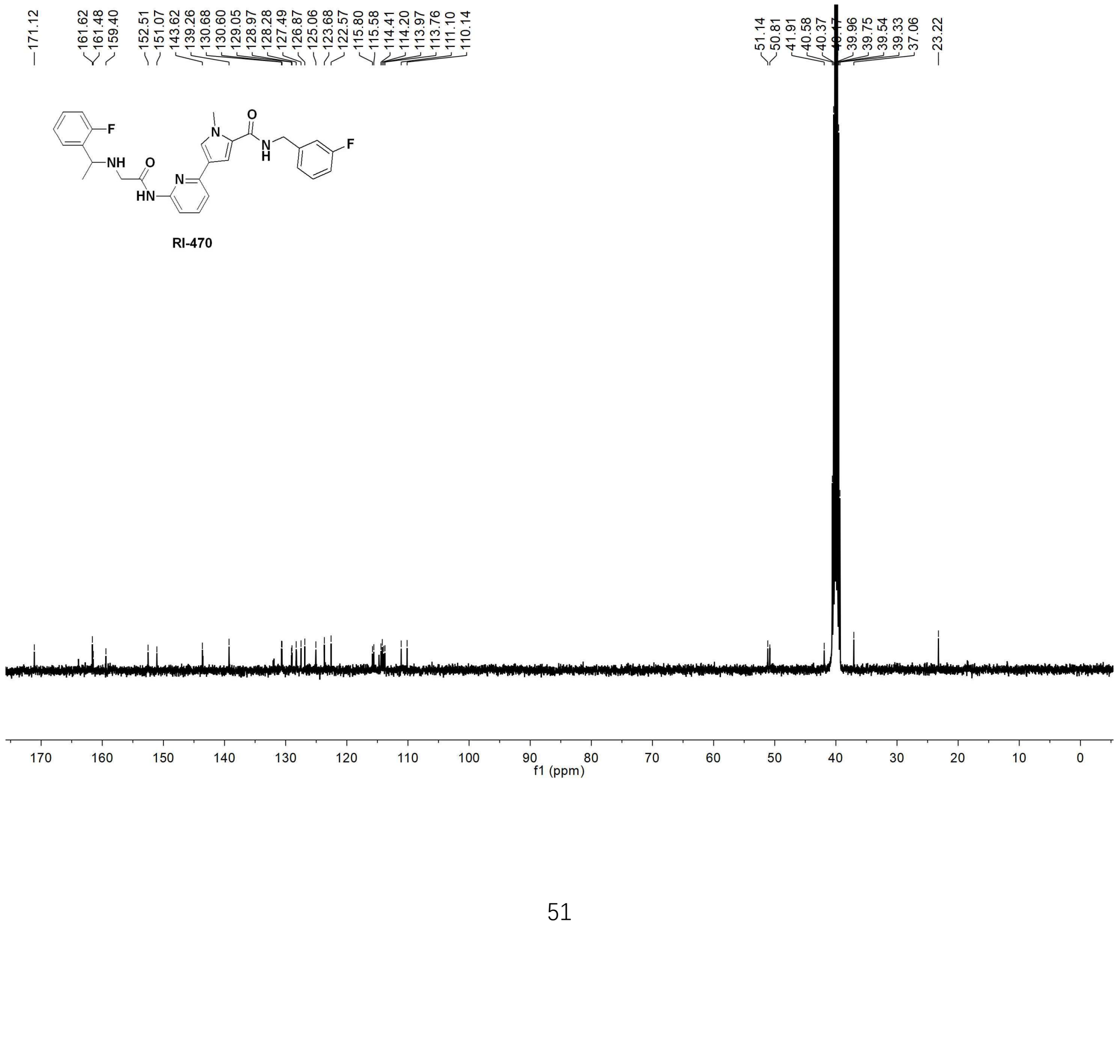

1D #1-15 RT: 0.00-0.07 AV: 15 NL: 1.06E8
T: FTMS + p ESI Full ms [150.0000-2000.0000]

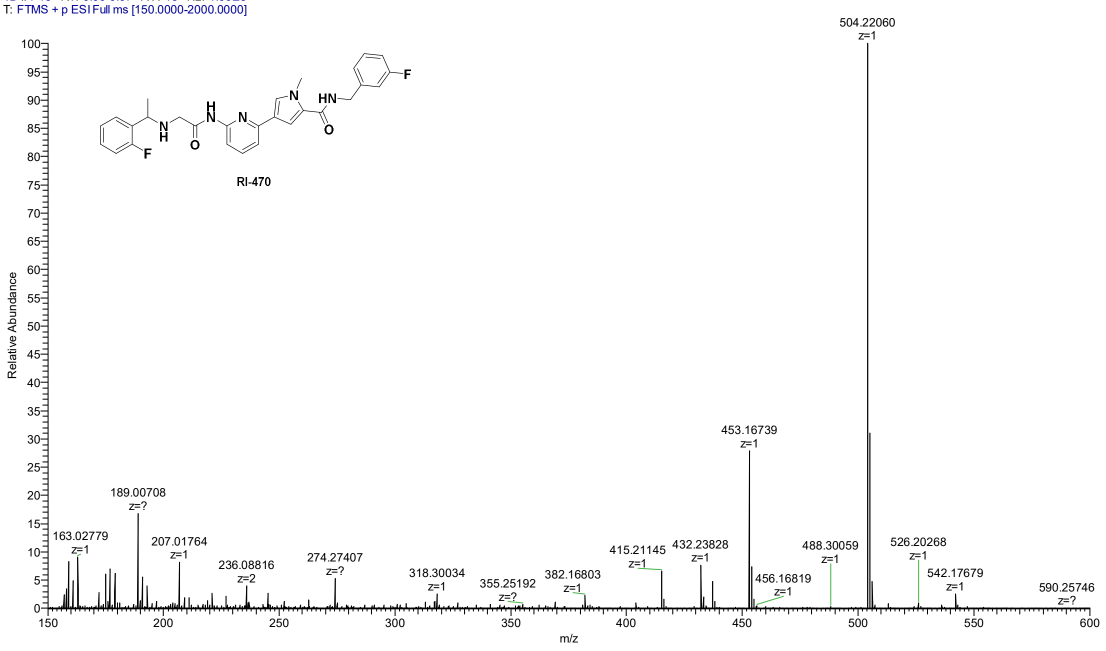

RI-470

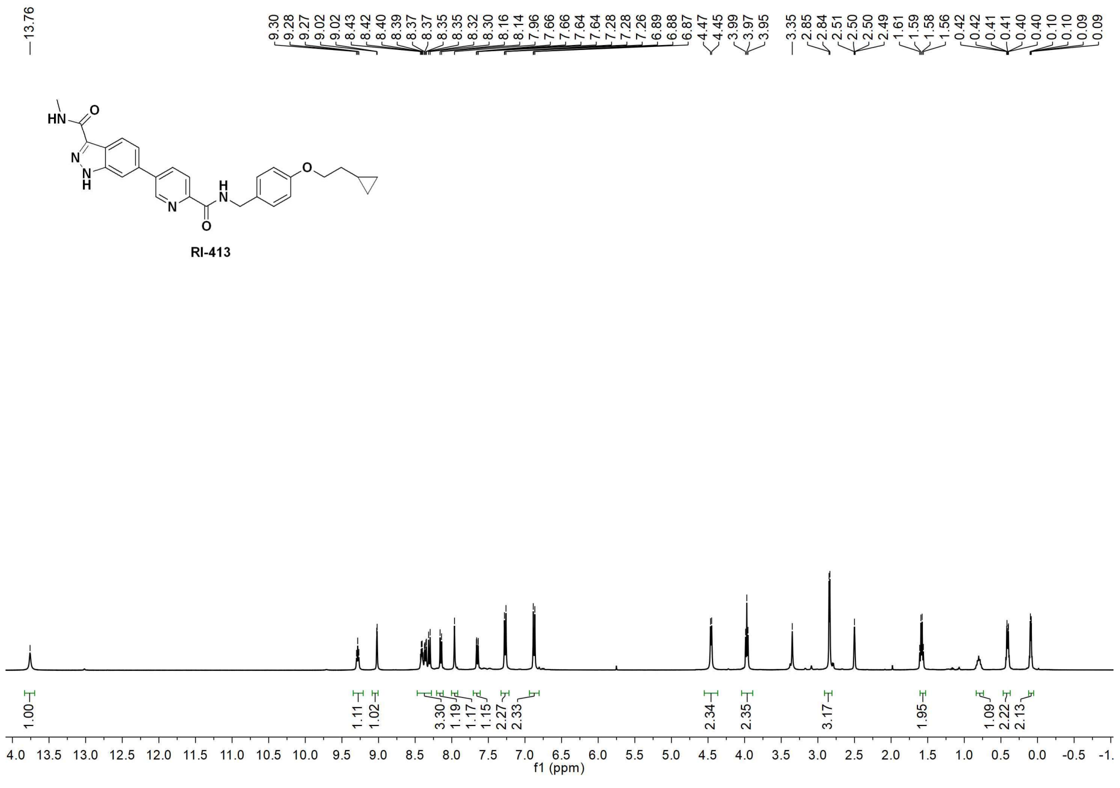

RI-413

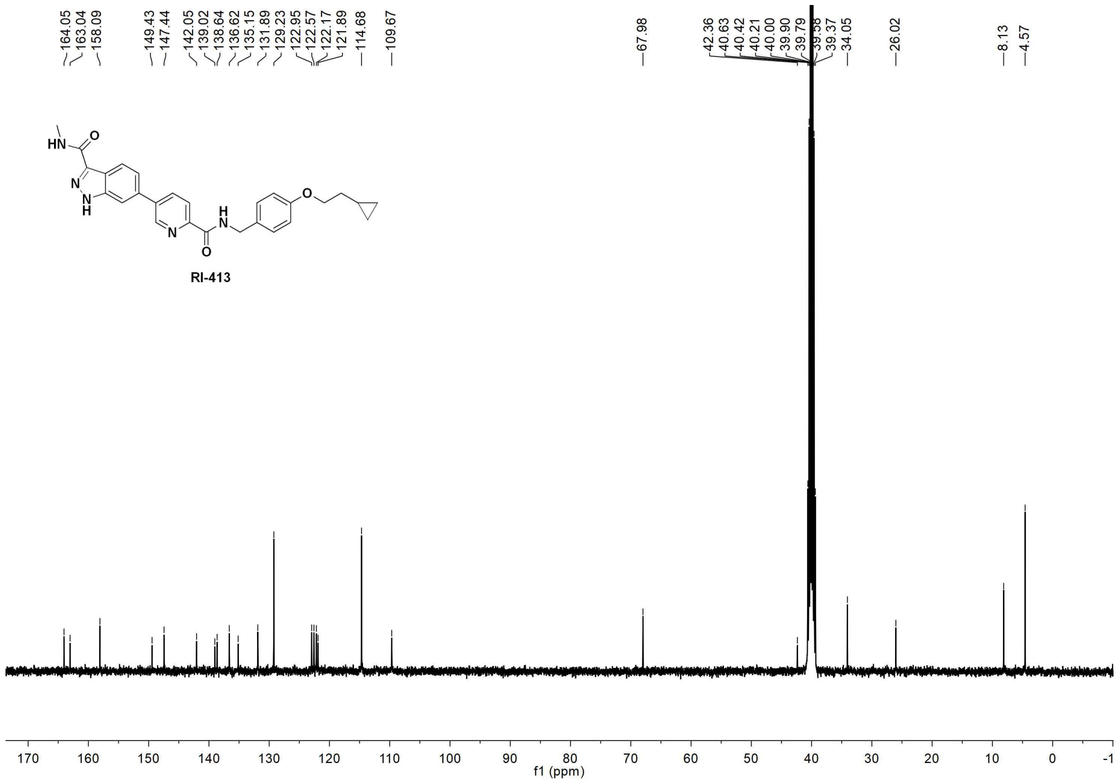
164.05
163.04
158.09
149.43
147.44
142.05
139.02
138.64
136.62
135.15
131.89
129.23
122.95
122.57
122.17
121.89
114.68
109.67
67.98
42.36
40.63
40.42
40.21
40.00
39.90
39.79
39.58
39.37
34.05
26.02
8.13
4.57
RI-413
f1 (ppm)

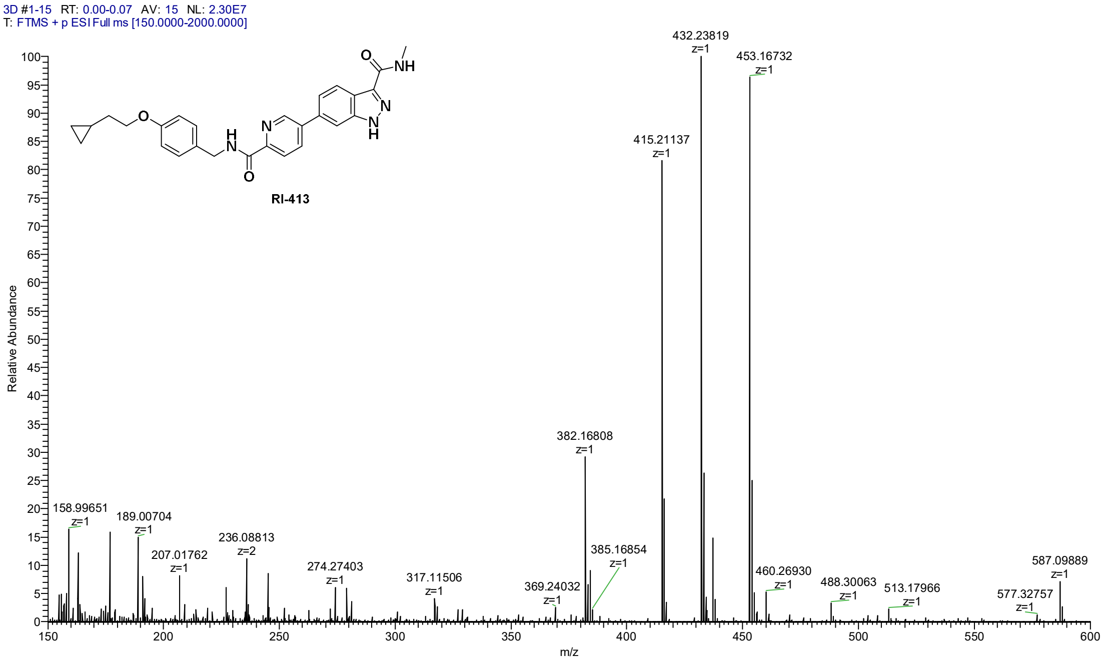
3D #1-15 RT: 0.00-0.07 AV: 15 NL: 2.30E7
T: FTMS + p ESI Full ms [150.0000-2000.0000]
RI-413
Relative Abundance
m/z
432.23819 z=1
453.16732 z=1
415.21137 z=1
382.16808 z=1
385.16854 z=1
158.99651 z=1
189.00704 z=1
207.01762 z=1
236.08813 z=2
274.27403 z=1
317.11506 z=1
369.24032 z=1
460.26930 z=1
488.30063 z=1
513.17966 z=1
577.32757 z=1
587.09889 z=1

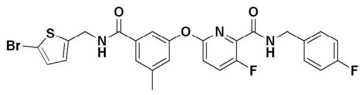

Br
S
O
N
H
O
N
O
N
H
F
F

RI-539

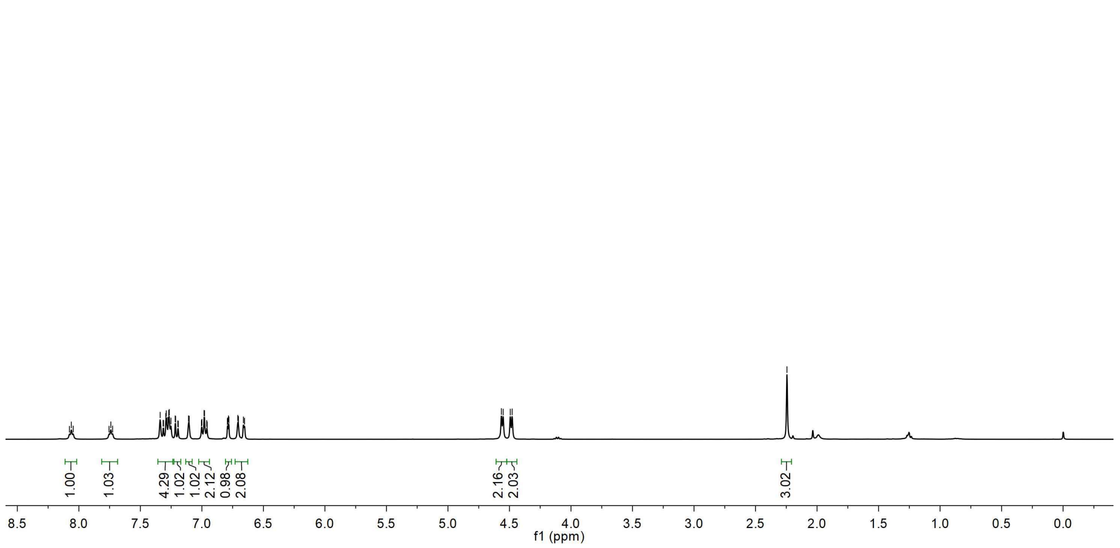

8.08
8.06
8.05
7.75
7.74
7.73
7.34
7.32
7.31
7.29
7.29
7.28
7.27
7.27
7.27
7.25
7.22
7.21
7.20
7.19
7.11
7.10
7.00
7.00
6.98
6.98
6.96
6.96
6.79
6.79
6.78
6.71
6.70
6.66
6.65
4.57
4.55
4.49
4.48
2.24
1.00
1.03
4.29
1.02
1.02
2.12
0.98
2.08
2.16
2.03
3.02
8.5
8.0
7.5
7.0
6.5
6.0
5.5
5.0
4.5
4.0
3.5
3.0
2.5
2.0
1.5
1.0
0.5
0.0
f1 (ppm)

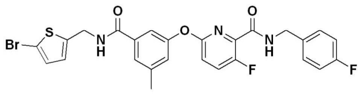

Br
S
O
N
H
O
N
O
N
H
F
F

RI-539

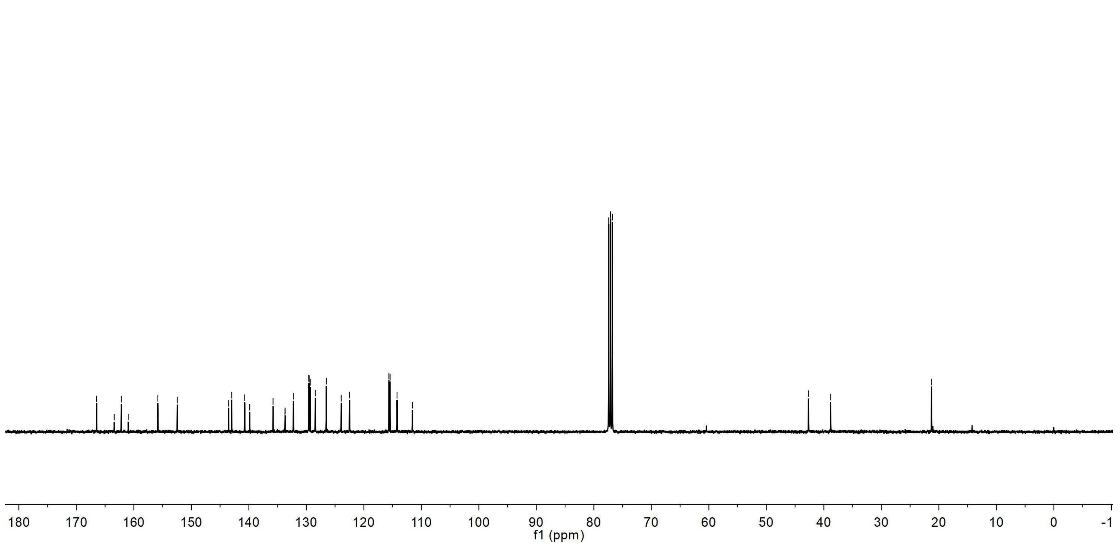

166.47
163.41
162.18
160.97
155.83
152.45
143.50
142.98
140.72
139.85
135.79
133.72
133.69
132.25
129.58
129.50
129.31
128.44
126.53
123.94
122.49
115.65
115.43
114.23
111.56
77.41
77.09
76.77
42.67
38.82
21.28
180
170
160
150
140
130
120
110
100
90
80
70
60
50
40
30
20
10
0
-1
f1 (ppm)

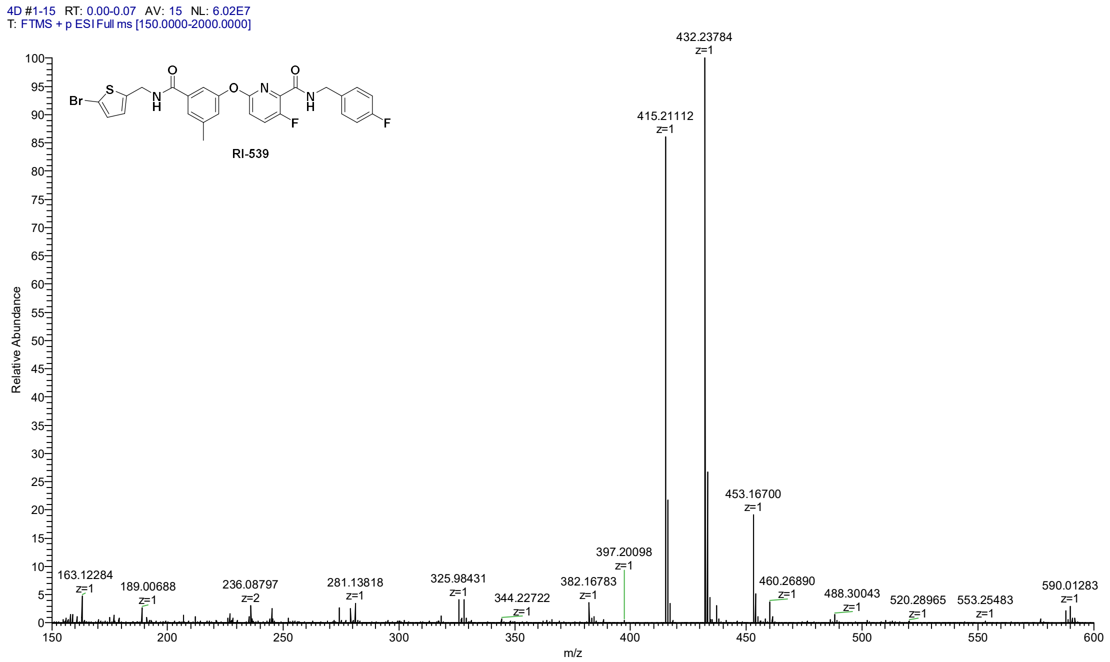
4D #1-15 RT: 0.00-0.07 AV: 15 NL: 6.02E7
T: FTMS + p ESI Full ms [150.0000-2000.0000]
RI-539
432.23784 z=1
415.21112 z=1
453.16700 z=1
397.20098 z=1
382.16783 z=1
325.98431 z=1
344.22722 z=1
281.13818 z=1
236.08797 z=2
189.00688 z=1
163.12284 z=1
460.26890 z=1
488.30043 z=1
520.28965 z=1
553.25483 z=1
590.01283 z=1
Relative Abundance
m/z

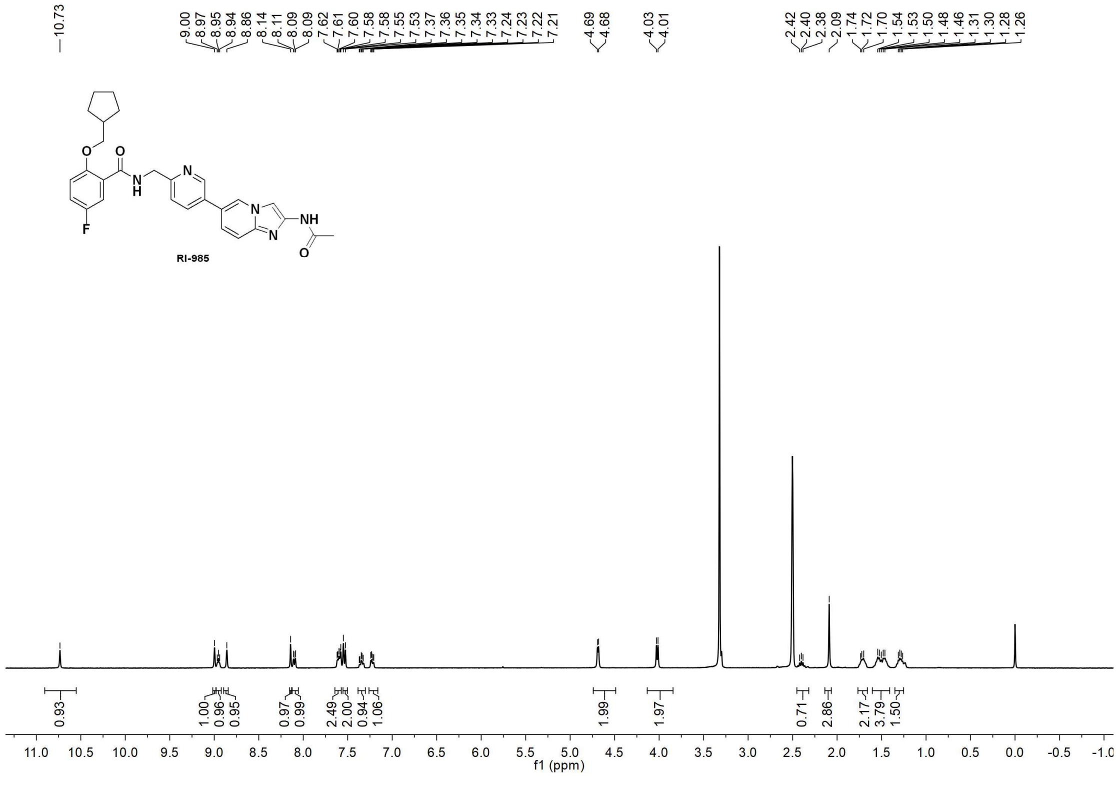
RI-985
10.73
9.00 8.97 8.95 8.94 8.86 8.14 8.11 8.09 8.09 7.62 7.61 7.60 7.58 7.58 7.55 7.53 7.37 7.36 7.35 7.34 7.33 7.24 7.23 7.22 7.21
4.69 4.68
4.03 4.01
2.42 2.40 2.38 2.09 1.74 1.72 1.70 1.54 1.53 1.50 1.48 1.46 1.31 1.30 1.28 1.26
0.93 1.00 0.96 0.95 0.97 0.99 2.49 2.00 0.94 1.06 1.99 1.97 0.71 2.86 2.17 3.79 1.50
f1 (ppm)

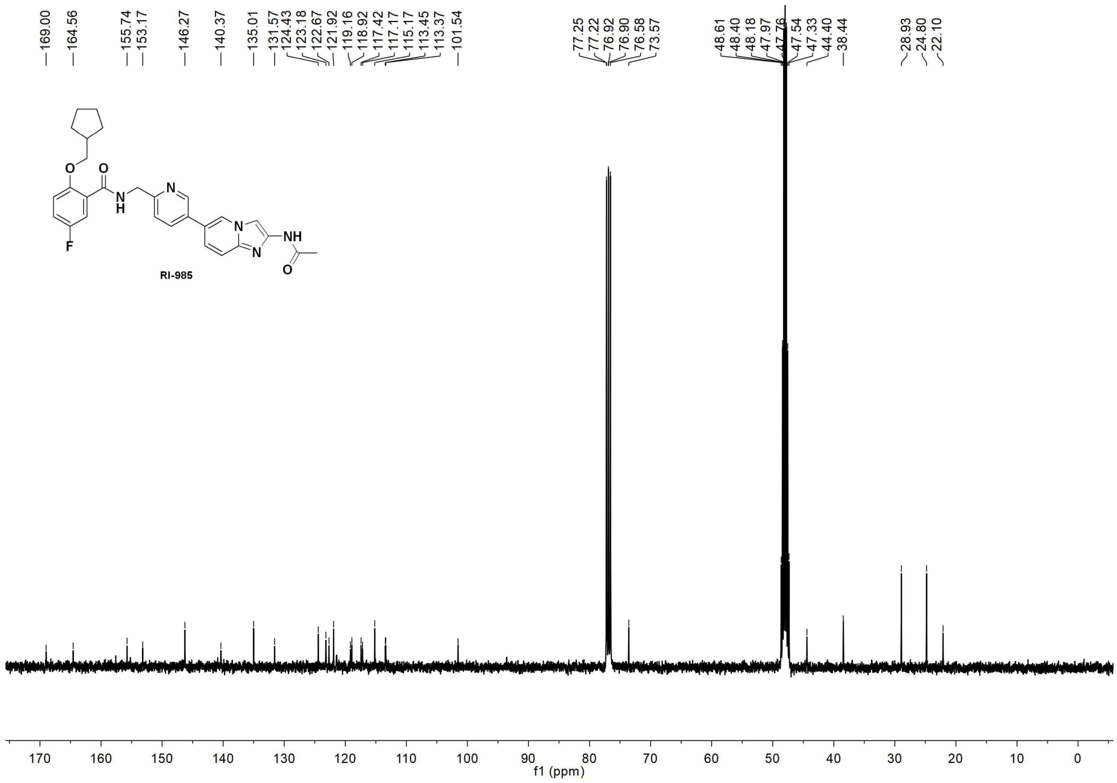
169.00
164.56
155.74
153.17
146.27
140.37
135.01
131.57
124.43
123.18
122.67
121.92
119.16
118.92
117.42
117.17
115.17
113.45
113.37
101.54
77.25
77.22
76.92
76.90
76.58
73.57
48.61
48.40
48.18
47.97
47.76
47.54
47.33
44.40
38.44
28.93
24.80
22.10
RI-985
f1 (ppm)

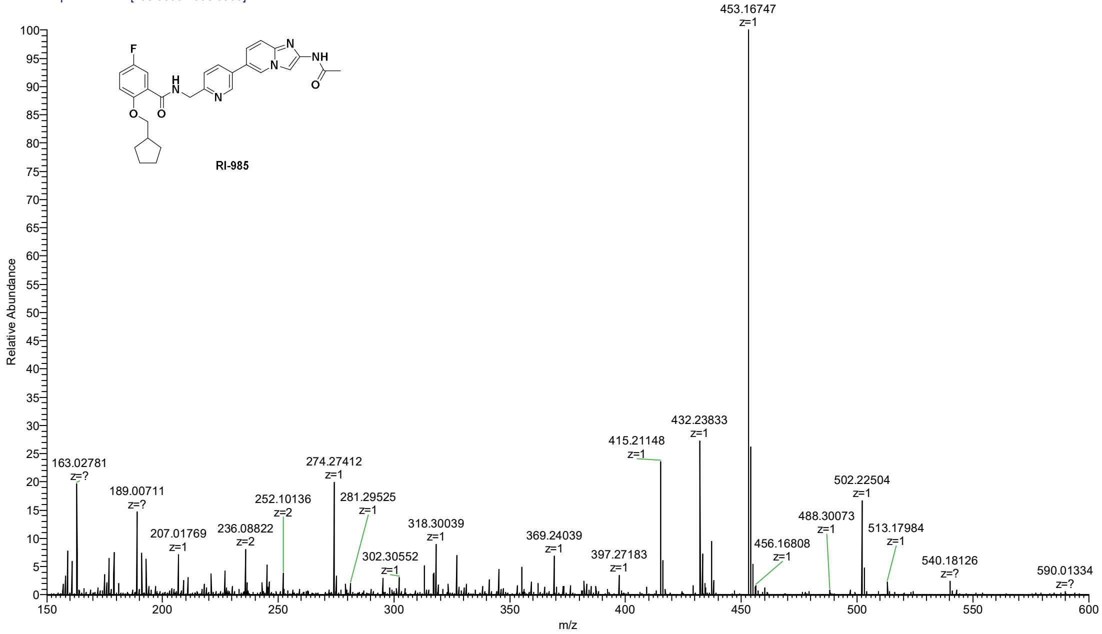
8 #1-15 RT: 0.00-0.07 AV: 15 NL: 6.59E7
T: FTMS + p ESI Full ms [150.0000-2000.0000]
RI-985
Relative Abundance
m/z
453.16747 z=1
432.23833 z=1
415.21148 z=1
502.22504 z=1
488.30073 z=1
513.17984 z=1
456.16808 z=1
540.18126 z=?
590.01334 z=?
163.02781 z=?
189.00711 z=?
207.01769 z=1
236.08822 z=2
252.10136 z=2
274.27412 z=1
281.29525 z=1
302.30552 z=1
318.30039 z=1
369.24039 z=1
397.27183 z=1

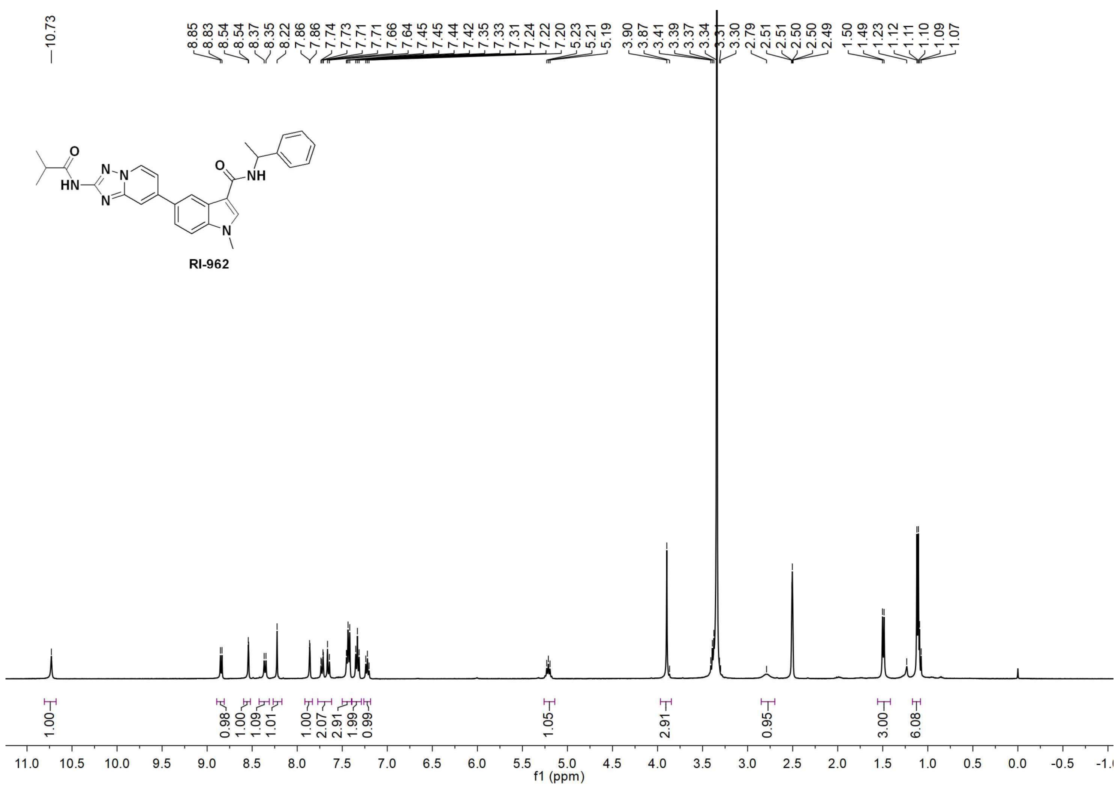
10.73
8.85 8.83 8.54 8.54 8.37 8.35 8.22 7.86 7.86 7.74 7.73 7.71 7.71 7.66 7.64 7.45 7.45 7.44 7.42 7.35 7.33 7.31 7.24 7.22 7.20 5.23 5.21 5.19
3.90 3.87 3.41 3.39 3.37 3.34 3.31 3.30 2.79 2.51 2.51 2.50 2.50 2.49 1.50 1.49 1.23 1.12 1.11 1.10 1.09 1.07
O
HN
N
N
NH
RI-962
1.00 0.98 1.00 1.09 1.01 1.00 2.07 2.91 1.99 0.99 1.05 2.91 0.95 3.00 6.08
f1 (ppm)

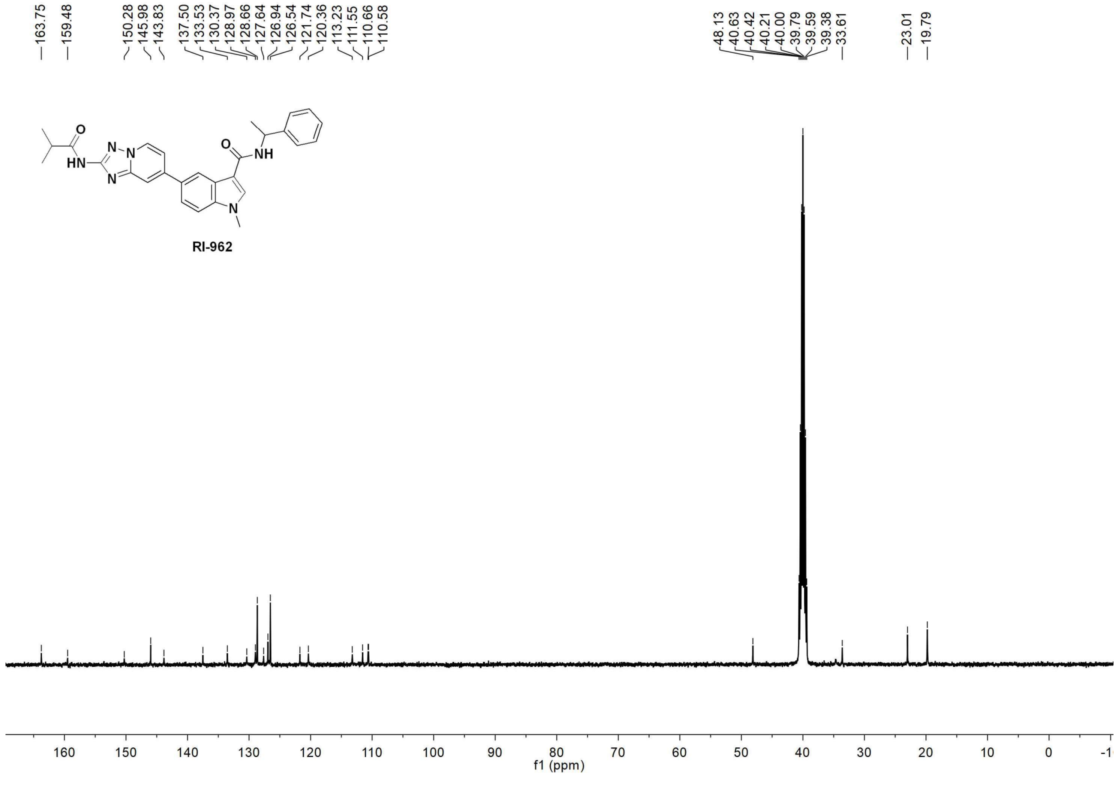
163.75 159.48 150.28 145.98 143.83 137.50 133.53 130.37 128.97 128.66 127.64 126.94 126.54 121.74 120.36 113.23 111.55 110.66 110.58
48.13 40.63 40.42 40.21 40.00 39.79 39.59 39.38 33.61 23.01 19.79
O
HN
N
N
NH
RI-962
f1 (ppm)

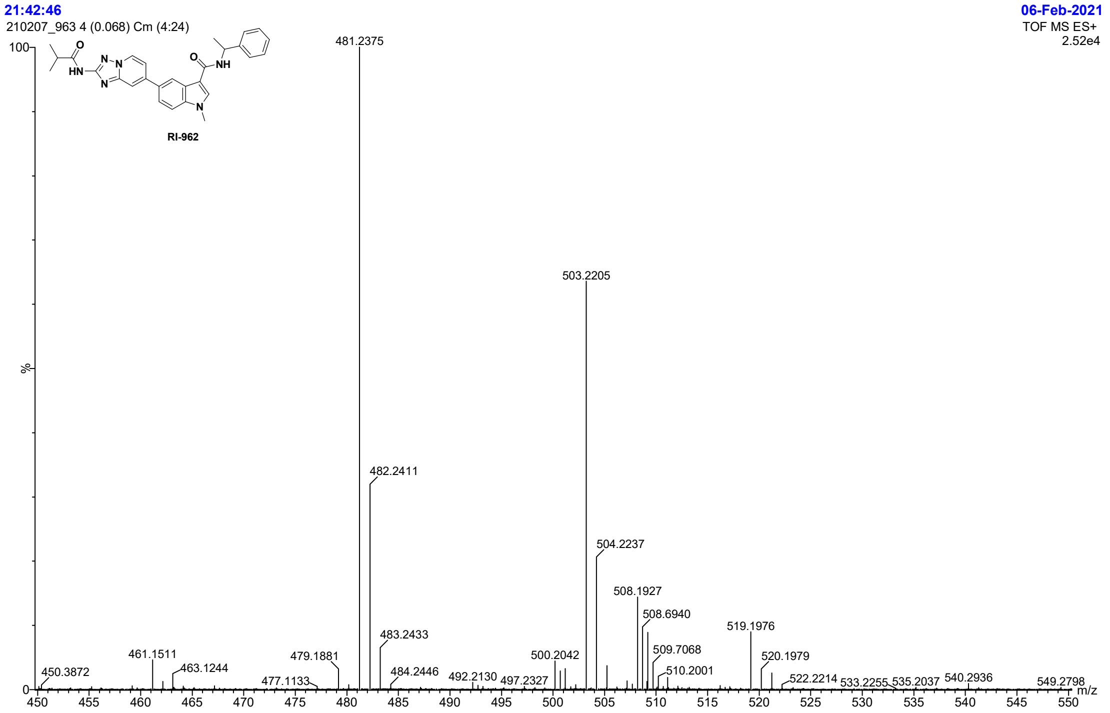

21:42:46
210207_963 4 (0.068) Cm (4:24)
06-Feb-2021
TOF MS ES+
2.52e4
RI-962
481.2375
503.2205
482.2411
504.2237
508.1927
508.6940
519.1976
483.2433
509.7068
520.1979
461.1511
479.1881
500.2042
463.1244
510.2001
450.3872
484.2446
492.2130
497.2327
477.1133
522.2214
533.2255
535.2037
540.2936
549.2798
m/z
%

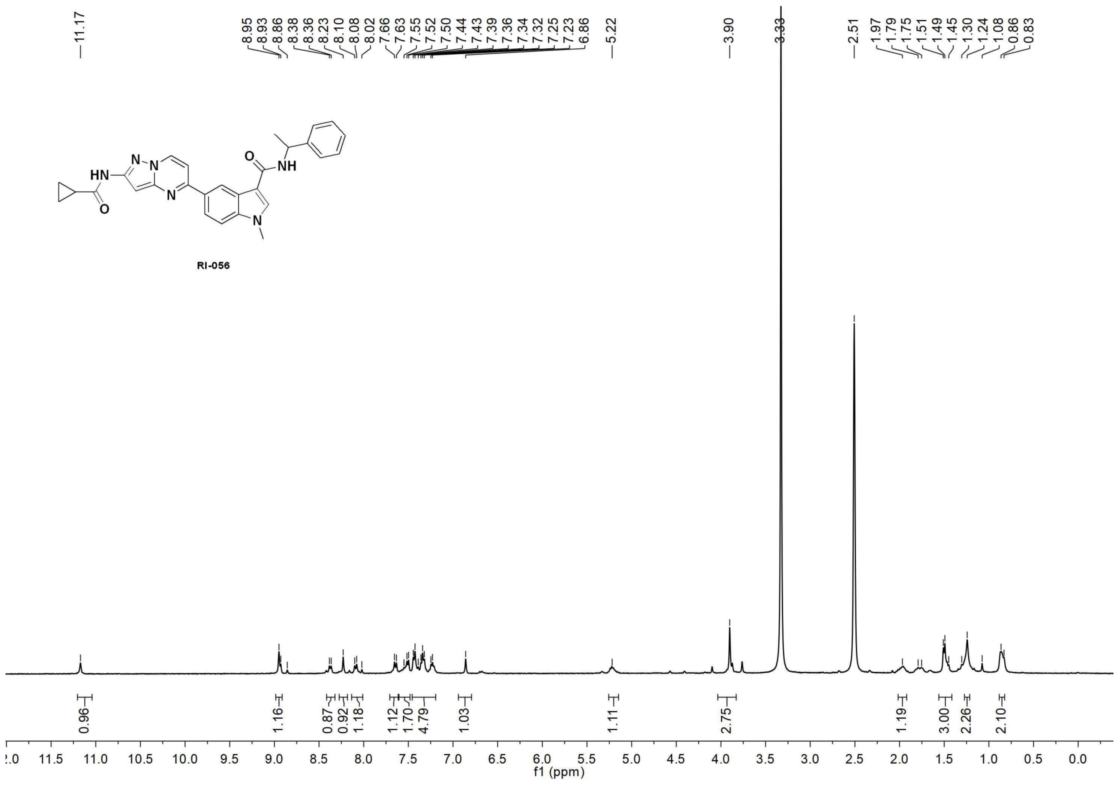

RI-056
11.17
8.95 8.93 8.86 8.38 8.36 8.23 8.10 8.08 8.02 7.66 7.63 7.55 7.52 7.50 7.44 7.43 7.39 7.36 7.34 7.32 7.25 7.23 6.86
5.22
3.90
3.33
2.51 1.97 1.79 1.75 1.51 1.49 1.45 1.30 1.24 1.08 0.86 0.83
0.96 1.16 0.87 0.92 1.18 1.12 1.70 4.79 1.03 1.11 2.75 1.19 3.00 2.26 2.10
f1 (ppm)

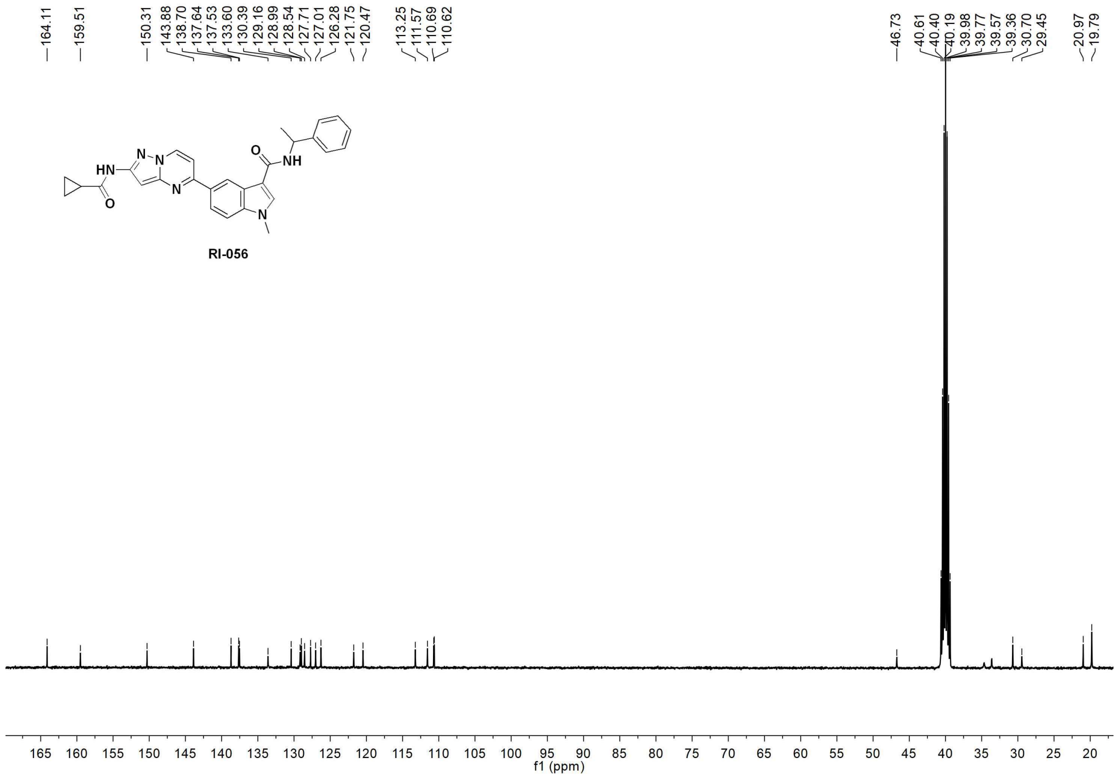
164.11
159.51
150.31
143.88
138.70
137.64
137.53
133.60
130.39
129.16
128.99
128.54
127.71
127.01
126.28
121.75
120.47
113.25
111.57
110.69
110.62
46.73
40.61
40.40
40.19
39.98
39.77
39.57
39.36
30.70
29.45
20.97
19.79
HN
N
N
N
O
O
NH
N
RI-056
f1 (ppm)

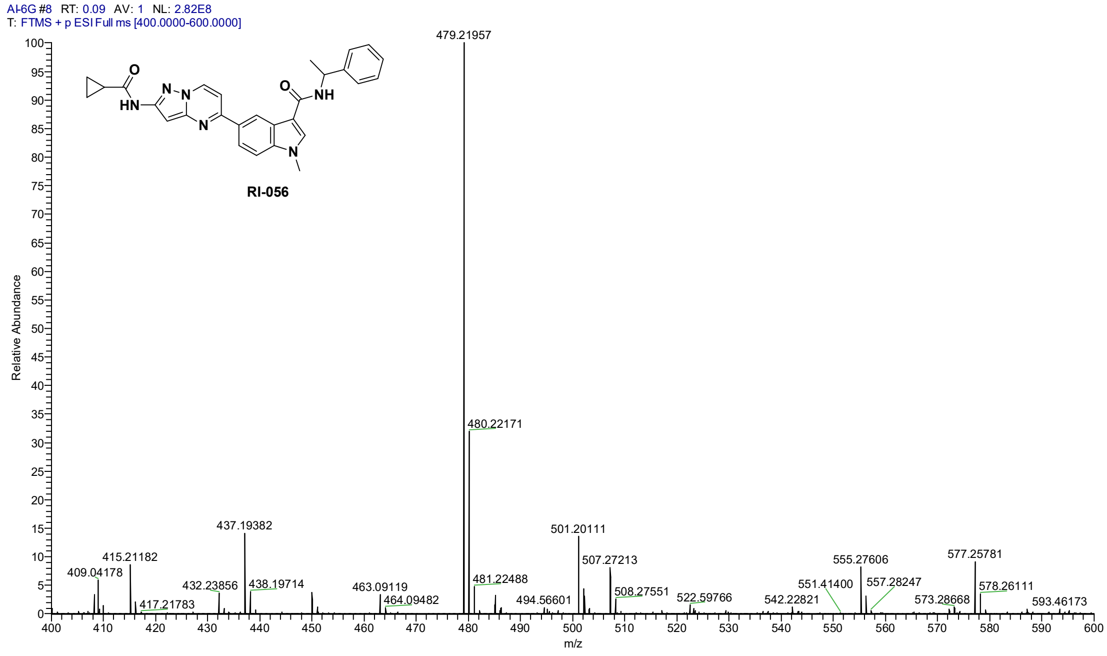
AI-6G #8 RT: 0.09 AV: 1 NL: 2.82E8
T: FTMS + p ESI Full ms [400.0000-600.0000]
479.21957
480.22171
437.19382
415.21182
409.04178
432.23856
438.19714
417.21783
463.09119
464.09482
481.22488
494.56601
501.20111
507.27213
508.27551
522.59766
542.22821
551.41400
555.27606
557.28247
573.28668
577.25781
578.26111
593.46173
RI-056
Relative Abundance
m/z

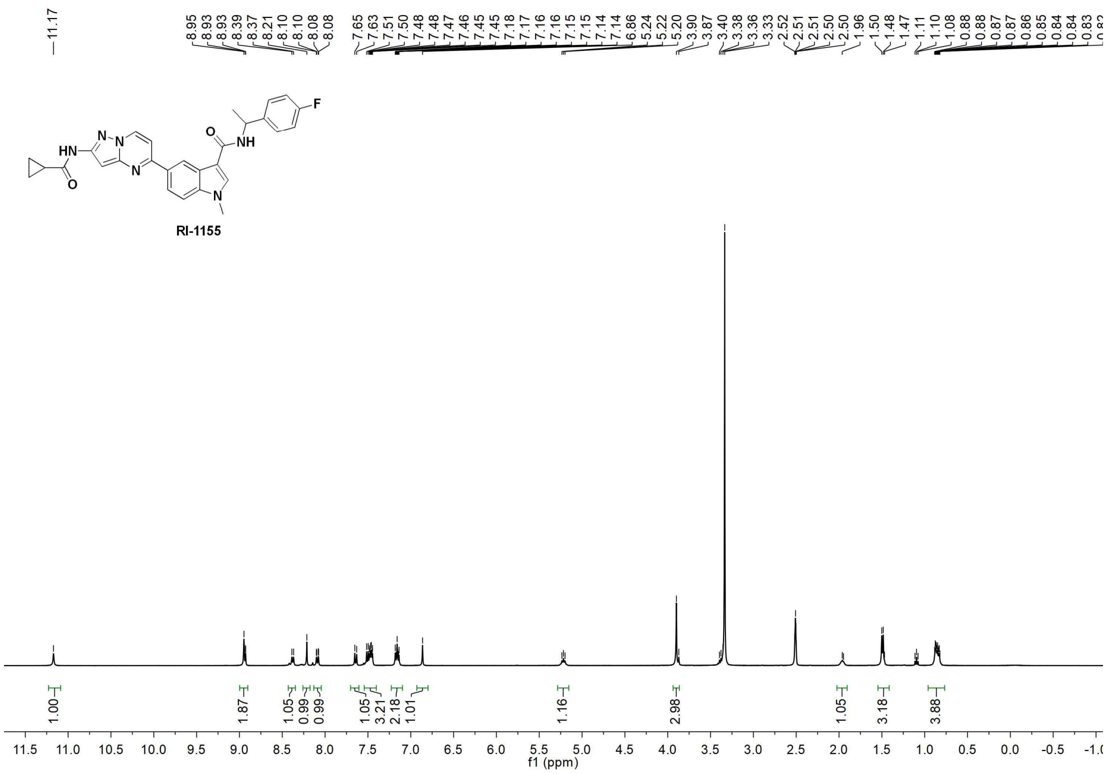

11.17
8.95 8.93 8.93 8.39 8.37 8.21 8.10 8.10 8.08 8.08
7.65 7.63 7.51 7.50 7.48 7.48 7.47 7.46 7.45 7.45 7.18 7.17 7.16 7.16 7.15 7.15 7.14 6.86 5.24 5.22 5.20 3.90 3.87 3.40 3.38 3.36 3.33 2.52 2.51 2.51 2.50 2.50 1.96 1.50 1.48 1.47 1.11 1.10 1.08 0.88 0.88 0.87 0.87 0.86 0.85 0.84 0.84 0.83
RI-1155
1.00
1.87
1.05 0.99 0.99
1.05 3.21 2.18 1.01
1.16
2.98
1.05
3.18
3.88
f1 (ppm)

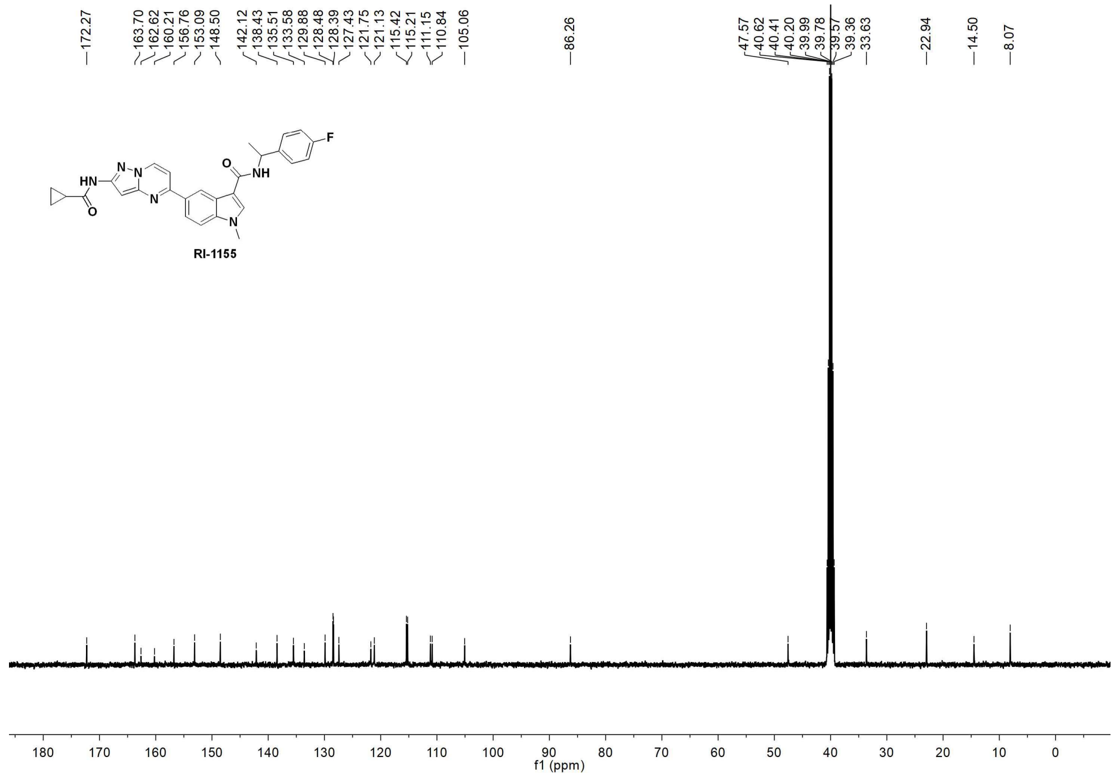

172.27
163.70 162.62 160.21 156.76 153.09 148.50
142.12 138.43 135.51 133.58 129.88 128.48 128.39 127.43 121.75 121.13 115.42 115.21 111.15 110.84
105.06
86.26
47.57 40.62 40.41 40.20 39.99 39.78 39.57 39.36 33.63
22.94
14.50
8.07
RI-1155
f1 (ppm)

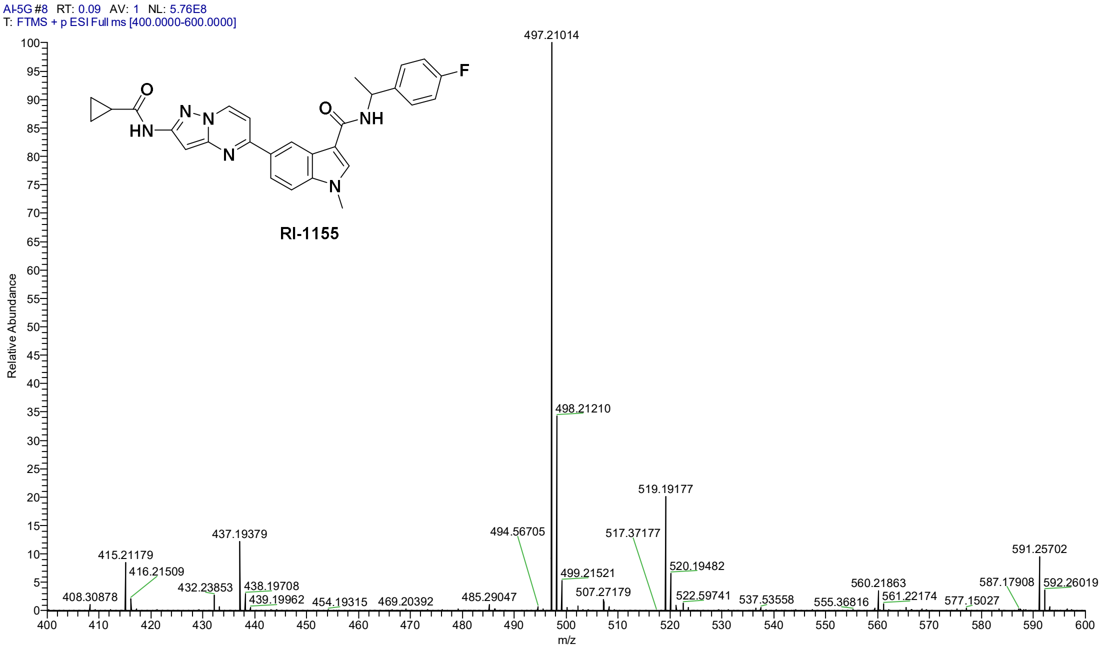
AI-5G #8 RT: 0.09 AV: 1 NL: 5.76E8
T: FTMS + p ESI Full ms [400.0000-600.0000]
RI-1155
Relative Abundance
m/z
497.21014
498.21210
519.19177
437.19379
415.21179
416.21509
591.25702
592.26019
494.56705
517.37177
520.19482
499.21521
507.27179
560.21863
587.17908
432.23853
438.19708
439.19962
408.30878
454.19315
469.20392
485.29047
522.59741
537.53558
555.36816
561.22174
577.15027

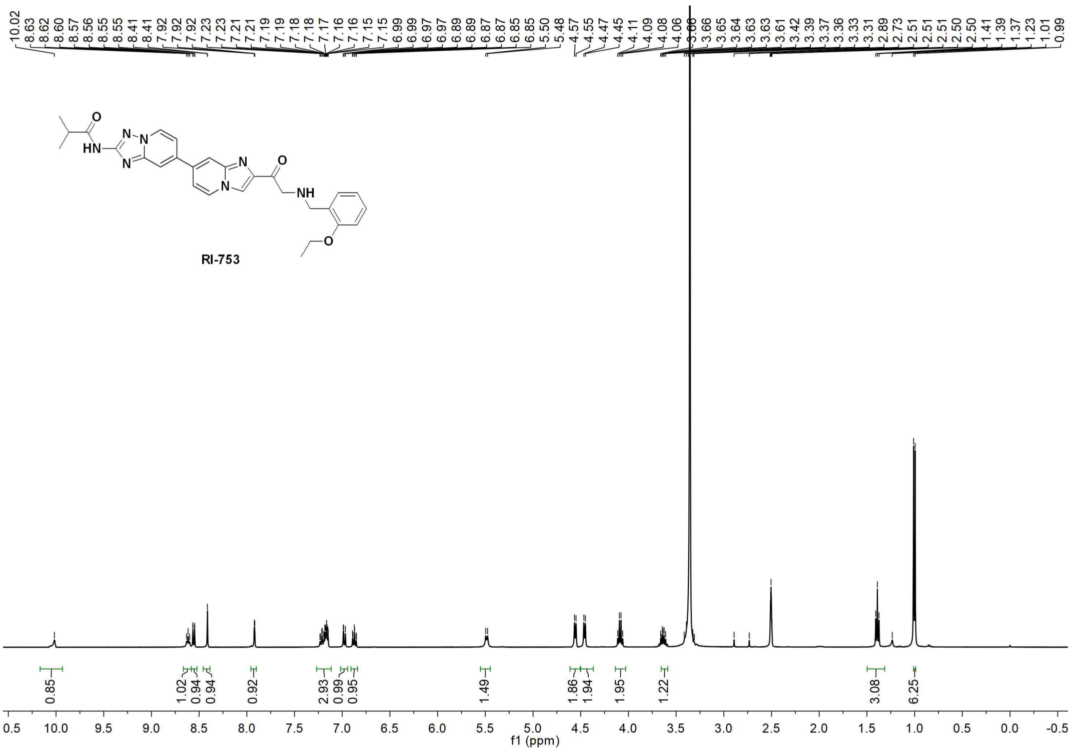
RI-753
f1 (ppm)
10.02 8.63 8.62 8.60 8.57 8.56 8.55 8.55 8.41 8.41 7.92 7.92 7.92 7.23 7.23 7.21 7.21 7.19 7.18 7.18 7.17 7.16 7.16 7.15 6.99 6.99 6.97 6.97 6.89 6.89 6.87 6.85 5.50 5.48 4.57 4.55 4.47 4.45 4.11 4.09 4.08 4.06 3.66 3.66 3.65 3.64 3.63 3.63 3.61 3.42 3.39 3.37 3.36 3.33 3.31 2.89 2.73 2.51 2.51 2.50 2.50 1.41 1.39 1.37 1.23 1.01 0.99
0.85 1.02 0.94 0.94 0.92 2.93 0.99 0.95 1.49 1.86 1.94 1.95 1.22 3.08 6.25

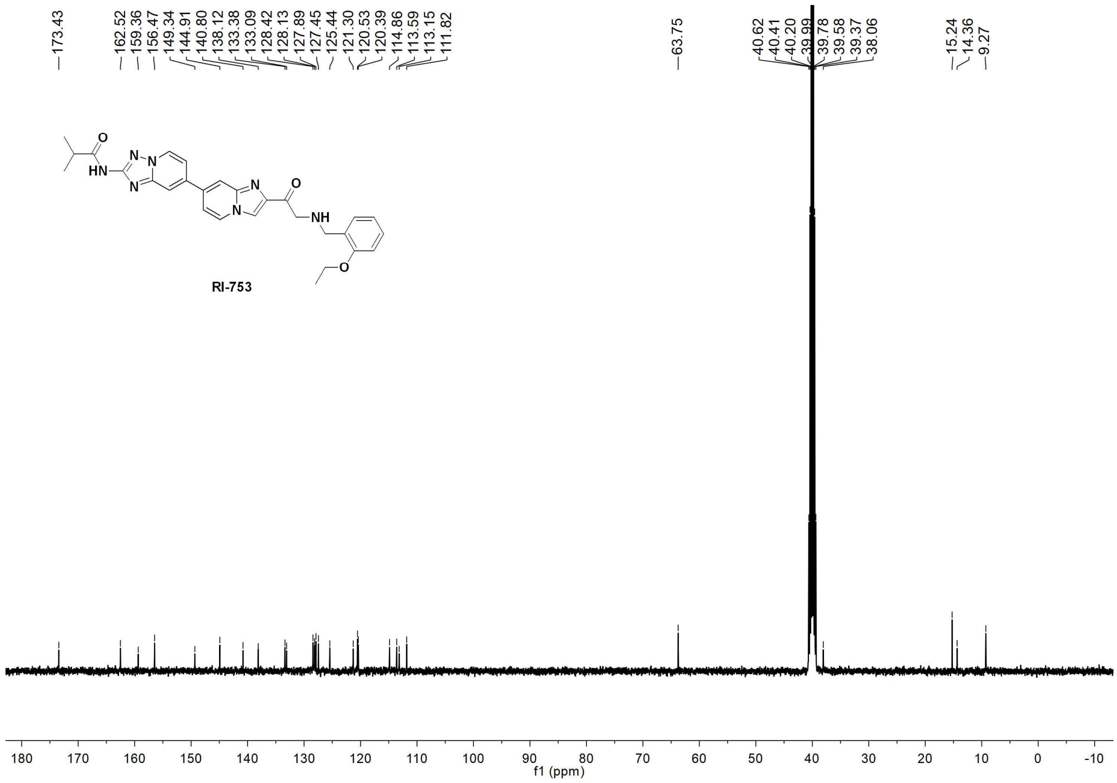
173.43
162.52
159.36
156.47
149.34
144.91
140.80
138.12
133.38
133.09
128.42
128.13
127.89
127.45
125.44
121.30
120.53
120.39
114.86
113.59
113.15
111.82
63.75
40.62
40.41
40.20
39.99
39.78
39.58
39.37
38.06
15.24
14.36
9.27
RI-753
f1 (ppm)

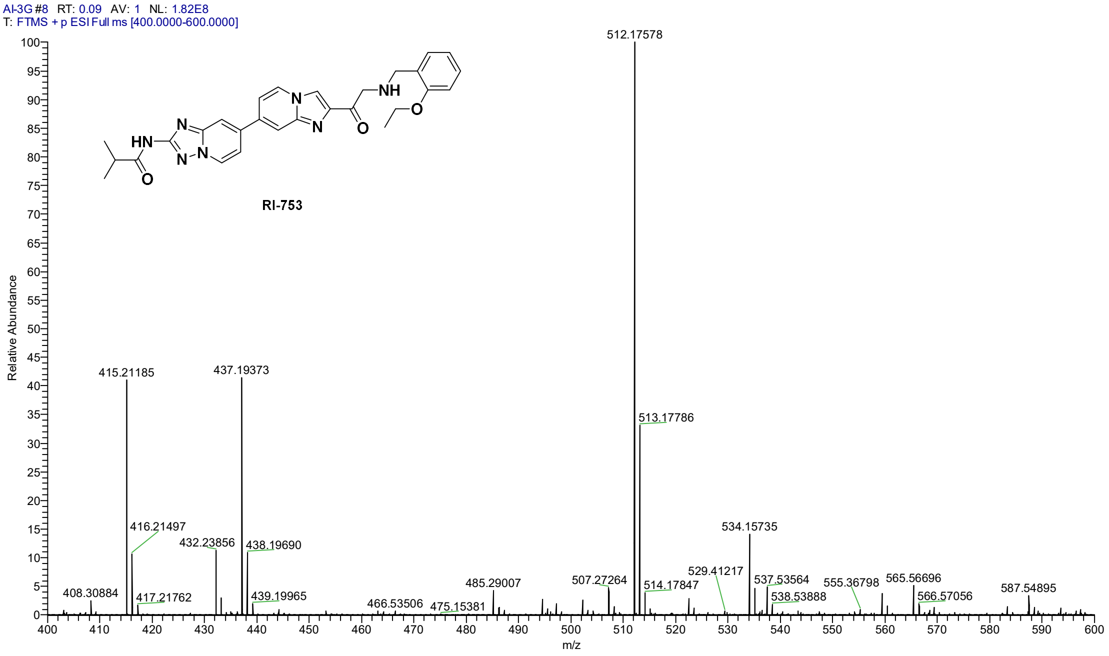
AI-3G #8 RT: 0.09 AV: 1 NL: 1.82E8
T: FTMS + p ESI Full ms [400.0000-600.0000]
RI-753
Relative Abundance
m/z
512.17578
415.21185
437.19373
513.17786
534.15735
416.21497
432.23856
438.19690
408.30884
417.21762
439.19965
466.53506
475.15381
485.29007
507.27264
514.17847
529.41217
537.53564
538.53888
555.36798
565.56696
566.57056
587.54895

Supplementary References

1. Ren, Y. et al. Discovery of a highly potent, selective, and metabolically stable inhibitor of receptor-interacting protein 1 (RIP1) for the treatment of systemic inflammatory response syndrome. *J. Med. Chem.* **60**, 972–986 (2017).
2. Karaman, M. W. et al. A quantitative analysis of kinase inhibitor selectivity. *Nat. Biotechnol.* **26**, 127–132 (2008).
3. Davis, M. I. et al. Comprehensive analysis of kinase inhibitor selectivity. *Nat. Biotechnol.* **29**, 1046–1051 (2011).
4. Zarrinkar, P. P. et al. AC220 is a uniquely potent and selective inhibitor of FLT3 for the treatment of acute myeloid leukemia (AML), *Blood* **114**, 2984–2992 (2009).
5. Harris, P. A. et al. Discovery of small molecule RIP1 kinase inhibitors for the treatment of pathologies associated with necroptosis. *ACS Med. Chem. Lett.* **4**, 1238–1243 (2013).
6. Harris, P. A. et al. Discovery of a first-in-class receptor interacting protein 1 (RIP1) kinase specific clinical candidate (GSK2982772) for the treatment of inflammatory diseases. *J. Med. Chem.* **60**, 1247–1261 (2017).
7. Yoshikawa, M. et al. Discovery of 7-oxo-2,4,5,7-tetrahydro-6*H*-pyrazolo[3,4-*c*]pyridine derivatives as potent, orally available, and brain-penetrating receptor interacting protein 1 (RIP1) kinase inhibitors: Analysis of structure-kinetic relationships. *J. Med. Chem.* **61**, 2384–2409 (2018).
8. Harris, P. A. et al. DNA-encoded library screening identifies benzo[*b*][1,4]oxazepin-4-ones as highly potent and monoselective receptor interacting protein 1 kinase inhibitors. *J. Med. Chem.* **59**, 2163–2178 (2016).
9. Munoz, L. Non-kinase targets of protein kinase inhibitors. *Nat. Rev. Drug Discov.* **16**, 424–440 (2017).
10. Bengio, Y., Ducharme, R., Vincent, P. & Janvin, C. A neural probabilistic language model. *J. Mach. Learn. Res.* **3**, 1137–1155 (2003).
11. Collobert, R. & Weston, J. A unified architecture for natural language processing: Deep neural networks with multitask learning. In *Proceedings of the 25th international conference on Machine learning*. 160–167 (2008).
12. Cireşan, D. C., Meier, U. & Schmidhuber, J. Transfer learning for Latin and Chinese characters with deep neural networks. In *The 2012 International Joint Conference on Neural Networks (IJCNN)*, 1−6 (2012).
13. Moret, M., Friedrich, L., Grisoni, F., Merk, D. & Schneider, G. Generative molecular design in low data regimes. *Nat. Mach. Intell.* **2**, 171-180 (2020).
14. Segler, M. H. S., Kogej, T., Tyrchan, C. & Waller, M. P. Generating focused molecule libraries for drug discovery with recurrent neural networks. *ACS Cent. Sci.* **4**, 120–131 (2018).
15. Gupta, A. et al. Generative recurrent networks for *de novo* drug design. *Mol. Inform*. **37**, 1700111 (2018).
16. Merk, D., Friedrich, L., Grisoni, F. & Schneider, G. *De novo* design of bioactive small molecules by artificial intelligence. *Mol. Inform.* **37**, 1700153 (2018).
17. Cai, C. et al. Transfer learning for drug discovery. *J. Med. Chem.* **63**, 8683–8694 (2020).
18. An, G. The effects of adding noise during backpropagation training on a generalization performance. *Neural Comput.* **8**, 643–674 (1996).

19. Vincent, P., Larochelle, H., Lajoie, I., Bengio, Y. & Manzagol, P.-A. Stacked denoising autoencoders: Learning useful representations in a deep network with a local denoising criterion. *J. Mach. Learn. Res.* **11**, 3371–3408 (2010).
20. Poole, B., Sohl-Dicksteiny, J. & Ganguliy, S. Analyzing noise in autoencoders and deep networks. Preprint at https://arxiv.org/abs/1406.1831 (2014).
21. Bishop, C. M. Training with noise is equivalent to Tikhonov regularization. *Neural Comput.* **7**, 108–116 (1995).
22. Reed, R. & Marks II, R. J. *Neural Smithing: Supervised Learning in Feedforward Artificial Neural Networks*. (MIT Press, 1999).
23. Rifai, S., Glorot, X., Bengio, Y. & Vincent P. Adding noise to the input of a model trained with a regularized objective. Preprint at https://arxiv.org/abs/1104.3250 (2018).
24. Webb, A. R. Functional approximation by feed-forward networks: A least-squares approach to generalization. *IEEE Transactions on Neural Networks and Learning Systems* 363–371 (1994).
25. Chapelle, O., Schölkopf, B. & Zien, A. *Semi-Supervised Learning*. (The MIT Press, 2006).
26. Sietsma, J. & Dow, R. J. F. Creating artificial neural networks that generalize. *Neural Netw.* **4**, 67–79 (1991).
27. Harel, S. & Radinsky, K. Prototype-based compound discovery using deep generative models. *Mol. Pharm.* **15**, 4406–4416 (2018).
28. Gómez-Bombarelli, R. et al. Automatic chemical design using a data-driven continuous representation of molecules. *ACS Cent. Sci.* **4**, 268–276 (2018).
29. Berthelot, D., Raffel, C., Roy, A. & Goodfellow, I. Understanding and improving interpolation in autoencoders via an adversarial regularizer. Preprint at https://arxiv.org/abs/1807.07543 (2018).
30. White, T. Sampling generative networks. Preprint at https://arxiv.org/abs/1609.04468 (2016).
31. Shoemake, K. Animating rotation with quaternion curves. In *Proceedings of the 12th annual conference on Computer graphics and interactive techniques* 245–254 (1985).
32. Yang, S.-Y. Pharmacophore modeling and applications in drug discovery: Challenges and recent advances. *Drug Discov. Today* **15**, 444–450 (2010).
33. Zou, J. et al. Towards more accurate pharmacophore modeling: Multicomplex-based comprehensive pharmacophore map and most-frequent-feature pharmacophore model of CDK2. *J. Mol. Graph. Model* **27**, 430–438 (2008).
34. Berman, H. M. et al. The protein data bank. *Nucl. Acids Res.* **28**, 235–242 (2000).
35. Ladbury, J. E. Just add water! The effect of water on the specificity of proteinligand binding sites and its potential application to drug design. *Chem. Biol.* **3**, 973–980 (1996).
36. Marti-Renom, M. A. et al. Comparative protein structure modeling of genes and genomes. *Annu. Rev. Biophys. Biomol. Struct.* **29**, 291–325 (2000).
37. Wolber, G. & Langer, T. LigandScout: 3-D pharmacophores derived from proteinbound ligands and their use as virtual screening filters. *J. Chem. Inf. Model.* **45**, 160–169 (2005).
38. Kurogi, Y. & Güner, O. F. Pharmacophore modeling and three-dimensional database searching for drug design using catalyst. *Curr. Med. Chem.* **8**, 1035–1055 (2001).
39. Xie, T. et al. Structural basis of RIP1 inhibition by necrostatins. *Structure* **21**, 493–499 (2013).
40. Fayaz, S. M. & Rajanikant G. K. Ensemble pharmacophore meets ensemble docking: A novel screening strategy for the identification of RIPK1 inhibitors. *J. Comput. Aided Mol. Des.* **28**,

779–794 (2014).

41. Fayaz, S. M. & Rajanikant G. K. Ensembling and filtering: An effective and rapid in silico multitarget drug-design strategy to identify RIPK1 and RIPK3 inhibitors. *J. Mol. Model* **21**, 314 (2015).
