# Supplementary material for: Generative deep learning enables the discovery of a potent and selective RIPK1 inhibitor
Source: Nat Commun. 2022 Nov 12;13:6891. doi: 10.1038/s41467-022-34692-w (PMC9653409; doi:10.1038/s41467-022-34692-w)
Supplement: Supplementary file 3 — Description of Additional Supplementary Files [file 41467_2022_34692_MOESM3_ESM.pdf]

Description of Additional Supplementary Files

Supplementary Software: Computer codes of the GDL model.
