# Supplementary material for: Generative deep learning enables the discovery of a potent and selective RIPK1 inhibitor
Source: Nat Commun. 2022 Nov 12;13:6891. doi: 10.1038/s41467-022-34692-w (PMC9653409; doi:10.1038/s41467-022-34692-w)
Supplement: Supplementary file 5 — Reporting Summary [file 41467_2022_34692_MOESM5_ESM.pdf]

nature portfolio

Corresponding author(s): Shengyong Yang

Last updated by author(s): Oct 25, 2022

Reporting Summary

Nature Portfolio wishes to improve the reproducibility of the work that we publish. This form provides structure for consistency and transparency in reporting. For further information on Nature Portfolio policies, see our Editorial Policies and the Editorial Policy Checklist.

Statistics

For all statistical analyses, confirm that the following items are present in the figure legend, table legend, main text, or Methods section.

n/a	Confirmed	
☐	☒	The exact sample size (*n*) for each experimental group/condition, given as a discrete number and unit of measurement
☐	☒	A statement on whether measurements were taken from distinct samples or whether the same sample was measured repeatedly
☐	☒	The statistical test(s) used AND whether they are one- or two-sided<br>*Only common tests should be described solely by name; describe more complex techniques in the Methods section.*
☒	☐	A description of all covariates tested
☒	☐	A description of any assumptions or corrections, such as tests of normality and adjustment for multiple comparisons
☐	☒	A full description of the statistical parameters including central tendency (e.g. means) or other basic estimates (e.g. regression coefficient) AND variation (e.g. standard deviation) or associated estimates of uncertainty (e.g. confidence intervals)
☐	☒	For null hypothesis testing, the test statistic (e.g. *F*, *t*, *r*) with confidence intervals, effect sizes, degrees of freedom and *P* value noted<br>*Give P values as exact values whenever suitable.*
☒	☐	For Bayesian analysis, information on the choice of priors and Markov chain Monte Carlo settings
☒	☐	For hierarchical and complex designs, identification of the appropriate level for tests and full reporting of outcomes
☒	☐	Estimates of effect sizes (e.g. Cohen's *d*, Pearson's *r*), indicating how they were calculated

Our web collection on statistics for biologists contains articles on many of the points above.

Software and code

Policy information about availability of computer code

Data collection	CLARIOstar (v5.61); Eclipse Ci-L microscope; Pannoramic MIDI scanner; FUSION-FX6.EDGE V.070; python 3.6.9; tensorflow 1.10.0; numpy 1.15.4; rdkit 2019.09.2.0; Computer codes of our GDL model are provided as Supplementary Software and have been deposited in the Zenodo under accession code doi: 10.5281/zenodo.7074218 [https://doi.org/10.5281/zenodo.7074218].
Data analysis	GraphPad Prism (v 8.00); HKL2000;CCP4 (version 7.0.078); Coot (version 0.8.9.2); Phenix(Version 1.18.2_3874); CaseViewer2.4; python 3.6.9; tensorflow 1.10.0; numpy 1.15.4; rdkit 2019.09.2.0; umap-learn 0.4.6; seaborn 0.11.1; tmap 1.0.4; faerun 0.3.20; Discovery Studio 3.1

For manuscripts utilizing custom algorithms or software that are central to the research but not yet described in published literature, software must be made available to editors and reviewers. We strongly encourage code deposition in a community repository (e.g. GitHub). See the Nature Portfolio guidelines for submitting code & software for further information.

Data

Policy information about availability of data

All manuscripts must include a data availability statement. This statement should provide the following information, where applicable:

- Accession codes, unique identifiers, or web links for publicly available datasets
- A description of any restrictions on data availability
- For clinical datasets or third party data, please ensure that the statement adheres to our policy

The SDF file of the generated data has been deposited in the Zenodo under accession code doi: 10.5281/zenodo.6451205 [https://doi.org/10.5281/

zenodo.6451205].
The crystal structure of the RIPK1–RI-962 complex has been deposited in the Protein Data Bank (PDB) under accession code 7YDX [http://doi.org/10.2210/pdb7YDX/pdb].
The crystal structures of RIPK1 used in this study are available in the Protein Data Bank (PDB) under accession code 4ITJ [http://doi.org/10.2210/pdb4ITJ/pdb], 4ITI [http://doi.org/10.2210/pdb4ITI/pdb], 4ITH [http://doi.org/10.2210/pdb4ITH/pdb], 4NEU [http://doi.org/10.2210/pdb4NEU/pdb], 5HX6 [http://doi.org/10.2210/pdb5HX6/pdb], 5TX5 [http://doi.org/10.2210/pdb5TX5/pdb], 6C4D [http://doi.org/10.2210/pdb6C4D/pdb], 6HHO [http://doi.org/10.2210/pdb6HHO/pdb], 6NW2 [http://doi.org/10.2210/pdb6NW2/pdb], 6R5F [http://doi.org/10.2210/pdb6R5F/pdb], 6OCQ [http://doi.org/10.2210/pdb6OCQ/pdb], 6NYH [http://doi.org/10.2210/pdb6NYH/pdb] and 6RLN [http://doi.org/10.2210/pdb6RLN/pdb].
All other data that support the conclusions are available from this published article, supplementary information files, Source Data file or corresponding authors on reasonable request.
Source data are provided with this paper.

Human research participants

Policy information about studies involving human research participants and Sex and Gender in Research.

Reporting on sex and gender	Not Applicable
Population characteristics	Not Applicable
Recruitment	Not Applicable
Ethics oversight	Not Applicable

Note that full information on the approval of the study protocol must also be provided in the manuscript.

Field-specific reporting

Please select the one below that is the best fit for your research. If you are not sure, read the appropriate sections before making your selection.

☒ Life sciences ☐ Behavioural & social sciences ☐ Ecological, evolutionary & environmental sciences

For a reference copy of the document with all sections, see nature.com/documents/nr-reporting-summary-flat.pdf

Life sciences study design

All studies must disclose on these points even when the disclosure is negative.

Sample size	Sample size estimation was not relevant for this study, as it does not report on a statistical evaluation of effects between two or more groups. Sample-size calculations were not done. For the animal study , the numbers of animals in each group meet the requirement for statistical analysis (at least 3 for each group), which is sufficient given the excellent technical reproducibility. For experiments other than animal studies, at least three samples were selected to meet the requirements for statistical analysis.
Data exclusions	No data has been excluded from the analyses presented in this manuscript.
Replication	In cell experiments, to ensure reproducibility of experimental findings, each assay was performed at least two times to confirm the results. IC50 measurements were carried out with two or three biological replicates for each data point and these data were used to calculate mean values.<br>In animal studies, multiple mice were included in each group. The SIRS model experiment and IBD model experiment were repeated twice. Reproducible findings were obtained from all repeats.
Randomization	For all in vivo experiments, sex- and age-matched mice were randomized into different experimental groups. Mice were randomly assigned to vehicle and treatment groups. For in vitro experiments, cells were randomized into different control/treatment groups.The tests were also randomly selected from all samples. The pictures were representatively shown.
Blinding	For the measurement of quantitative values, such as colon length, Cytokine content, body temperature, body weight and survival, data acquiring does not involve subjective judgments, therefore no blinding procedures were applied to the experimentalists involved. For the in vitro and in vivo experiments, blinding was not possible because researchers were involved in induction/treatment procedures. For the histological examinations, qualified pathologists were blinded to group allocation to ensure the assessment was unbiased.

Reporting for specific materials, systems and methods

We require information from authors about some types of materials, experimental systems and methods used in many studies. Here, indicate whether each material, system or method listed is relevant to your study. If you are not sure if a list item applies to your research, read the appropriate section before selecting a response.

Materials & experimental systems

n/a	Involved in the study
☐	☒ Antibodies
☐	☒ Eukaryotic cell lines
☒	☐ Palaeontology and archaeology
☐	☒ Animals and other organisms
☒	☐ Clinical data
☒	☐ Dual use research of concern

Methods

n/a	Involved in the study
☒	☐ ChIP-seq
☒	☐ Flow cytometry
☒	☐ MRI-based neuroimaging

Antibodies

Antibodies used

(1) human RIPK1 antibody (R&D, 334640; , 1:1000)
(2) mouse RIPK1 antibody (Affinity, DF2642, 1:1000)
(3) human phospho-RIP (Ser166) rabbit mAb (Cell Signaling Technologies, 65746, 1:1000)
(4) mouse phospho-RIP (Ser321) rabbit mAb (Cell Signaling Technologies, 38662, 1:1000)
(5) human RIPK3 (B-2) antibody (Santa Cruz, sc-374639, 1:250)
(6) mouse RIPK3 antibody (Abcam, ab62344, 1:1000)
(7) human anti-RIP3 (phospho S227) antibody (Abcam, ab209384, 1:2000)
(8) mouse anti-RIP3 (phospho T231 + S232) antibody (Abcam, ab205421, 1:500)
(9) anti-MLKL (58-70) antibody (Sigma, M6697, 1:250)
(10) human anti-MLKL (phospho S358) antibody (Abcam, ab187091, 1:1000)
(11) mouse anti-MLKL (phospho S345) antibody (Abcam, ab196436, 1:1000)
(12) β-actin (Proteintech, 66009-1-Ig, 1:1000)
(13) S100A9 (D3U8M) rabbit mAb (Cell Signaling Technologies, 73425,1:800)
(14) HRP-conjugated Affinipure Goat Anti-Rabbit IgG(H+L) (Proteintech, SA00001-2, 1:5000)
(15) HRP-conjugated Affinipure Goat Anti-Mouse IgG(H+L) (Proteintech, SA00001-1, 1:5000)

Validation

All antibodies were commercially available, and validated by manufacturers and/or citations. Manufacturer websites containing their validation data and/or citations, are listed below:
(1) human RIPK1 antibody (R&D, 334640; , 1:1000): website(https://www.rndsystems.com/cn/products/human-mouse-rat-ripk1-rip1-antibody-334640_mab3585) and citations (PMID: 35045357, etc).
(2) mouse RIPK1 antibody (Affinity, DF2642, 1:1000): website(http://www.affbiotech.cn/goods-6723-DF2642-RIPK1_Antibody.html).
(3) human phospho-RIP (Ser166) rabbit mAb (Cell Signaling Technologies, 65746, 1:1000): website(https://www.cellsignal.cn/products/primary-antibodies/phospho-rip-ser166-d1l3s-rabbit-mab/65746?_=1666703008939&Ntt=65746&tahead=true).
(4) mouse phospho-RIP (Ser321) rabbit mAb (Cell Signaling Technologies, 38662, 1:1000): website(https://www.cellsignal.cn/products/primary-antibodies/phospho-rip-ser321-e9k2a-rabbit-mab/38662?_=1666704015062&Ntt=38662&tahead=true).
(5) human RIPK3 (B-2) antibody (Santa Cruz, sc-374639, 1:250): website(https://www.scbt.com/p/rip3-antibody-b-2?requestFrom=search) and citations (PMID: 36155057, etc).
(6) mouse RIPK3 antibody (Abcam, ab62344, 1:1000): website(https://www.abcam.cn/rip3-antibody-ab62344.html) and citations (PMID: 33241577, etc).
(7) human anti-RIP3 (phospho S227) antibody (Abcam, ab209384, 1:2000): website(https://www.abcam.cn/nav/primary-antibodies/rabbit-monoclonal-antibodies/rip3-phospho-s227-antibody-epr9627-ab209384.html) and citations (PMID: 33369872, etc).
(8) mouse anti-RIP3 (phospho T231 + S232) antibody (Abcam, ab205421, 1:500): website(https://www.abcam.cn/rip3-phospho-t231--s232-antibody-2d7-ab205421.html) and citations (PMID: 33605079, etc).
(9) anti-MLKL (58-70) antibody (Sigma, M6697, 1:250): website(https://www.sigmaaldrich.cn/CN/zh/product/sigma/m6697) and citations (PMID: 30062059, etc).
(10) human anti-MLKL (phospho S358) antibody (Abcam, ab187091, 1:1000): website(https://www.abcam.cn/mlkl-phospho-s358-antibody-epr9514-ab187091.html) and citations (PMID: 33503908, etc).
(11) mouse anti-MLKL (phospho S345) antibody (Abcam, ab196436, 1:1000): website(https://www.abcam.cn/mlkl-phospho-s345-antibody-epr95152-ab196436.html) and citations (PMID: 32999468, etc).
(12) β-actin (Proteintech, 66009-1-Ig, 1:1000): website(https://www.ptgcn.com/products/Pan-Actin-Antibody-66009-1-Ig.htm#publications) and citations (PMID: 32581380, etc).
(13) S100A9 (D3U8M) rabbit mAb (Cell Signaling Technologies, 73425,1:800): website(https://www.cellsignal.cn/products/primary-antibodies/s100a9-d3u8m-rabbit-mab-rodent-specific/73425?site-search-type=Products&N=4294956287&Ntt=73425&fromPage=plp&_requestid=5298198).

Eukaryotic cell lines

Policy information about cell lines and Sex and Gender in Research

Cell line source(s)	HT-29, U937, L929, HEK 293T ,J774A.1 and SF9 cells were purchased from the American Type Culture Collection.
Authentication	All the cell lines were commercially available and have not been authenticated after receiving them.
Mycoplasma contamination	Mycoplasma testing confirmed negative at regular intervals.
Commonly misidentified lines (See ICLAC register)	No commonly misidentified cell lines were used.

Animals and other research organisms

Policy information about studies involving animals; ARRIVE guidelines recommended for reporting animal research, and Sex and Gender in Research

Laboratory animals	Male SD rats (n = 3 per group, aged 6-8 weeks and weighting 180-250 g) were used to pharmacokinetic (PK) studies. C57BL/6 mice (age: 6-8 weeks) consist of half male (weight: 20-23 g) and half female (weight: 18-20 g) were used to assess toxicity of RI-962 in vivo. C57BL/6 mice, aged from 6-8 weeks, were used to in vivo studies. The mice were kept in cages with individual ventilation under 65% humidity and an ambient temperature of 21–23°C and a 12h–12h day–night cycle for housing and husbandry.
Wild animals	The study did not involve wild animals.
Reporting on sex	Animals were randomized at a 1:1 male/female ratio in the chronic toxicity experiment.<br>Sex was not considered in other study design.<br>The sex of the animals used for each experiments were reported in the figure legends and methods.
Field-collected samples	The study did not involve samples collected from the field.
Ethics oversight	All procedures related to animal handling, care and treatment in pharmacokinetic (PK) studies were performed according to the guidelines approved by the Institute Animal Care and Use Committee (IACUC) of Shanghai Medicilon Inc.<br>All procedures related to animal handling, care and treatment in in vivo toxicity and efficacy studies were performed according to the guidelines approved by the IACUC of West China Hospital, Sichuan University (20211062A).

Note that full information on the approval of the study protocol must also be provided in the manuscript.
